# Exploring the feasible net-zero transition pathway in China considering energy system flexibility

**DOI:** 10.1038/s41467-026-71410-2

**Published:** 2026-04-11

**Authors:** Shu Zhang, Wenying Chen

**Affiliations:** 1 Research Center for Contemporary Management, Tsinghua University, Beijing, China; 2 Institute of Energy, Environment and Economy, Tsinghua University, Beijing, China

**Keywords:** Environmental social sciences, Environmental sciences, Energy science and technology, Energy and society, Energy infrastructure

## Abstract

Net-zero energy transition is projected to accelerate the replacement of fossil fuels with renewables, leaving system flexibility resources increasingly scarce. Here, we present a sub-annual energy-environment-economy model with endogenous hourly energy demand profiles and power balance dynamics including power dispatch, storage operations and demand-side response that co-optimizes supply- and demand-side flexibility, to map feasible transition pathways for China. The results show that compared with coarser timeslice representative of common modelling practice, sub-annual representation tightening flexibility needs with a high variable renewable energy and high electrification energy system. Results reveal steeper load ramps and frequent evening price strikes, identify increased activities in thermal power, nuclear power, hydrogen production, and energy storage. Accounting for temporal variability in supply and demand, the cost-optimal solution exhibits marginal abatement costs that are over 9% higher, but incorporating demand-side flexibility measures can mitigate cost growth and delineates least-regret portfolios for reliable, affordable decarbonization. Incentives for demand-side response such as load time-shifting and vehicle-to-grid can reduce investment in pumped hydro by 23% and yield more than a threefold cost-benefit ratio. The study highlights enhanced modelling of temporal dynamics within future energy model development and incentive-compatible market mechanism design for dispatchable resource development.

## Abstract (web summary)

Based on the hourly IAM modelling, the study finds accounting for sub-annual dynamics tightens China’s net-zero transition feasibility. Demand-side flexibility, such as load time-shifting and V2G, reduces storage needs and boosts cost-effectiveness.

## Introduction

In alignment with the Paris Agreement, key nations have updated their nationally determined contributions (NDCs) and declared strategies targeting mid-century net-zero emissions, necessitating swift energy system transition^1^. China, the top emitter of greenhouse gases in total amount^2^, is undergoing a rapid transformation, driven by the massive variable renewable energy (VRE) deployment and accelerated electrification. The increased variability in supply and demand is making the flexibility challenge more urgent for the purposes of enhancing energy security and facilitating the low-carbon transition^3–7^. Fragmented regulatory frameworks limited economic incentives and insufficient market mechanisms pose significant obstacles to the redistribution of load in a manner that aligns with power output, consequently constraining system flexibility^8^. Failing to adequately quantify system flexibility may result in the misallocation of resources toward unfeasible or misguided endeavors.

Multi-sector energy system model and integrated Assessment Model (IAM) are designed to provide a quantitative description of coupled human and natural system processes and their interactions, having been widely applied in long-term planning studies in energy and broader domains. Some national energy system model framework, such as TIMES-family, employ coarse sub-annual timeslices (timeslices by season and daily period)^9^, and several global IAMs operate at annual with limited operational detail, although optional timeslices exist^10,11^. Coarse timeslice modeling, however, often cannot fully capture diurnal ramps, peak load hours, or the operational synergies from demand-side flexibility. Without considering the challenges of power system flexibility, their proposed transition pathways casts doubt on the practicability^12–18^. The power system-focused models have detailed temporal resolution but fall short in projecting load patterns due to the lack of a comprehensive energy system perspective, consequently necessitating the external setting of future load projections^19–26^. Some studies have attempted to link IAMs to power system models. While these studies have partially addressed shortcomings associated with temporal resolution, they have encountered difficulties in accurately portraying the intricate interactions between energy storage, sector coupling, and demand-side response, which are recognized as critical to energy system flexibility^27–31^. Building hierarchically nested representative timeslices within the IAM framework facilitates the depiction of transition dynamics with higher temporal resolution, concurrently avoiding unnecessary computational requirements^32–34^. However, most studies concentrate on power dispatching, ignoring the immense potential within energy demand sectors^35–37^.

In this study, we enhanced the modeling of temporal dynamics in an energy-environment-economy model, China TIMES 2.0, using 56 representative timeslices nested at the annual-seasonal-weekly-daily-hourly to assess energy transition under operational constraints and flexibility enhancement measures. This approach establishes an integrated framework that aligns long-term strategic planning with the complexities of short-term operational dynamics. Based on industry-level energy consumption pattern modeling, the amplitude and shape of the power load curve are optimized endogenously. Additionally, simulations encompass the functioning of energy storage, variations in energy service demands across diverse time scales, and the application of demand-side response tools, including load time-shifting, orderly electric vehicle (EV) charging, and vehicle-to-grid (V2G) integration. Our findings indicate that effective coordination between orderly energy consumption, energy storage and power dispatching are pivotal in rectifying demand-supply imbalances. Rapidly rising electricity demand for EV charging and hydrogen production from renewables (green hydrogen) should be directed to accommodate fluctuations in renewable energy generation. Active demand-side response strategies such as V2G and load time-shifting can significantly curtail peak load and alleviate ramping issues, thereby circumventing the need for energy storage investment, and preventing volatile price oscillations. This signifies the key in subsidizing demand-side response measures.

## Results

### Assessing the energy transition at the sub-annual level

**Fig. 1:**
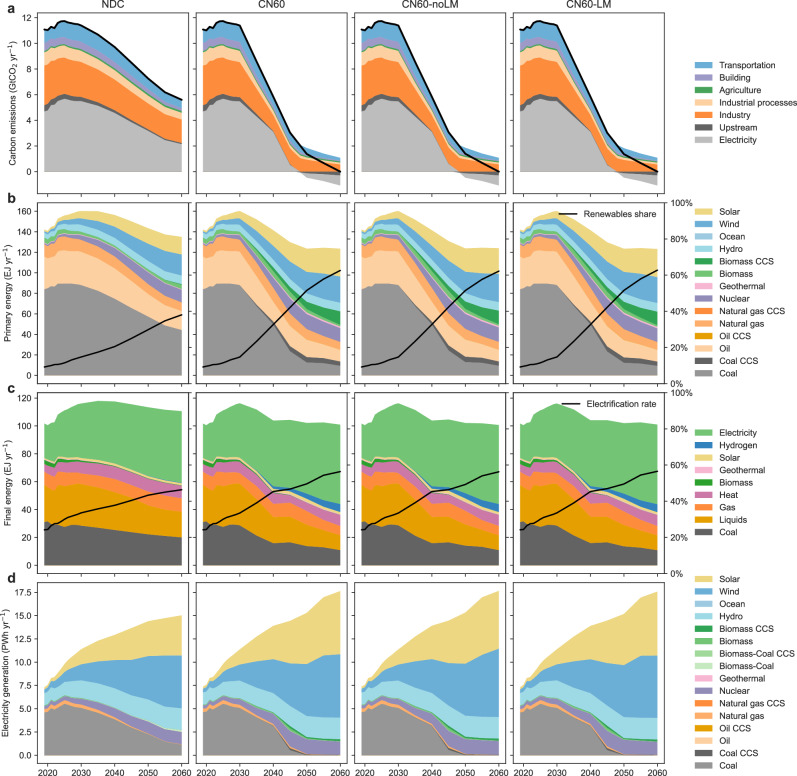
China’s energy system decarbonization pathway. **a** Fossil fuel and industrial processes (FFI) CO_2_ emissions by sector, **b** Primary energy mix by fuel, **c** Final energy mix by fuel, **d** Electricity generation by technology. In the legend of Panel **b**, the fuel names ending in CCS indicate fuels are used by processes equipped with carbon capture and storage (CCS) and otherwise indicate processes without CCS. In the legend of Panel **d**, the fuel names ending in CCS indicate power plants equipped with CCS and otherwise indicate power plants without CCS. GtCO_2_ yr^-1^ represents billion tons of CO_2_ per year. EJ yr^-1^ represents exajoule per year. PWh yr^-1^ represents petawatt-hour per year.

China aims for net-zero emissions by 2060 amid rising energy demand and sectoral transition (Fig. 1). China is projected to peak its fossil fuel and industrial CO₂ emissions between 2025 and 2030, then carbon emissions will decrease rapidly to achieve carbon neutrality by 2060. Coal use will decline sharply post-2030, with renewables dominating power generation after 2035. In this study, we propose four hourly scenarios with different flexibility management measures and climate ambition: NDC, CN60, CN60-noLM, and CN60-LM, which represent the baseline reference, the net-zero scenario with orderly energy consumption (flexible load adjustment for EV charging and hydrogen production), the net-zero scenario maintaining current energy consumption patterns and the net-zero scenario with active supply-demand interactions (introduction of demand-side response measures), respectively. For comparison, we also present three scenarios considering coarser temporal resolutions: CN60-noTS, CN60-DN, and CN60-noHour, corresponding to no sub-annual modeling (most multi-sector energy system models in China), only considering day and night timeslices (China TIMES 1.0 model), and considering seasonal-weekly-daily modeling but without hourly modeling (common practice in national TIMES models).

**Fig. 2:**
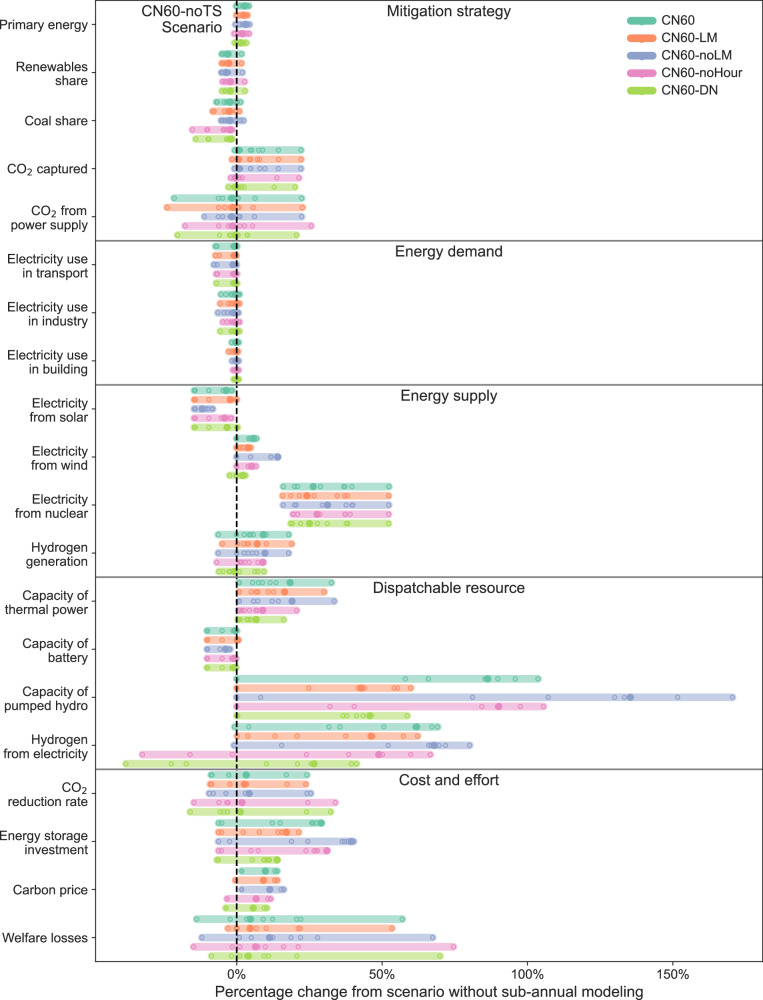
Impact of sub-annual modeling on energy system transition. The number represents the percent change relative to the scenario without sub-annual modeling in 2060. The solid dots represent the year 2060 and the other eight hollow dots represent the years 2035, 2040, 2045, 2050, 2055, 2070, 2085, 2100.

**Fig. 3:**
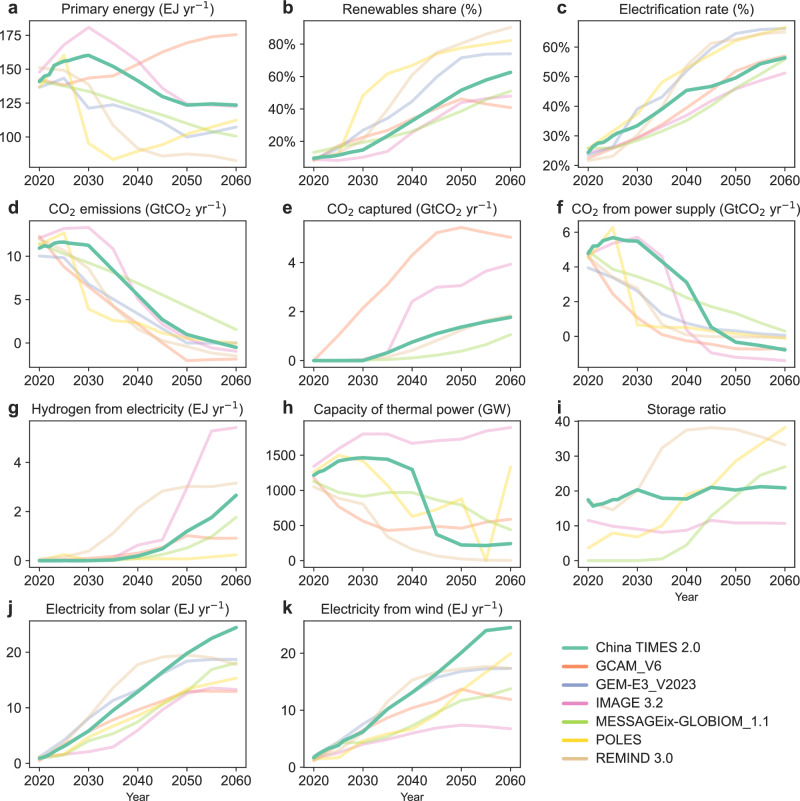
Multi-model comparison for China’s net-zero target. **a** Primary energy supply, **b** Renewable energy share in primary energy supply, **c** Electricity share in final energy consumption, **d** Total CO_2_ emissions, **e** CO_2_ captured, **f** CO_2_ emissions from power sector, **g** Hydrogen generation through electrolysis, **h** Installed capacity of thermal power, **i** The ratio of installed energy storage capacity to wind and solar generation (gigawatt for capacity per exajoule for power generation), **j** Electricity generation from solar, **k** Electricity generation from wind. Based on Glasgow+ scenario from ENGAGE project protocol, the dominant integrated assessment models simulated net-zero scenarios. Except for China TIMES 2.0 (CN60 scenario), all other models are at the annual level. In the Panel **i**, the capacity units for energy storage are all transformed to GW assuming 2-hour energy storage. EJ yr^-1^ represents exajoule per year. GtCO_2_ yr^-1^ represents billion tons of CO_2_ per year. GW represents gigawatt.

Figure 2 compares outcomes under different temporal granularity and flexibility management across mitigation strategy, energy demand, energy supply, dispatchable resources, and cost. Figure 3 compares the results with prominent IAMs under a harmonized scenario framework with the same carbon budget constraints and net-zero targets. The finer temporal resolution reveals operational constraints that are not visible at coarse resolution which tighten feasible pathways for high VRE shares and fast electrification unless flexibility is enhanced accordingly. In addition, the distinct trajectories of thermal power, green hydrogen, and energy storage in China TIMES 2.0, which differ from other IAMs, clearly demonstrate the unique impact of system flexibility on long-term planning. We identify renewable resource curtailment and load shedding might both occur due to supply-demand mismatches. By 2060, electricity consumption in the transportation, industrial, and building sectors would decline by up to 7%, 5%, and 2%, respectively, compared to the annual scenario. When temporal variability is explicitly represented, the cost-optimal solution exhibits marginal abatement costs that are over 9% higher and total final energy about 3% lower by 2060 relative to a coarse-timeslice baseline. These differences arise because the model internalizes flexibility requirements and operational constraints that are muted at the annual scenario.

The discrepancies in the technology portfolio are more pronounced than those observed in aggregated pathways. Due to the difficulty of VRE integration, fossil fuel with carbon capture and storage (CCS) would play a prominent role before net-zero is achieved. Thermal power capacity in 2055 could increase by 16-21% in the CN60-DN and CN60-noHour scenarios compared to the CN60-noTS scenario. For hourly scenarios, the increase would be 30-34%. From the hourly perspective, we identify thermal power achieves a shift from electricity provider to flexibility provider, mitigating the possible shocks of rapid decommissioning. On the other hand, renewables proportion among energy mix would decrease by 2.2% for CN60-nohour and 3.0% for CN60. Comparing the three hourly scenarios, the demand-side measures have cross-sectoral impacts, in particular significant impacts on power transformation and energy storage development. The coarse time granularity masks the impact of different flexibility measures. Under the CN60-LM scenario, which considers active demand-side flexibility, photovoltaics (PV) generation would decrease by 2% compared to the annual-scale model, while in the CN60-noLM scenario, which does not include demand-side flexibility measures, PV generation would decrease by 12%. For scenarios considering 2-timeslice (CN60-DN) and 32-timeslice (CN60-noHour) scenarios, PV generation would decrease by 3% and 4%, respectively. Demand for dispatchable resources would rise sharply with considerable differences between scenarios, with pumped hydro growing by 43-135% under hourly scenarios, and CN60-noHour and CN60-DN would grow by 90% and 46% respectively. Grid-connected hydrogen production would increase by more than 45% when seasonal factors are taken into account, but only by 27% when only diurnal variations are considered.

### Flexibility scarcity leading to power plant repositioning

**Fig. 4:**
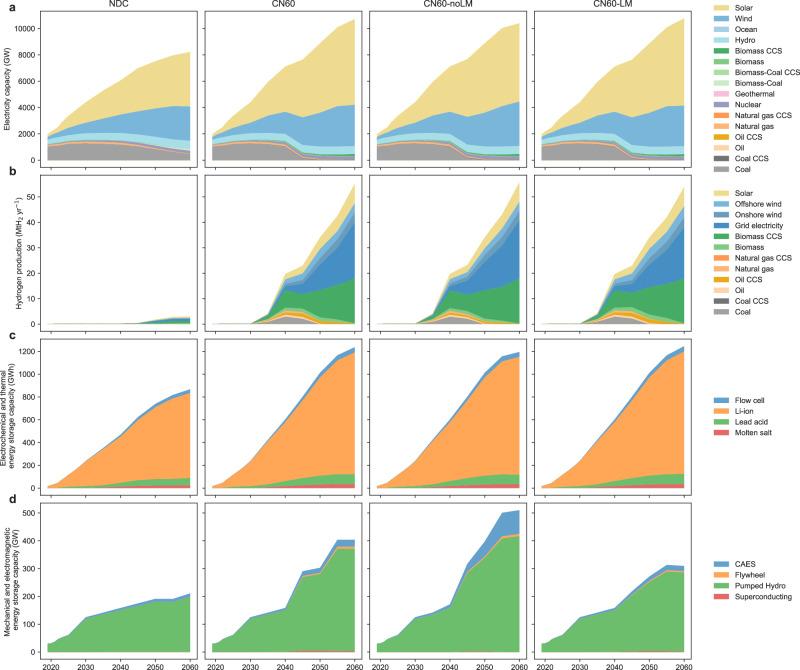
The installed capacity of the energy supply sector to provide energy system flexibility. **a** Electricity generation capacity by technology, **b** Hydrogen production capacity by technology, **c** Electrochemical and thermal energy storage capacity by technology, **d** Mechanical and electromagnetic energy storage capacity by technology. In the legend of Panel **a**, the fuel names ending in CCS indicate power plants equipped with carbon capture and storage (CCS) and otherwise indicate power plants without CCS. In the legend of Panel **b**, the fuel names ending in CCS indicate hydrogen generations equipped with CCS and otherwise indicate hydrogen generations without CCS. GW represents gigawatt. MtH_2_ yr^-1^ represents million tons of hydrogen per year. GWh represents gigawatts-hour.

During periods of ramping, generation output variations, net load fluctuations, and peak load hours, flexibility scarcity issues are likely to arise. China has historically concentrated its flexibility resources on the supply side, primarily through thermal power and hydropower executing dispatch instructions. Risks associated with system flexibility are progressively accumulating, particularly following the expedited decommissioning of thermal power plants after 2040. Under the CN60 scenario, an annual coal-fired power plant (CFPP) retirement averages 26 gigawatts (GW) during the 2030-2040. This rate quadruples during 2040-2050, with almost all unabated CFPPs phased out by 2050 (Fig. 4a). As VRE expands, the proportion of dispatchable units will gradually decrease, thereby weakening the system flexibility. CFPP flexibility retrofits can significantly improve the dispatch performance of existing assets, partially alleviating the lack of flexibility (Supplementary Notes 2 and Supplementary Figs. 16 and 17). The net load curve, the difference between the real load and VRE output, which is an indicator of power system flexibility, would be negative at noon after 2050 with two increasingly steep load ramps (Supplementary Fig. 6).

**Fig. 5:**
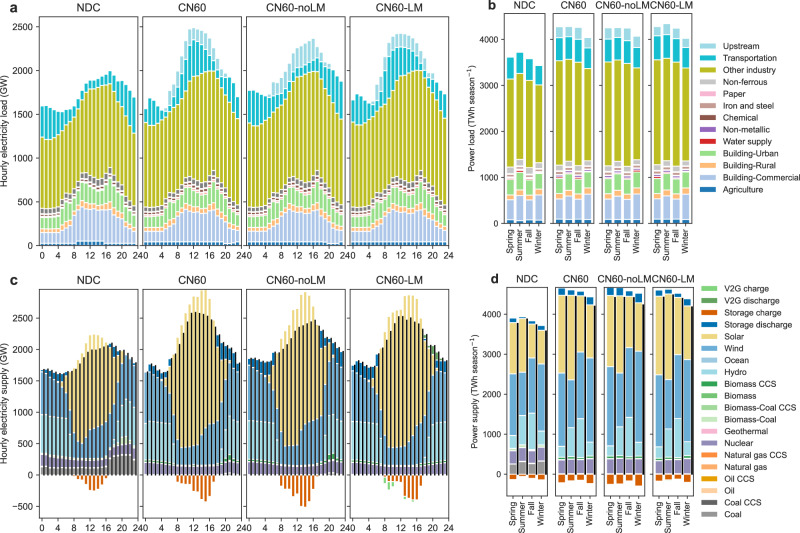
Seasonal and intra-day variation in electricity supply and demand in 2060. **a** Electricity consumption by industry for a summer workday, **b** Electricity consumption by industry in different seasons, **c** Electricity generation by technology for a summer workday, **d** Electricity generation by technology in different seasons. In the legend of Panel **c** and **d**, the fuel names ending in CCS indicate power plants equipped with carbon capture and storage (CCS) and otherwise indicate power plants without CCS. GW represents gigawatt. TWh season^-1^ represents terawatt-hour per season. The data for other seasons and working days can be found at Supplementary Figs. 7 and 8.

With the rapid spread of VRE, it is essential to rapidly scale up low-carbon flexibility resources. Nuclear, hydro and bioenergy with CCS (BECCS) power are expected to become increasingly valuable dispatchable resources of responding to rapid power load and supply fluctuations (Fig. 5c). Nuclear power, traditionally employed to meet baseload, but its flexible operation has gained momentum in both research^38^ and policy^39^ perspectives. Hydropower has gained significant importance in the high-VRE power system and is poised to become a central pillar of flexibility. Its operational focus is anticipated to transition from catering to peak loads to bolstering ramping capacity and accommodating nighttime loads, with over 35% of electricity demand at night being met by hydro.

Seasonal pattens in PV and wind output reshape system balance configuration (Fig. 5d). In summer, PV will dominate, requiring massive electricity storage during midday hours and low-load operation capabilities for thermal power plants. In winter, wind dominance will increase intraday power load fluctuations and multi-hour deficits. These dynamics will increase the demand for fast-ramping capacity, short-duration storage, and demand flexibility to contain curtailment and maintain adequacy.

### Orderly demand easing power imbalance

It is anticipated that there will be an increase in total electricity consumption, rising from 7.6 petawatt-hours (PWh) in 2020 to 17.6 PWh by 2060 (Fig. 1d). This surge is coupled with an increase in peak load, projected to grow from 1.0 terawatt (TW) in 2019 to at least 2.5 TW in 2060. Peak loads could be even further in case power load fluctuates unpredictably.

**Fig. 6:**
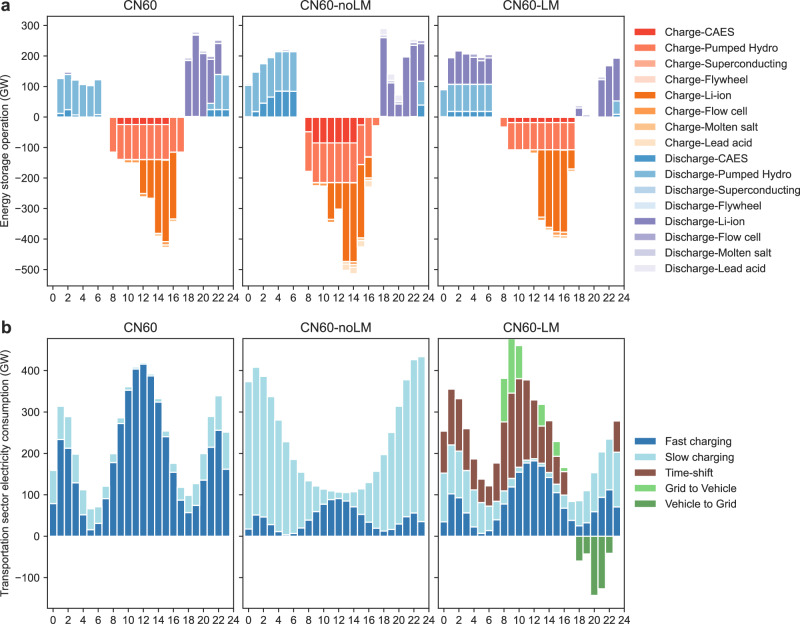
Energy storage, load time-shifting and vehicle-to-grid technology operations 2060. **a** Energy storage operation by technology for a summer workday, **b** Electricity consumption in the transportation sector for a summer workday. CAES represents compressed-air energy storage. GW represents gigawatt. The data for other seasons and working days can be found at Supplementary Figs. 9 and 10.

Our study delineates the seasonal and intraday variations in power loads, a crucial aspect for integrating more challenging-to-dispatch power systems. A thorough examination of power load characteristics is conducted to investigate the flexibility resources embedded in energy consumption patterns. By comparing the CN60 and CN60-noLM scenarios, a discernible shift is evident in the consumption patterns of adaptive loads, including EV charging and hydrogen production. Under the CN60 scenario, results show EV would shift from charging primarily at night to charging during the day, utilizing PV power and alleviating excess electricity supply. Model results suggest adoption of fast-charging devices would increase dramatically, projected to increase from 15% in 2019 to 80% by 2060 in CN60 while remaining stable in CN60-noLM. Comparing year 2060 and year 2020 (Supplementary Fig. 6a), the load profile for CN60 not only shows an increase in value, but also a change in shape from relatively flat to a significant daytime peak, attributed to the effect of orderly load. In the CN60-noLM scenario without demand-side participation, the shape of the load curve remains unchanged, thereby requiring higher storage capacity and activity (Fig. 6a).

**Fig. 7:**
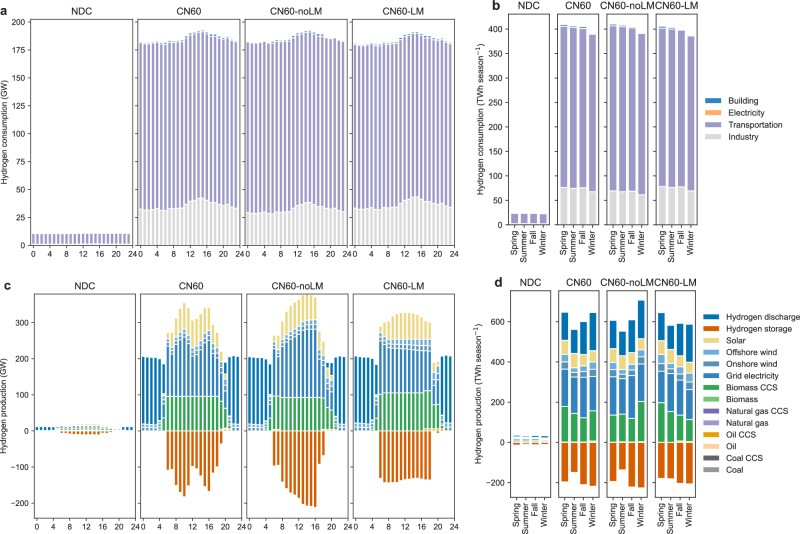
Seasonal and intra-day variation in hydrogen supply and demand in 2060. **a** Hydrogen consumption by industry for a summer workday, **b** Hydrogen consumption by industry in different seasons, **c** Hydrogen production by technology for a summer workday, **d** Hydrogen production by technology in different seasons. In the legend of Panel **c** and **d**, the fuel names ending in CCS indicate hydrogen generations equipped with carbon capture and storage (CCS) and otherwise indicate hydrogen generations without CCS. GW represents gigawatt. TWh season^-1^ represents terawatt-hour per season. The data for other seasons and working days can be found at Supplementary Figs. 11 and 12.

By 2060, hydrogen is projected to account for approximately 7% of final energy consumption in China, with the majority originating from grid-connected hydrogen production facilities if considering flexibility demands (Fig. 7c). Compared with the CN60-noLM scenario, strategic hydrogen production in the CN60 scenario has the potential to utilize surplus daytime electricity, thereby mitigating rather than exacerbating power imbalances during peak demand periods. Furthermore, the conversion and storage of liquefied hydrogen present a viable method for long-duration energy storage.

Space heating and cooling are major contributors to seasonal variations in power load. Nowadays, fossil fuels and district heating (mostly fossil fuels) are the primary sources of the heating demand, while cooling demand is almost entirely met by electricity. Significant increases in winter electricity consumption are expected in the future due to the growing popularity of electric heating technology, thereby narrowing seasonal differences in electricity consumption (Fig. 5b). Hydrogen produced in spring and autumn is stored for use in summer and winter, thereby achieving cross-seasonal energy storage (Fig. 7d).

### Addressing the flexibility crisis through demand-supply interaction

The degree of interaction between supply and demand determines the extent of the flexibility risk. Some demands such as EV charging can be economically optimized by adjusting its energy consumption behavior, and response to price fluctuations. Additional incentives could promote load time-shifting in BF-BOF steelmaking, chemicals, paper and cement industries. The CN60-LM scenario introduces load time-shifting and V2G across all economic sectors with differential incentives (Fig. 6b).

In the CN60-LM scenario, time-shifting and V2G technologies are projected to gain widespread adoption after 2045. Pumped hydro capacity is expected to reach 285 GW by 2060, a level deemed feasible with a consistent annual growth rate of 3% (Fig. 4d). In the CN60 scenario, it is necessary to invest pumped hydro facilities in nearly all eligible candidate sites (368 GW). Even worse, the CN60-noLM scenario exhausts the potential of pumped hydro and requires the intensive construction of other long-term storage facilities (417 GW).

**Fig. 8:**
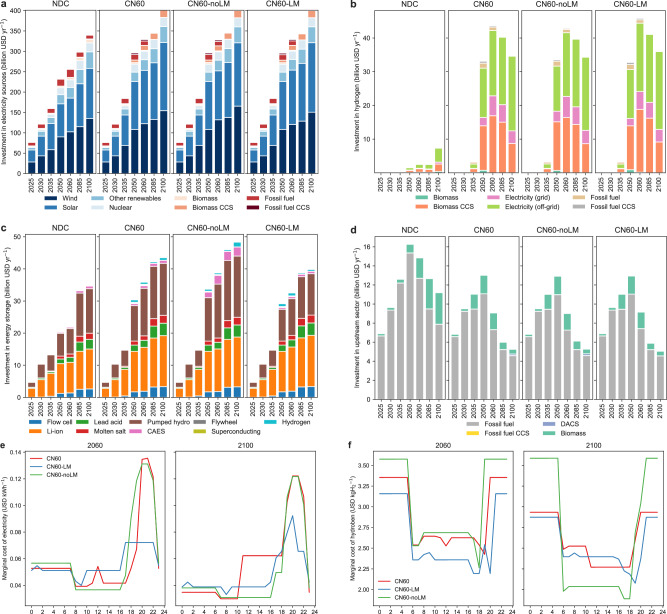
The annual investment required to guarantee system flexibility and the cost of the energy transition. **a** Investment in electricity production by technology, **b** Investment in hydrogen production by technology, **c** Investment in energy storage by technology, **d** Investment in upstream sector by technology, **e** Marginal electricity supply costs for different time periods for a summer workday, **f** Marginal hydrogen supply costs for different time periods for a summer workday. In the legend of Panel **a**, the fuel names ending in CCS indicate power plants equipped with carbon capture and storage (CCS) and otherwise indicate power plants without CCS. In the legend of Panel **b**, the fuel names ending in CCS indicate hydrogen generations equipped with carbon capture and storage (CCS) and otherwise indicate hydrogen generations without CCS. In the legend of Panel **d**, the fuel names ending in CCS indicate energy processing technologies equipped with carbon capture and storage (CCS) and otherwise indicate energy processing technologies without CCS. CAES represents compressed-air energy storage. USD represents US dollar.

V2G technology has emerged as a promising approach for managing peak load. V2G could shift as much as 7% of the peak load in 2060, which would result in substantial reductions in investments required for power balancing and thereby effectively lower the marginal cost of electricity (Fig. 8e). The result of sensitivity analysis of different V2G application rates can be found in Supplementary Notes 3 and Supplementary Figs. 18 and 19.

The energy price increase resulting from emission reduction could incentivize demand-side load reduction, which is also an important means of demand-side response. By 2060, the energy service demands in the industrial sector in the net-zero scenarios are expected to decline by 7–15% compared to the NDC scenario, while transport turnover is projected to decline by 6%, except for aviation and navigation, which would drop by 15%. These figures illustrate the adaptive shifts in production mode and lifestyle in response to transformative pressures.

### Financial implications of a feasible energy transition to net-zero

Considering system operation constraints at the hourly level (CN60), the total system cost would rise by about 4% compared to the CN60-noTS scenario. Enhanced temporal granularity scenarios show more demand on flexibility resources, and the differences between scenarios are mainly in the areas of electricity, hydrogen and energy storage (Supplementary Fig. 13). Net-zero transition in the CN60 scenario requires an increment annual investment by 50% compared to the NDC scenario (Fig. 8). Under the CN60 scenario, annual investment in VRE, energy storage, BECCS and hydrogen needs to be approximately 250, 30, 12 and 44 billion US dollars (USD) by 2060. Energy storage expenses would be 24% larger in the CN60 compared to the CN60-noTS by 2060.

Demand-side response exerts a substantial influence on energy storage expenditures. A 10% reduction in expenses is observed for the CN60-LM scenario relative to the CN60 scenario, while the CN60-noLM scenario demonstrates an expense increase of more than 12% compared to the CN60 scenario. Specifically, the CN60-LM scenario shows a 23% decrease in investment for pumped hydro attributable to active demand-side response measures compared to the CN60 scenario. Furthermore, Incentives for V2G technology, amounting to 4.9 billion USD, could lead to a large saving in power system investment by 14 billion USD.

The marginal costs of electricity show significant variations across different scenarios. The cost fluctuations are more pronounced within the day. Excess power generation during midday hours results in lower marginal costs. In contrast, generation falls sharply in the evening while demand remains high, leading to a spike in prices. More active demand-side response in the CN60-LM scenario can potentially decrease tariff volatility and cut peak hour tariffs in half, dropping from 0.135 to 0.072 USD kWh^-1^.

A significant reduction in the cost of green hydrogen is anticipated. During periods of high solar power availability, green hydrogen price could potentially drop to as low as 2 USD kg^-1^ H_2_, lower than that of conventional hydrogen produced from fossil fuels (gray hydrogen). During nighttime, the cost of green hydrogen is projected to hover 3 USD kg^-1^ H_2_, aligning closely with the cost of fossil fuel-based hydrogen integrated with CCS (blue hydrogen). Based on an estimated 5000 hours of annual operating hours, green hydrogen produced during daylight hours is already cost-effective in the net-zero era, even without considering its contribution to system flexibility.

## Discussion

With the annual-seasonal-weekly-daily-hourly nesting modeling framework, we observe the dynamics of the energy transition with operational constraints. We investigate the feasible pathways for a secure net-zero energy transition by cultivating a more adaptable and integrated energy system. A paradigm shift in the way power system is planned, operated, and financed is necessary to ensure the effective integration of more VRE. Based on our findings, we offer the following insights into demand management, power planning, storage deployment, demand-side response, and market mechanisms for flexibility enhancement and energy system transformation.

Effective demand management is a more economical means of balancing energy supply and demand than investing in power supply. Re-electrification would alter the configuration of the load curve, representing both an opportunity and a challenge for flexibility risks. The booming EV charging and hydrogen production via electrolysis are poised to become valuable resources for managing flexible loads in the coming years. Given that daytime fast charging is a cheap, clean, and flexibility-enhancing option, charging infrastructure construction should gradually increase the fast-charging proportion and guide the coordination of charging load with VRE output through market regulation, policy incentives and intelligent instruments.

For safety scheduling and adequate supply considerations, China has accelerated the approval process for new CFPPs in the past 2 years^40^. Our scenario analyses suggest that existing CFPPs through flexibility retrofits could provide the necessary flexibility for the system for at least 20 years, and after 2040, unabated CFPPs would face considerable mitigation risk under the mounting pressure for carbon reduction. Therefore, enhancing regulation performance of CFPPs and promoting low-carbon retrofits are essential options for mitigating the risk of stranded assets. In a high-VRE power system, hydro, abated CFPPs and BECCS need to reposition and transform into primary flexibility providers.

Diversified energy storage portfolio addresses various flexibility needs. Pumped hydro and compressed-air energy storage (CAES) provide consistent energy supply, large-scale energy storage capacity, and serve as black-start emergency power sources. Electrochemical storage stands out for their ability to maintain grid stability, proving particularly valuable during peak load periods or during ramping. Technologies like flywheels and superconducting storage manage sudden load spikes and abrupt short-term increases in demand. We recommend accelerating pumped hydro construction and promoting variable-speed pumped hydro to effectively manage rapid power fluctuations. We propose to strengthen the integration of the grid-forming energy storage with the development planning of distribution grids, renewables, EVs, to enhance resilience of power grids.

Active demand-side response bridges the gap between energy demand and supply. Demand-side response can considerably impact energy prices and supply-demand balance during periods of limited flexibility resource availability, despite the small share of electricity. Although recent government documents^39,41,42^ have marked the legal recognition of demand-side response as a critical component of demand management, the implementation of market mechanisms for demand-side resources that optimize and coordinate flexibility resources still needs to be piloted and explored.

Conventional objectives in power dispatch are progressively proving ineffective in aligning with the optimal configuration requirements for system flexibility in high-VRE power systems. Future dispatch strategy should prioritize guiding load concentration during periods of abundant VRE resources rather than flattening the load curve.

Current time-of-use tariffs encourage narrowing the peak-to-valley load gap, thereby reducing the need for thermal power regulation for economic efficiency. The tariff mechanism needs to accurately capture the composite value of different generation plants, such as baseload supply, flexible peak supply, and auxiliary standby. More importantly, energy consumers must be able to discern and react to price fluctuations, thereby empowering stakeholders to engage in incentive-compliant actions that foster system flexibility. Specifically, we propose the implementation of the following measures: a) The expansion of the peak-valley price discrepancy, b) The augmentation of the floating range of tariffs. c) The diversification of trading varieties in auxiliary service markets, including ramping and system inertia. d) The establishment of a capacity tariff mechanism for new-type energy storage, rectifying inequitable competition among flexible resources.

Overall, the implementation of sub-annual simulation enables a thorough assessment of both the technical and institutional feasibility of long-term transition pathways, yielding scenarios that offer a more grounded and realistic perspective compared to previous assessment results. The challenges of ensuring power system security during the net-zero transition are evidenced by recent global trends, including more frequent power interruptions, load control, and electricity price volatility. A pressing need has emerged within the broader IAMs community and among energy system modelers to enhanced modelling of sub-annual dynamics and flexibility management measures. This paper leverages flexible potential from both the supply and demand sides of the energy system to create a long-term transition strategy that adheres to the imperative of real-time power balance. While richer simulations of uncertainty and extreme scenarios are difficult to perform due to computational capacity limitations, the insights garnered here provide a nuanced understanding of the multifaceted challenges and prospects pertaining to energy system flexibility in China, offering valuable lessons that could be beneficial for other developing nations navigating towards a net-zero future.

## Methods

### China TIMES 2.0 model overview

**Fig. 9:**
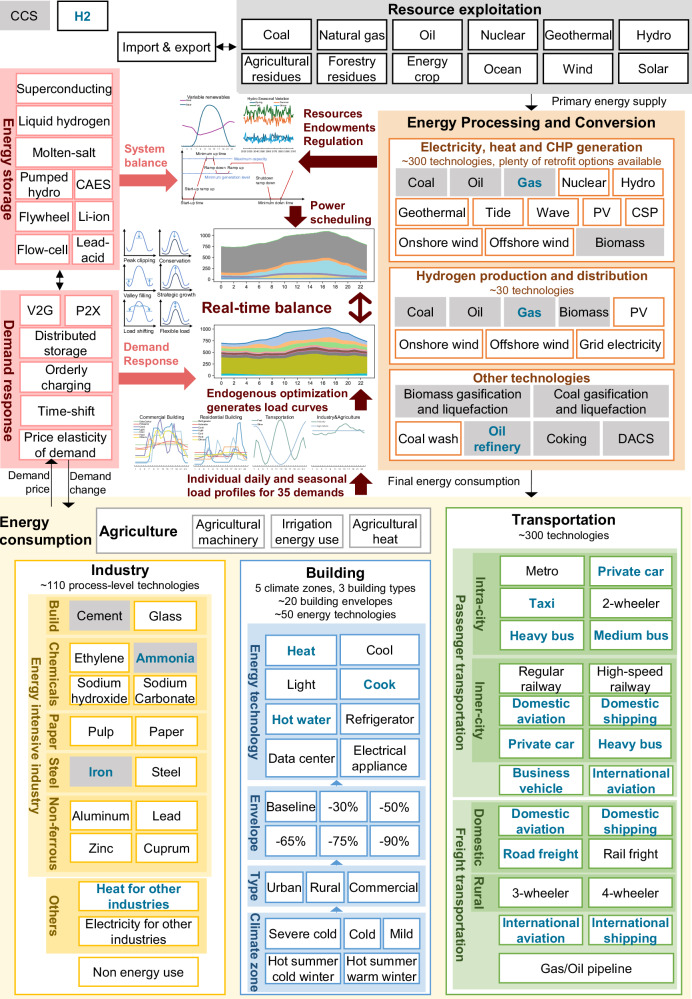
The China TIMES 2.0 model framework. China TIMES 2.0 model covers the full chain of energy system from resource extraction to energy end-use. It includes modules for resource exploitation, energy processing & conversion, energy demand sectors (industry, buildings, transport, agriculture). Hourly demand profiles, renewable output characteristics, power regulation performance and multiple flexibility enhancement options (energy storage and demand-side response) are modeled to achieve multi-timescale power supply-demand balance. Technology names with gray background represent CCS have been modelled in these technologies. Technologies names with blue fonts represent hydrogen fuel option have been modelled in these technologies. The framework enables integrated optimization of China’s energy transition across sectors, technologies, and temporal scales to boost a secure and feasible net-zero transition. CAES represents compressed-air energy storage. CCS represents carbon capture and storage, PV represents photovoltaic. CSP represents concentrated solar power. V2G represents vehicle-to-grid technology. P2X represents multiple fuels and chemical products from electricity.

**Fig. 10:**
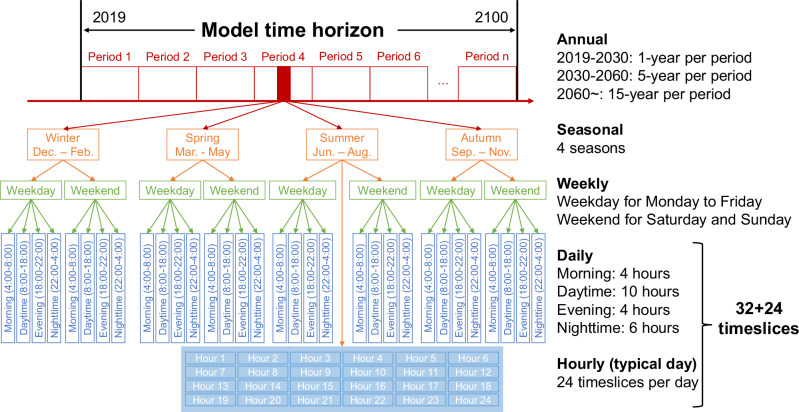
Model horizon and timeslice setting. China TIMES 2.0 features a 2019-2100 model horizon and the flexibility to adjust the length of each period to balance computing workload and accuracy. The model provides a differentiated portrayal of the four seasons, weekdays and weekends, four typical periods of the day, and hourly scheduling of a summer workday.

China TIMES 2.0 is designed as a reconstructed energy-environment-economy model adapted to the deep decarbonization and long-term sustainable transition in China (Fig. 9). With modeling capabilities for water system, land system and air quality system, China TIMES 2.0 is emerging as one of active national-level IAMs for China in the international climate change arena^43^. Building upon the China MARKAL^44–46^ and China TIMES^47–51^ models, which have been widely used in climate change mitigation and energy strategy initiatives, China TIMES 2.0 recalibrates the base year to 2019, with a model horizon spanning the entire century (2019 to 2100). Model outputs for 2020-2023 were calibrated against official statistics. Distinctively, the model achieves flexible time steps, with a one-year period until 2030, a five-year period from 2030 to 2060, and several 15-year periods thereafter. China TIMES 2.0 models the entire energy system at the sub-annual level, constructing 56 timeslices by stratifying the selection of representative time periods according to the annual-seasonal-weekly-daily-hourly nesting (Fig. 10), thus increasing cross-timescale carving capability as much as possible while taking into account computational efficiency. As a bottom-up linear planning framework, China TIMES 2.0 portrays the whole energy system process of energy extraction, energy processing, energy transmission, energy distribution, and energy end-use. The built-in emulator is connected to the GLOBIOM model, thus covering the main greenhouse gas emissions such as CO_2_, CH_4_ and N_2_O from the FFI as well as the agriculture, forestry and other land use (AFOLU) sector^52^.

Vast existing and future technologies empower the model to provide path projections with technical details. The energy demand for heating, cooling, hot water, cooking, refrigerators, electrical appliances, and data centers in the building sector is divided into 15 sub-areas based on five climate zones and three building types (urban, rural, commercial)^53^. The model classifies existing building envelopes from 1980 according to five generations of building standards and adds modeling of ultra-low and near-zero energy building envelopes. Old buildings can reduce their heating and cooling demands through envelope retrofits. From traditional energy-intensive technologies such as direct solid biomass combustion and incandescent lighting to advanced low-carbon technologies such as ground-source heat pumps and natural gas-hydrogen blended combustion, more than 50 energy end-use technologies and nearly 20 new and retrofit options for envelopes provide staunch support for building energy simulation in different building types and regions. Industrial production such as Na_2_CO_3_, NaOH, NH_3_, C_2_H_4_, cement^54,55^, glass, steel^56–59^, paper, and non-ferrous are modeled at the process level. To meet the need for deep decarbonization, fuels such as coal, oil, gas, hydrogen, biomass, and electricity are widely introduced, making up more than 100 industrial energy technologies. Benchmark, baseline and historical values are set for all technical processes to encourage to increase energy efficiency^60^. As for transportation sector, the model covers gasoline, diesel, electricity, hydrogen, natural gas, LPG, biofuel and carves motorcycles, low-speed three or four wheelers, light-duty vehicles, medium-sized buses, large buses, cabs, mini-trucks, small trucks, medium trucks, large trucks, normal trains, high-speed trains, subways, aviation, navigation, and pipelines, which are used to meet the passenger and freight transportation needs in different distances (intracity, intercity, and international)^61^.

The model also designed various energy supply and transmission technologies. Electricity and heat production are highlighted separately (see next section). Technologies such as fossil fuel extraction and trade, hydrogen production from fossil fuels (with or without CCS) and water electrolysis, coal washing, oil refining (with or without CCS), coking (with or without CCS), coal to oil or gas, biomass to oil or gas, water supply (groundwater, surface water, desalination, wastewater treatment), direct air capture storage (DACS) are represented in the upstream sector.

### Electricity and heat production

China TIMES 2.0 model clusters existing power and heating units into 100 representative technologies with vintage based on plant-level data for coal, natural gas and nuclear power, as well as renewable energy data by technology category from China Electricity Council (CEC) and considers 186 future technologies that could be built new or retrofitted on existing units^62,63^. For the determination of the cooling type of each unit, information from the CEC is mainly used and cross-referenced with Google satellite maps on a plant-by-plant basis.

For thermal power, technologies are classified according to fuel type (biomass, coal, gas, oil, biomass-coal blending), unit size (1000 megawatts class, 600 megawatts class, 300 megawatts class, 100 megawatts class, small units), cooling method (air cooling, once-through cooling, recirculating cooling), steam pressure (ultra-supercritical, supercritical, subcritical, high-pressure), and whether combined heat and power (CHP) units^64–66^. The model portrays the ongoing CFPP retrofit plan. Flexibility retrofit, CCS retrofit, biomass-coal blending retrofit, and BECCS retrofit can be performed on compatible units. At the same time, units are able to gain better regulation capacity through additional investment (500 Chinese Yuans kW^-1^)^67^. Taking the ultra-supercritical unit as an example, the minimum load level after the retrofit dips from 40% to 20%. For nuclear energy, the model considers pressurized water reactors and determines a capacity limit based on the availability of coastal locations^68^. The model also portrays new types of nuclear reactors, such as the high-temperature gas-cooled reactor, which have better safety features and are assumed to be built inland, and therefore avoid the location constraint. All thermal power plants and nuclear reactors are set up with parameters such as ramp-up and ramp-down costs, start-stop costs, ramp-up and ramp-down rate constraints, minimum output levels, to more realistically reflect the operation of the power system^20,69^.

Renewable energy is expected to experience extremely rapid growth, because of its nature, it may pose risks of power system security. Run-of-river and regulated hydropower are portrayed separately in the model, and both provide supply curves with varying costs, thus reflecting the value of regulation resources. Offshore wind and onshore wind are treated differently according to the real data in 2018 representing the hour-by-hour output going forward^70^. Similarly, centralized PV, distributed PV, building-integrated PV (BIPV), and centralized solar power (CSP) set future sub-annual operations based on hourly data in 2018^70^. We pre-processed the raw data for 2018 to find the hourly PV and wind generation and load as a percentage of the total for the year. In this way, we extracted the seasonal and intraday variations that characterize renewables and load, and excluded the influence of trend changes on the results. In addition, wave energy, tidal energy, geothermal energy, are also modeled. Biomass-fired power is one of the alternative choices to mitigate high-emitted CFPPs and can be equipped with CCS technology to contribute valuable negative emissions. There are fuel substitution technologies for almost every size of CFPPs, and a variety of combustion types such as biomass combustion, biomass gasification, and biomass-coal blending, as well as options for units equipped with CCS. In the model, we introduce the constraint of the peak load reserve margin, in which we require that the total credible capacity exceeds 10% of the peak load at each timepoint. The contribution of each technology to the peak load is described in Supplementary Table 1. The constraints on the capacity of the power plant are described in Supplementary Table 2.

### Future load profile generation

As China is still in its rapid electrification progress, electricity is meeting energy service demands in expanding areas. As a result, future electricity load curves are expected to change significantly due to the addition of new electrical appliances. In this study, we propose a method to determine the time distribution of energy use according to behavioral patterns and construct energy use profiles for different energy service demands, thus avoiding exogenous prediction of future load curve. In this paper, according to the mandatory national standard issued in 2021, energy use for lighting, hot water, and cooking in different rooms is split by time of a day. The space cooling and heating demand is differentiated for different seasons, and they are also modeled by time of a day^71^. For electricity use for transportation, this study mainly considers the timing and mode of EV charging. In view of the various categories of EV chargers, fast charging and slow charging are portrayed according to the New Energy Vehicle Industry Development Plan (2021-2035), which corresponds to different time distribution of energy use in a day (Supplementary Table 3). Data center operations often require consistency, and their energy consumption cannot vary dramatically in a day. Electricity consumption from industry is used as the residue in the load profile to match historical load curves and thus complete the calibration for each sector. For the specific energy use curves for each demand, refer to Supplementary Fig. 14.

### Energy storage and sectoral coupling

Given the unregulated nature of VRE, it is physically hard to achieve a real-time supply-demand balance in a power system with a high share of VRE, thus requiring energy storage. China TIMES 2.0 portrays electrical energy storage technologies such as pumped hydro, CAES, liquid flow batteries (VRB and Zn-Br), lithium-ion batteries (NMC, NCA, LFP, LTO), lead-acid batteries, molten salt batteries (Na-S and zebra), superconducting magnetic energy storage, and flywheel energy storage^72^. China TIMES 2.0 considers the current policy and sets a lower limit for energy storage development (10% of VRE capacity) and sets a lower limit for future pumped hydro capacity in accordance with the Medium- and Long-Term Development Plan for Pumped Hydro (2021-2035). In addition to the energy storage, China TIMES 2.0 also includes technological options for sectoral coupling. For non-ferrous metals smelting, EAF steelmaking processes, seawater desalination, and water electrolysis for hydrogen production, these technologies can be regulated more easily and flexibly, thus allowing them to be worked in an orderly manner within a certain range, bridging the gap between power supply and demand in real time. Furthermore, liquefied hydrogen storage and hydrogen-natural gas blending are also portrayed, thus maximizing the exploitation of flexibility resources.

### Active demand-side response measures

In a market environment, where demand responds to both price changes and incentive signals, demand-side response measures are considered to have enormous potential to help balance the grid. All energy service demands can respond to price changes at each timeslice (annual, seasonal, weekly, daily, hourly) by means of price elasticity of demand which helps us model the curtailable load. Over a longer time scale, the price elasticity of demand can act to cut demand due to price increases resulting from the deployment of abatement technologies. Thus, it can represent the consumer behavior shift.

For all energy service demands, the energy demand can be time-shifted through incentives, thus reducing mismatch between supply and demand. EV charging demand can not only be shifted but can be reversed to supply power to the grid through V2G technology. Based on the TIMES open-source framework^73,74^, the China TIMES 2.0 model models load shifting as a special bi-directional energy storage technology that is able to represent the maximum allowed deviations from nominal demand loads, maximum advance or delay for meeting the shifted loads, and the value per hour shifted by setting the storage capacity, the storage time, and the operating cost, respectively. The model conservatively assumes a subsidy of approximately 4 US cents kWh^-1^ hour^-1^ of shifting, whether advanced or delayed, based on a subsidy of 1 Chinese Yuan kWh^-1^ for three-hour load shifting in Yunnan Province.

### Scenario setting

In this study, four hourly scenarios are proposed. The NDC, as a reference, contains energy and climate policies issued until 2020 and considers the climate target of carbon peaking by 2030 in the updated NDCs. Carbon intensity reduction of more than 65% from 2005 and VRE capacity exceeding 1200 GW targets are bounded in the NDC scenario. The scenario is the cost-optimal solution within physical and technical feasibility constraints. The CN60, CN60-noLM and CN60-LM scenarios all address the carbon neutrality commitment of China (FFI CO_2_ net-zero by 2060) and correspond to distinct levels of demand-side participations. In addition, we propose a scenario CN60-noTS without sub-annual constraints, which removes the seasonal, weekly, and daily variability carve-outs of renewable energy outputs and loads (assumed to be averaged distributions) and relaxes the power unit flexibility constraints. CN60-DN represents the fluctuations in renewable energy and load considering only daytime and nighttime, which is the practice of the China TIMES 1.0 model^48^. CN60-noHour scenario depicts four seasons, weekdays and weekends, and four time periods in a day, for a total of 32 timeslices, similar to the leading national TIMES model.

For all net-zero scenarios, we set the cumulative carbon emissions for 2019-2100 under RCP2.6 assumption based on the carbon budget allocation principle (230 billion tons of CO_2_). The global carbon budget allocation is calculated according to the Equal Cumulative Per Capita (ECPC) methodology, with a historical allocation starting point in 1990, and a global pathway that selects the median pathway of the IPCC Sixth Assessment Report Category C4 (limit warming in 2 degrees with >50% probability). It also assumes that China follows the path of the NDC scenario until 2030, to reflect the inertia of the energy system and the institutional constraints. Price elasticity of demand and early retirement of power plant and industrial capacity are also allowed in all net-zero scenarios. For NDC and all net-zero scenarios, we set capacity limits for coal, nuclear, wind, and PV power plants based on policy and resource potential. For all end-use sectors, we assumed no further increase in the share of fossil fuel. In addition, for all scenarios, we require total carbon emissions to decline monotonically after peaking (no rebound).

The difference in assumptions between main scenarios centers on the difference in the degree of demand-side contribution to system flexibility. CN60-noLM represents a scenario where no demand-side response measures are implemented, and the energy user consumes energy in the same way as they do presently. This represents the case where energy prices do not affect demand-side behavior at all. CN60 is an intermediate scenario that envisions the electricity demand for transportation and hydrogen production adapting to variations in online electricity generation capacity. In this scenario, energy prices have an impact on demand that can be easily regulated, such as hydrogen production and EV charging, and users regulate the time distribution of energy use in response to price signals. Meanwhile, CN60-LM is a supply-demand interaction scenario that introduces load time-shifting across a wide range of industries alongside V2G technology and home energy storage feed-in. In this scenario, most of the energy service demands are capable of demand-side response with price incentives, and in particular, EV batteries, and home energy storage can be dispatched to balance system supply and demand.

For comparison, we introduced six prominent global IAMs. China TIMES and these six IAMs worked together on a simulation of the Glasgow+ scenario, guided by the ENGAGE project proposal. This task aims at exploring the consistency of mid-century strategies and policies with the overall objectives of the Paris Agreement. The protocol integrates national and global pathways into a coherent set of low-carbon mid-century strategies (Supplementary Table 4), assessing national mid-century strategies and their consistency with global pathways to 1.5 and 2 °C warming levels and identifying the most effective policies in different countries and sectors. The proposal does not mandate harmonization of input assumptions across model groups due to differences in calibration data sources for national and global models. Based on the model input information voluntarily reported by the teams, China TIMES model is in the middle of the models in terms of the demand assumptions for steel, cement, and residential and commercial floor space. The GDP assumptions are on the high side of the models, and the population assumptions are on the low side of the models. Despite the differences in demand assumptions, for the trajectory trends and quantity differences in power sector emissions, storage, and thermal power identified by the comparisons in this paper, they can be accounted for by the implications of sub-annual level modeling.

## Supplementary information

Supplementary Information

Transparent Peer Review file

## Source data

Source Data

## Data Availability

All annual scale IAM scenarios are made accessible online via the ENGAGE Scenario Portal at https://data.ece.iiasa.ac.at/engage. Main parameter assumptions are listed in the Supplementary Table 5. Other data that indirectly supports the findings of this study and all code for result visualization are available from the corresponding author upon request. Source data are provided with this paper.

## Code availability

The code of the TIMES model is open source and available on GitHub (https://github.com/etsap-TIMES/TIMES_model), and this study is run using TIMES 4.7.3. The documentation of the TIMES model is available on website (https://iea-etsap.org/index.php/documentation). For the result analysis and visualization tasks, we mainly used Python 3.11 and pyam-iamc 2.2.4 package, which are freely available. The model has about 200 million non-zero values, and applying the Barrier method of the Gurobi 11 software to solve it requires about 10 hours of computation and about 100 GB of memory on a workstation with 64 CPU cores.
