# Supplementary material for: Exploring the feasible net-zero transition pathway in China considering energy system flexibility
Source: Nat Commun. 2026 Apr 11;17:5440. doi: 10.1038/s41467-026-71410-2 (PMC13279959; doi:10.1038/s41467-026-71410-2)
Supplement: Supplementary file 1 — Supplementary Information [file 41467_2026_71410_MOESM1_ESM.pdf]

Supplementary Information

Exploring the feasible net-zero transition pathway in China considering energy system flexibility

Shu Zhang (张枢)[1,2], Wenying Chen (陈文颖)[1,2,*]

1 Research Center for Contemporary Management, Tsinghua University, 100084 Beijing, China
2 Institute of Energy, Environment and Economy, Tsinghua University, 100084 Beijing, China
* Corresponding author: Wenying Chen (chenwy@tsinghua.edu.cn)

Contents of the Supplementary Items

Supplementary Table 1 Fraction of capacity that can contribute to the peak load 3
Supplementary Table 2 Power capacity limit assumptions 4
Supplementary Table 3 The percentage of energy consumption at different hours of the day in the building and transportation sectors 5
Supplementary Table 4 Net-zero CO_2 target year based on official submissions, aggregated to the GCAM regions, for Glasgow+ scenario 6
Supplementary Table 5 Power plant construction, energy storage construction, hydrogen electrolyzer construction and demand side response costs 8
Supplementary Fig. 1 Low-carbon energy development in the industrial sector 9
Supplementary Fig. 2 Low-carbon energy development for space heating in 2060 10
Supplementary Fig. 3 Low-carbon energy development for cooking 11
Supplementary Fig. 4 Low-carbon energy development for light-duty vehicles 12
Supplementary Fig. 5 Low-carbon energy development for trucks....................................... 13
Supplementary Fig. 6 The load and net load variation of the typical summer day in China. 14
Supplementary Fig. 7 Electricity consumption in 2060 by industry for different seasons of the year and different working days 15
Supplementary Fig. 8 Electricity generation in 2060 by technology for different seasons of the year and different working days 16
Supplementary Fig. 9 Energy storage facility operation mode in 2060 by technology for different seasons of the year and different working days 17
Supplementary Fig. 10 Electricity consumption in the transportation sector in 2060 by technology for different seasons of the year and different working days 18
Supplementary Fig. 11 Hydrogen consumption by sector in 2060 for different seasons of the year and different working days 19
Supplementary Fig. 12 Hydrogen production by technology in 2060 for different seasons of the year and different working days 20
Supplementary Fig. 13 Energy transition cost in electricity, hydrogen and storage for China during 2020-2100 21
Supplementary Fig. 14 Behavior-based load curve generation procedure 22
Supplementary Note 1 China's energy transition towards carbon neutrality 23
Supplementary Fig. 15 China's energy system decarbonization pathway for all scenarios . 23
Supplementary Note 2 Sensitivity scenario analysis for flexibility retrofits............................ 24
Supplementary Fig. 16 Differences in electricity capacity by technology between the flexibility retrofit sensitivity scenario and the core scenario 25
Supplementary Fig. 17 Differences in electricity supply by technology for the typical day in 2060 between the flexibility retrofit sensitivity scenario and the core scenario 26
Supplementary Note 3 Sensitivity scenario analysis for vehicle-to-grid application rates .. 27
Supplementary Fig. 18 Differences in electricity supply by technology for the typical day in 2060 between the vehicle-to-grid application rate sensitivity scenario and the core scenario 28
Supplementary Fig. 19 Vehicle-to-grid operations in 2060 under different scenarios 29

Supplementary Table 1 Fraction of capacity that can contribute to the peak load

Each power system technology is assigned a coefficient from 0 to 1 to represent the proportion of credible output capacity to total capacity at every timepoint. This can be used to reflect the volatility of different power sources.

Technology	Fraction
Combined heat and power plant	0.95
Thermal power plant	0.97
Nuclear power	0.97
Geothermal	0.95
Hydropower	0.50
Ocean	0.15
Solar – Concentrated solar power	1.00
Solar – Photovoltaic	0.15
Wind	0.15
Storage	1.00

Supplementary Table 2 Power capacity limit assumptions

The coal power capacity upper limit is set based on existing coal power construction projects from Global Energy Monitor database, assuming that no new coal power units will be built after 2030 according to the policy requirement; it is assumed that only coastal nuclear power construction will be permitted before 2030, and inland nuclear power (only Generation IV reactors) will be allowed to be built according to the 14th Five-year Development Plan; The upper limit for wind and solar capacity is based on the National Energy Administration(NEA)'s guidance of 200 gigawatts (GW) of PV and 80 GW of wind per year in the short term, and on the results of the wind resource assessment in the long term. We set the lower limit of pumped hydro at 60 GW and 120 GW in 2025 and 2030 according to the Pumped Hydro Plan issued by NEA, and according to the long-term planning layout and potential assessment reported by NEA, we set the upper limit at 420 GW in 2060, and the upper limit at 500 GW in 2100.

Technology	Unit	2025	2030	2060	2100
Coal power plant capacity upper limit	GW	1300	1350	1350	1350
Nuclear power capacity upper limit	GW	200	200	350	400
Hydropower capacity upper limit	GW	445	500	615	700
Wind power capacity lower limit	GW	600	1000	/	/
Wind power capacity upper limit	GW	/	/	3500	5500
Solar power capacity lower limit	GW	1000	2000	/	/
Solar power capacity upper limit	GW	/	/	6800	8000
Pumped hydro capacity lower limit	GW	60	120	/	/
Pumped hydro capacity upper limit	GW	/	/	420	500

Supplementary Table 3 The percentage of energy consumption at different hours of the day in the building and transportation sectors

The percentage of energy use by time in a day in the building sector refers to the national standard GB 55015-2021 “General specifications for building energy efficiency and renewable energy use”. The percentage of energy use by time in a day in the transportation sector refers to the “New Energy Vehicle Industry Development Plan (2021-2035)”. The data is processed by spline smoothing.

Demand	H1	H2	H3	H4	H5	H6	H7	H8	H9	H10	H11	H12	H13	H14	H15	H16	H17	H18	H19	H20	H21	H22	H23	H24
**Commercial**	**2.4%**	**2.4%**	**2.4%**	**2.4%**	**2.4%**	**2.6%**	**3.1%**	**3.9%**	**5.1%**	**5.7%**	**6.0%**	**5.5%**	**5.4%**	**5.8%**	**5.7%**	**5.7%**	**5.9%**	**5.7%**	**4.9%**	**4.6%**	**4.3%**	**3.2%**	**2.8%**	**2.5%**
Cooling	0.8%	0.8%	0.8%	0.8%	0.8%	0.8%	0.8%	0.8%	6.0%	6.0%	6.0%	6.0%	6.8%	6.8%	6.8%	6.8%	6.8%	6.8%	6.8%	6.8%	6.8%	3.0%	3.0%	1.7%
Cooking	2.1%	2.1%	2.1%	2.1%	2.1%	4.8%	6.5%	4.8%	3.4%	4.8%	8.3%	9.2%	6.5%	4.8%	2.5%	3.0%	6.1%	8.3%	4.8%	3.0%	2.5%	2.1%	2.1%	2.1%
Heating	3.3%	3.3%	3.3%	3.3%	3.3%	3.3%	5.3%	5.3%	4.5%	4.5%	4.5%	4.5%	4.5%	4.5%	4.5%	4.5%	4.5%	4.5%	4.5%	5.3%	5.3%	3.3%	3.3%	3.3%
Lighting	0.6%	0.6%	0.6%	0.6%	0.6%	0.6%	1.4%	4.8%	7.5%	7.5%	7.5%	6.7%	6.7%	7.5%	7.7%	7.7%	8.4%	5.6%	6.0%	4.4%	4.4%	1.4%	0.6%	0.6%
Data center	4.2%	4.2%	4.2%	4.2%	4.2%	4.2%	4.2%	4.2%	4.2%	4.2%	4.2%	4.2%	4.2%	4.2%	4.2%	4.2%	4.2%	4.2%	4.2%	4.2%	4.2%	4.2%	4.2%	4.2%
Others	1.8%	1.8%	1.8%	1.8%	1.8%	1.8%	2.0%	3.8%	5.5%	6.9%	7.0%	5.4%	5.7%	6.8%	6.9%	6.9%	6.5%	6.3%	4.6%	4.3%	3.6%	3.3%	2.2%	1.8%
Hot water	3.6%	3.5%	3.5%	3.5%	3.6%	3.7%	3.8%	3.9%	4.0%	4.1%	4.3%	4.4%	4.6%	4.7%	4.8%	4.9%	4.9%	4.8%	4.7%	4.6%	4.5%	4.2%	3.9%	3.7%
**Rural**	**3.0%**	**3.0%**	**3.0%**	**3.0%**	**3.2%**	**4.6%**	**5.0%**	**4.0%**	**3.5%**	**3.7%**	**4.4%**	**4.4%**	**4.1%**	**4.2%**	**4.1%**	**4.2%**	**4.9%**	**5.6%**	**5.5%**	**5.8%**	**5.5%**	**4.3%**	**3.8%**	**3.3%**
Heating	2.5%	2.5%	2.5%	2.5%	2.5%	2.5%	2.5%	2.5%	3.9%	3.9%	3.9%	3.9%	3.9%	6.8%	6.8%	6.8%	6.8%	6.8%	6.8%	6.8%	3.9%	3.9%	2.5%	2.5%
Cooling	2.1%	2.1%	2.1%	2.1%	2.1%	4.8%	6.5%	4.8%	2.5%	3.9%	9.2%	7.4%	4.8%	3.4%	2.5%	3.0%	7.4%	10.1%	7.9%	2.5%	2.5%	2.1%	2.1%	2.1%
Hot water	4.9%	4.9%	4.9%	4.9%	4.9%	4.9%	3.3%	3.3%	3.3%	3.3%	3.3%	3.3%	3.3%	3.3%	3.3%	3.3%	3.3%	4.9%	4.9%	4.9%	4.9%	4.9%	4.9%	4.9%
Refrigeration	4.2%	4.2%	4.2%	4.2%	4.2%	4.2%	4.2%	4.2%	4.2%	4.2%	4.2%	4.2%	4.2%	4.2%	4.2%	4.2%	4.2%	4.2%	4.2%	4.2%	4.2%	4.2%	4.2%	4.2%
Lighting	0.7%	0.7%	0.7%	0.7%	1.7%	9.9%	10.4%	3.4%	1.9%	1.4%	1.4%	1.4%	1.4%	1.4%	1.4%	1.4%	2.6%	4.4%	6.6%	14.9%	15.4%	7.7%	5.7%	2.7%
Cooking	2.7%	2.7%	2.9%	3.1%	3.5%	4.0%	5.7%	5.5%	3.5%	3.7%	3.5%	5.7%	5.3%	3.9%	3.6%	3.6%	4.3%	6.1%	5.9%	5.6%	4.8%	4.2%	3.5%	2.9%
Appliances	3.6%	3.5%	3.5%	3.5%	3.6%	3.7%	3.8%	3.9%	4.0%	4.1%	4.3%	4.4%	4.6%	4.7%	4.8%	4.9%	4.9%	4.8%	4.7%	4.6%	4.5%	4.2%	3.9%	3.7%
**Urban**	**3.0%**	**3.0%**	**3.0%**	**3.0%**	**3.2%**	**4.5%**	**4.8%**	**3.9%**	**3.5%**	**3.7%**	**4.3%**	**4.3%**	**4.1%**	**4.3%**	**4.2%**	**4.2%**	**4.9%**	**5.6%**	**5.5%**	**5.9%**	**5.5%**	**4.4%**	**3.8%**	**3.3%**
Heating	2.5%	2.5%	2.5%	2.5%	2.5%	2.5%	2.5%	2.5%	3.9%	3.9%	3.9%	3.9%	3.9%	6.8%	6.8%	6.8%	6.8%	6.8%	6.8%	6.8%	3.9%	3.9%	2.5%	2.5%
Cooling	2.1%	2.1%	2.1%	2.1%	2.1%	4.8%	6.5%	4.8%	2.5%	3.9%	9.2%	7.4%	4.8%	3.4%	2.5%	3.0%	7.4%	10.1%	7.9%	2.5%	2.5%	2.1%	2.1%	2.1%
Hot water	4.9%	4.9%	4.9%	4.9%	4.9%	4.9%	3.3%	3.3%	3.3%	3.3%	3.3%	3.3%	3.3%	3.3%	3.3%	3.3%	3.3%	4.9%	4.9%	4.9%	4.9%	4.9%	4.9%	4.9%
Refrigeration	4.2%	4.2%	4.2%	4.2%	4.2%	4.2%	4.2%	4.2%	4.2%	4.2%	4.2%	4.2%	4.2%	4.2%	4.2%	4.2%	4.2%	4.2%	4.2%	4.2%	4.2%	4.2%	4.2%	4.2%
Lighting	0.7%	0.7%	0.7%	0.7%	1.7%	9.9%	10.4%	3.4%	1.9%	1.4%	1.4%	1.4%	1.4%	1.4%	1.4%	1.4%	2.6%	4.4%	6.6%	14.9%	15.4%	7.7%	5.7%	2.7%
Cooking	2.7%	2.7%	2.9%	3.1%	3.5%	4.0%	5.7%	5.5%	3.5%	3.7%	3.5%	5.7%	5.3%	3.9%	3.6%	3.6%	4.3%	6.1%	5.9%	5.6%	4.8%	4.2%	3.5%	2.9%
Appliances	3.6%	3.5%	3.5%	3.5%	3.6%	3.7%	3.8%	3.9%	4.0%	4.1%	4.3%	4.4%	4.6%	4.7%	4.8%	4.9%	4.9%	4.8%	4.7%	4.6%	4.5%	4.2%	3.9%	3.7%
**Transport**	**6.9%**	**7.4%**	**7.0%**	**6.2%**	**5.2%**	**4.2%**	**3.4%**	**2.8%**	**2.4%**	**2.0%**	**1.8%**	**1.7%**	**1.7%**	**1.7%**	**1.7%**	**1.9%**	**2.3%**	**2.9%**	**3.6%**	**4.6%**	**5.8%**	**6.9%**	**7.8%**	**8.0%**
Fast	1.7%	5.1%	4.7%	2.8%	1.1%	0.3%	0.7%	2.0%	3.9%	6.0%	7.7%	8.9%	9.1%	8.5%	7.1%	5.3%	3.4%	1.9%	1.3%	1.6%	3.0%	4.7%	5.6%	3.6%
Slow	7.8%	7.8%	7.5%	6.8%	5.9%	4.9%	3.9%	3.0%	2.1%	1.4%	0.8%	0.5%	0.3%	0.5%	0.8%	1.4%	2.1%	3.0%	4.1%	5.1%	6.2%	7.3%	8.1%	8.7%

Supplementary Table 4 Net-zero CO_2 target year based on official submissions, aggregated to the GCAM regions, for Glasgow+ scenario

Region	Countries	Net-zero year
Eastern Africa	Burundi, Comoros, Djibouti, Eritrea, Ethiopia, Kenya, Madagascar, Mauritius, Reunion, Rwanda, Sudan, Somalia, Uganda	2070
Northern Africa	Algeria, Egypt, Western Sahara, Libya, Morocco, Tunisia	2045
Southern Africa	Angola, Botswana, Lesotho, Mozambique, Malawi, Namibia, Swaziland, Tanzania, Zambia, Zimbabwe	2070
Western Africa	Benin, Burkina Faso, Central African Republic, Cote d'Ivoire, Cameroon, Democratic Republic of the Congo, Congo, Cabo Verde, Gabon, Ghana, Guinea, Gambia, Guinea-Bissau, Equatorial Guinea, Liberia, Mali, Mauritania, Niger, Nigeria, Senegal, Sierra Leone, São Tomé and Príncipe, Chad, Togo	2060
Argentina	Argentina	2040
Australia/New Zeeland	Australia, New Zeeland	2040
Brazil	Brazil	2040
Canada	Canada	2040
Central America and the Caribbean	Aruba, Anguilla, Netherlands Antilles, Antigua & Barbuda, Bahamas, Belize, Bermuda, Barbados, Costa Rica, Cuba, Cayman Islands, Dominica, Dominican Republic, Guadeloupe, Grenada, Guatemala, Honduras, Haiti, Jamaica, Saint Kitts and Nevis, Saint Lucia, Montserrat, Martinique, Nicaragua, Panama, El Salvador, Trinidad and Tobago, Saint Vincent and the Grenadines	2045
Central Asia	Armenia, Azerbaijan, Georgia, Kazakhstan, Kyrgyzstan, Mongolia, Tajikistan, Turkmenistan, Uzbekistan	2045
China	China	2050
Colombia	Colombia	2040
EU-12	Bulgaria, Cyprus, Czech Republic, Estonia, Hungary, Lithuania, Latvia, Malta, Poland, Romania, Slovakia, Slovenia	2040
EU-15	Andorra, Austria, Belgium, Denmark, Finland, France, Germany, Greece, Greenland, Ireland, Italy, Luxembourg, Monaco, Netherlands, Portugal, Sweden, Spain, United Kingdom	2040
Eastern Europe	Belarus, Moldova, Ukraine	2050

European Free Trade Association	Iceland, Norway, Switzerland	2040
Europe non-EU	Albania, Bosnia and Herzegovina, Croatia, Macedonia, Montenegro, Serbia, Türkiye	2053
India	India	2060
Indonesia	Indonesia	2050
Japan	Japan	2040
Mexico	Mexico	2040
Middle East	United Arab Emirates, Bahrain, Iran, Iraq, Israel, Jordan, Kuwait, Lebanon, Oman, Palestine, Qatar, Saudi Arabia, Syria, Yemen	2040
Pakistan	Pakistan	2070
Russia	Russia	2060
South Africa	South Africa	2050
Northern South America	French Guiana, Guyana, Suriname, Venezuela	2050
Southern South America	Bolivia, Chile, Ecuador, Peru, Paraguay, Uruguay	2040
South Asia	Afghanistan, Bangladesh, Bhutan, Sri Lanka, Maldives, Nepal	2065
Southeast Asia	American Samoa, Brunei Darussalam, Cocos (Keeling) Islands, Cook Islands, Christmas Island, Fiji, Federated States of Micronesia, Guam, Cambodia, Kiribati, Lao Peoples Democratic Republic, Marshall Islands, Myanmar, Northern Mariana Islands, Malaysia, Mayotte, New Caledonia, Norfolk Island, Niue, Nauru, Pacific Islands Trust Territory, Pitcairn Islands, Philippines, Palau, Papua New Guinea, Democratic People’s Republic of Korea, French Polynesia, Singapore, Solomon Islands, Seychelles, Thailand, Tokelau, Timor Leste, Tonga, Tuvalu, Viet Nam, Vanuatu, Samoa	2050
South Korea	South Korea	2050
Taiwan	Taiwan	2040
USA	United States of America	2050

Supplementary Table 5 Power plant construction, energy storage construction, hydrogen electrolyzer construction and demand side response costs

CCS represents carbon capture and storage. PWR represents pressurized water reactor. HYGR represents high-temperature gas-cooled reactor. PV represents photovoltaic. CSP represents concentrated solar power. LFP represents lithium iron phosphate battery. NMC represents nickel manganese cobalt battery. PEM represents proton exchange membrane electrolysis. SOEC represents solid oxide electrolyzer cell electrolysis. V2G represents vehicle-to-grid technology. USD represents US dollar. USD kWh^{-1} $hour^{-1}$ represents US dollar incentive amount for one hour and one kilowatt-hour load shifting operation.

Technology	Unit	2025	2030	2060	2100
**Power Plant**					
Coal without CCS	USD kW^{-1}	547	545	535	535
Coal with CCS	USD kW^{-1}	1031	1000	913	913
Gas without CCS	USD kW^{-1}	550	550	550	550
Gas with CCS	USD kW^{-1}	904	897	897	897
Nuclear (PWR)	USD kW^{-1}	1946	1897	1805	1805
Nuclear (HTGR)	USD kW^{-1}	2571	2286	1829	1805
Biomass without CCS	USD kW^{-1}	1220	1102	735	735
Biomass with CCS	USD kW^{-1}	1916	1726	1136	1136
Hydro	USD kW^{-1}	1066	1066	1066	1066
Onshore Wind	USD kW^{-1}	602	542	439	439
Offshore Wind	USD kW^{-1}	1287	1030	667	667
Solar PV	USD kW^{-1}	377	339	247	247
Solar CSP	USD kW^{-1}	2531	2405	1782	1371
**Energy Storage**					
Pumped Hydro	USD kW^{-1}	908	896	833	754
Battery storage LFP	USD kWh^{-1}	119	110	69	37
Battery storage NMC	USD kWh^{-1}	126	117	73	39
Hydrogen alkaline	USD kW^{-1}	246	232	205	205
Hydrogen PEM	USD kW^{-1}	1043	580	213	213
Hydrogen SOEC	USD kW^{-1}	1712	1384	387	387
**Demand-Side Response**					
Load time-shifting	USD kWh^{-1} $hour^{-1}$	0.04	0.03	0.02	0.02
V2G feed-in cost	USD kWh^{-1}	0.08	0.06	0.04	0.04

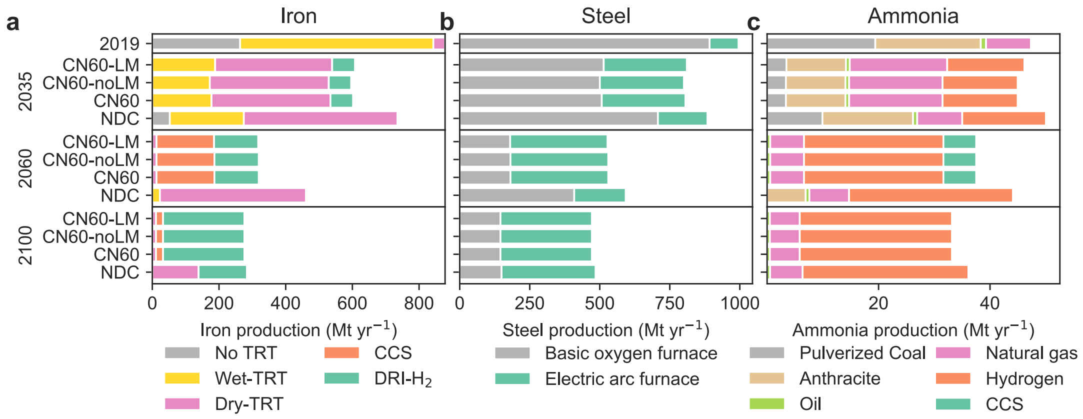

Supplementary Fig. 1 Low-carbon energy development in the industrial sector

a Iron production by technology, **b** steel production by technology, **c** ammonia production by technology. TRT represents top pressure recovery turbine technology. CCS represents carbon capture and storage. DRI-H_2 represents hydrogen direct reduction iron. Mt represents million tons of the product.

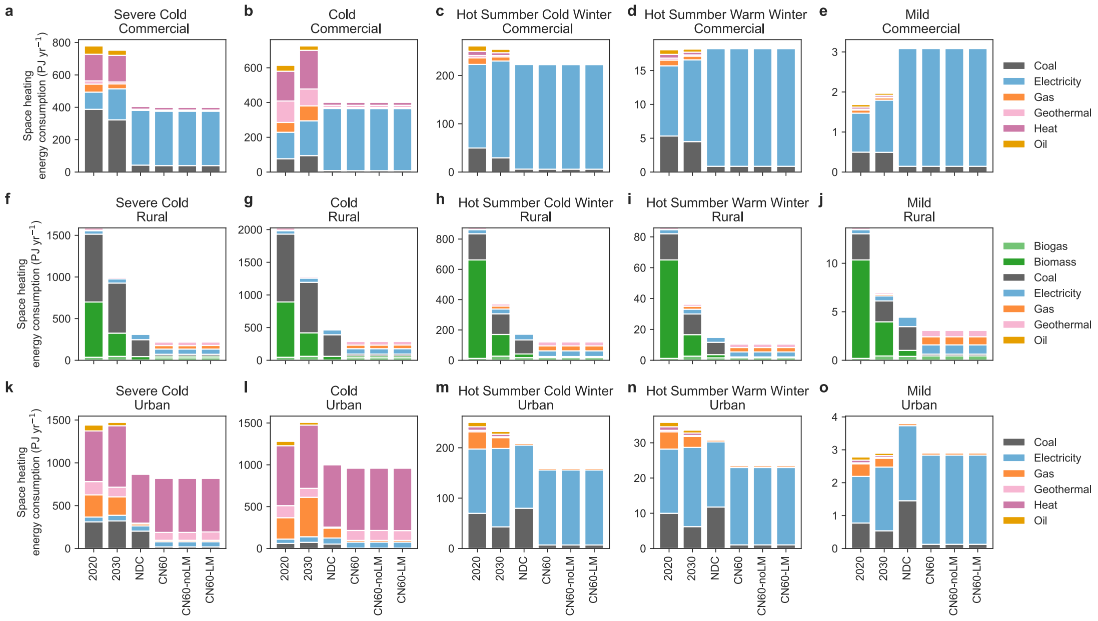

Supplementary Fig. 2 Low-carbon energy development for space heating in 2060

a-e Energy consumption for space heating in commercial buildings in regions classified as severe cold, cold, hot summer cold winter, hot summer warm winter, and mild by fuel, respectively. **f-j** Energy consumption for space heating in rural residential buildings in regions classified as severe cold, cold, hot summer cold winter, hot summer warm winter, and mild by fuel, respectively. **k-o** Energy consumption for space heating in urban residential buildings in regions classified as severe cold, cold, hot summer cold winter, hot summer warm winter, and mild by fuel, respectively. PJ represents petajoule.

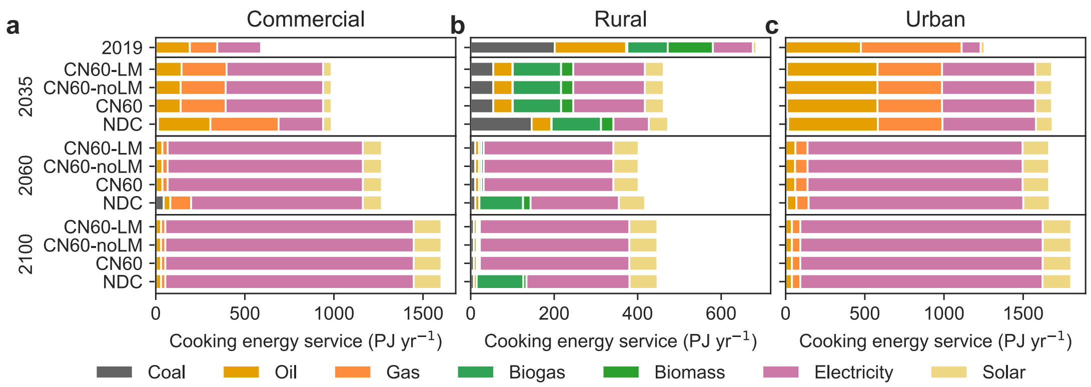

Supplementary Fig. 3 Low-carbon energy development for cooking

a Cooking energy service in commercial buildings by fuel. **b** Cooking energy service in rural residential buildings by fuel. **c** Cooking energy service in urban residential buildings by fuel. PJ represents petajoule.

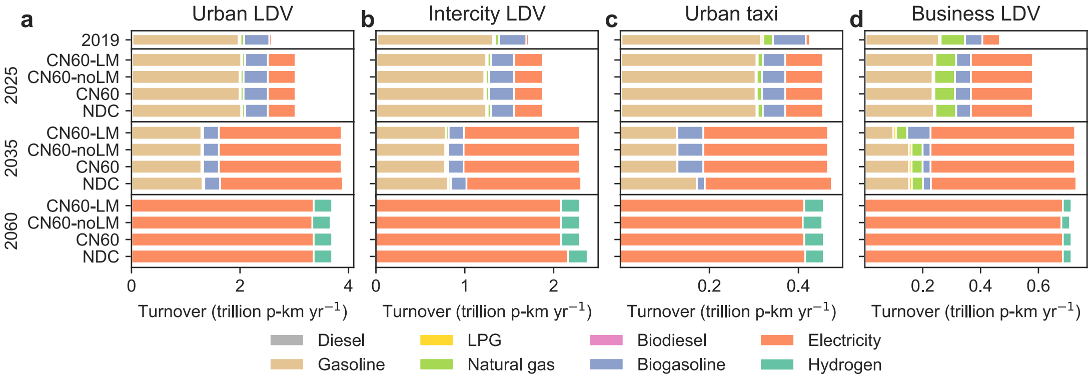

Supplementary Fig. 4 Low-carbon energy development for light-duty vehicles

a Turnover of light-duty vehicles (LDV) in urban areas by fuel. **b** Turnover of LDVs in intercity areas by fuel. **c** Turnover of LDVs used as taxi by fuel. **d** Turnover of LDVs used as business vehicles by fuel. p-km represents person-kilometer.

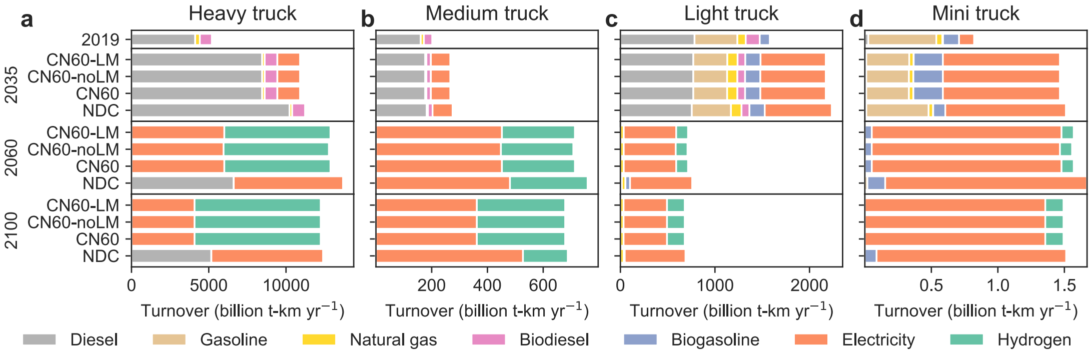

Supplementary Fig. 5 Low-carbon energy development for trucks

a Turnover of heavy trucks by fuel. **b** Turnover of medium trucks by fuel. **c** Turnover of light trucks by fuel. **d** Turnover of mini trucks by fuel. t-km represents ton-kilometer.

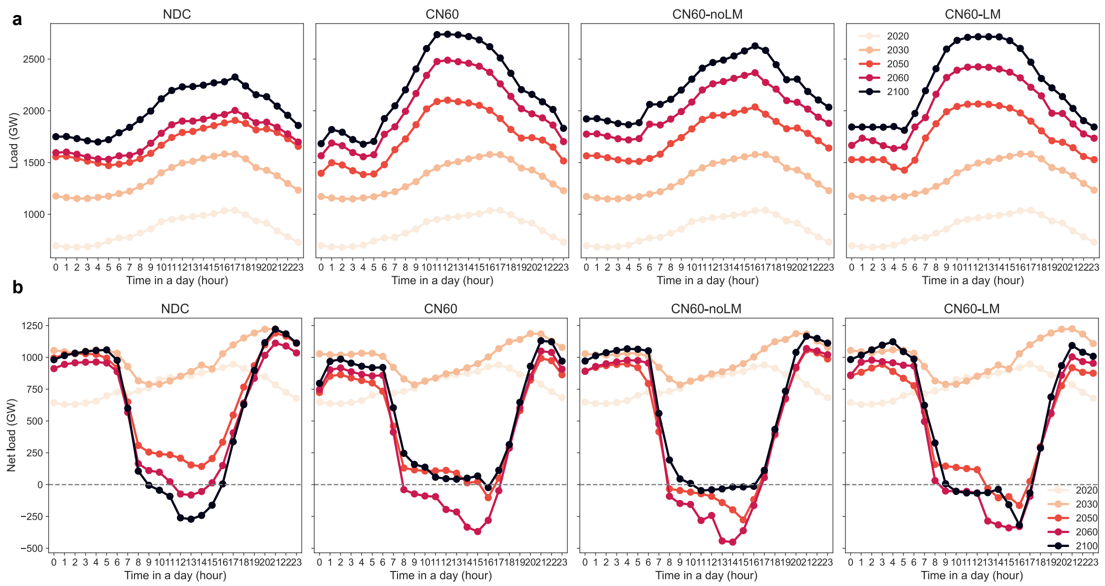

Supplementary Fig. 6 The load and net load variation of the typical summer day in China
a The real electricity load (except storage charging), **b** The net load. The net load is the difference between the real electricity load (except storage charging) and the output of variable renewables (wind and photovoltaic power generation). GW represents gigawatt.

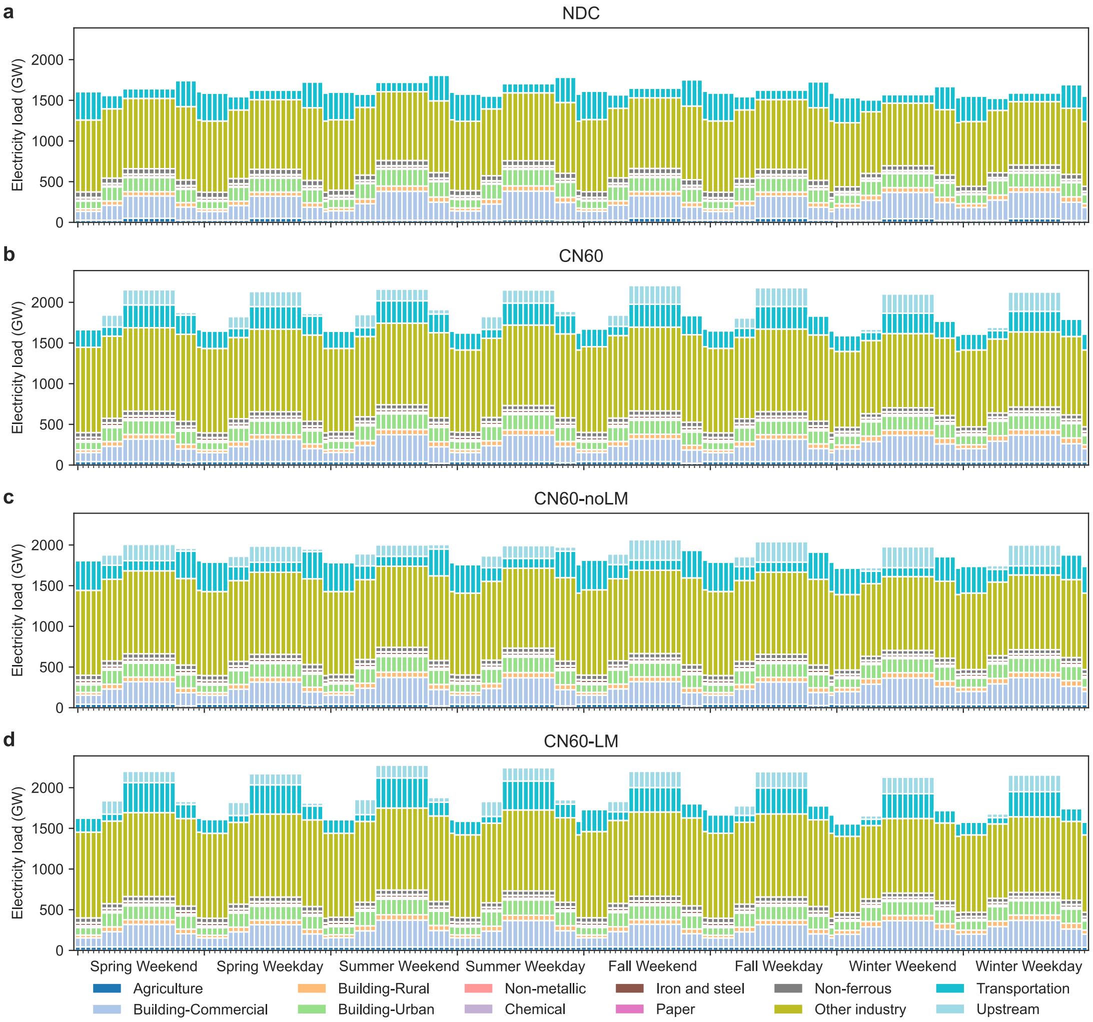

Supplementary Fig. 7 Electricity load in 2060 by industry for different seasons of the year and different working days

a Electricity load by industry under the NDC scenario. **b** Electricity load by industry under the CN60 scenario. **c** Electricity load by industry under the CN60-noLM scenario. **d** Electricity load by industry under the CN60-LM scenario. GW represents gigawatt.

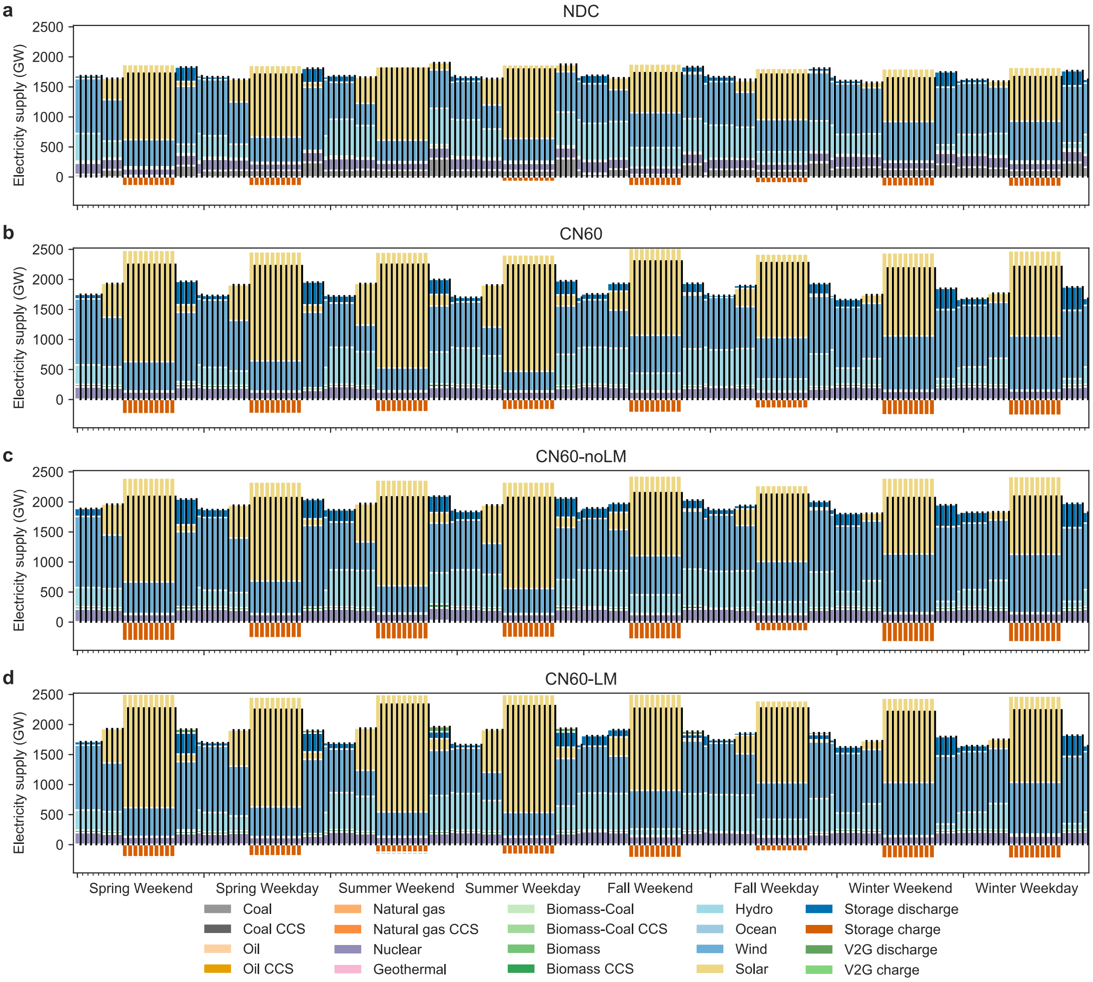

Supplementary Fig. 8 Electricity generation in 2060 by technology for different seasons of the year and different working days

a Electricity generation by technology under the NDC scenario. **b** Electricity generation by technology under the CN60 scenario. **c** Electricity generation by technology under the CN60-noLM scenario. **d** Electricity generation by technology under the CN60-LM scenario. In the legend, the fuel names ending in CCS indicate power plants equipped with carbon capture and storage (CCS) and otherwise indicate power plants without CCS. GW represents gigawatts. V2G represents vehicle-to-grid technology.

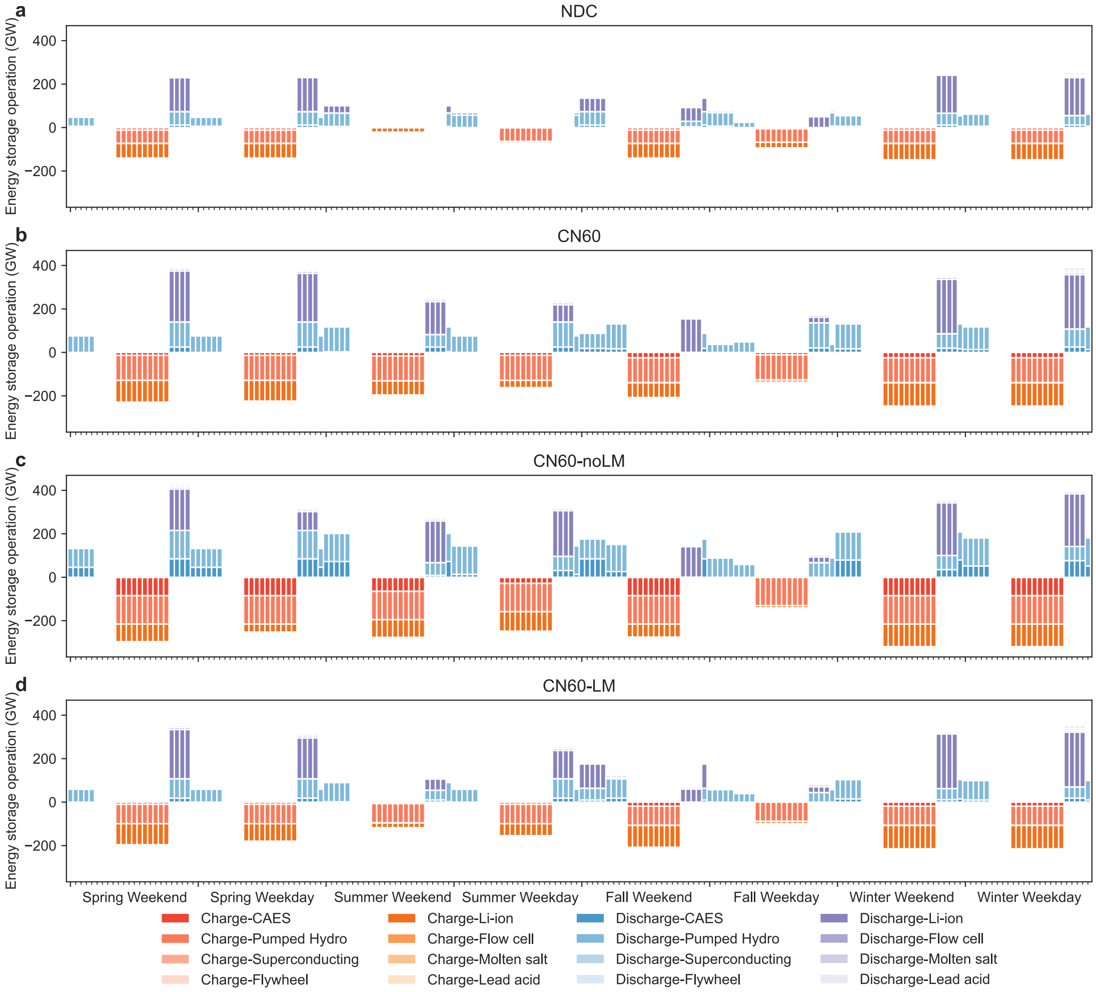

Supplementary Fig. 9 Energy storage facility operation mode in 2060 by technology for different seasons of the year and different working days

a Energy storage facility operation mode by technology under the NDC scenario. **b** Energy storage facility operation mode by technology under the CN60 scenario. **c** Energy storage facility operation mode by technology under the CN60-noLM scenario. **d** Energy storage facility operation mode by technology under the CN60-LM scenario. CAES represents compressed-air energy storage. GW represents gigawatt.

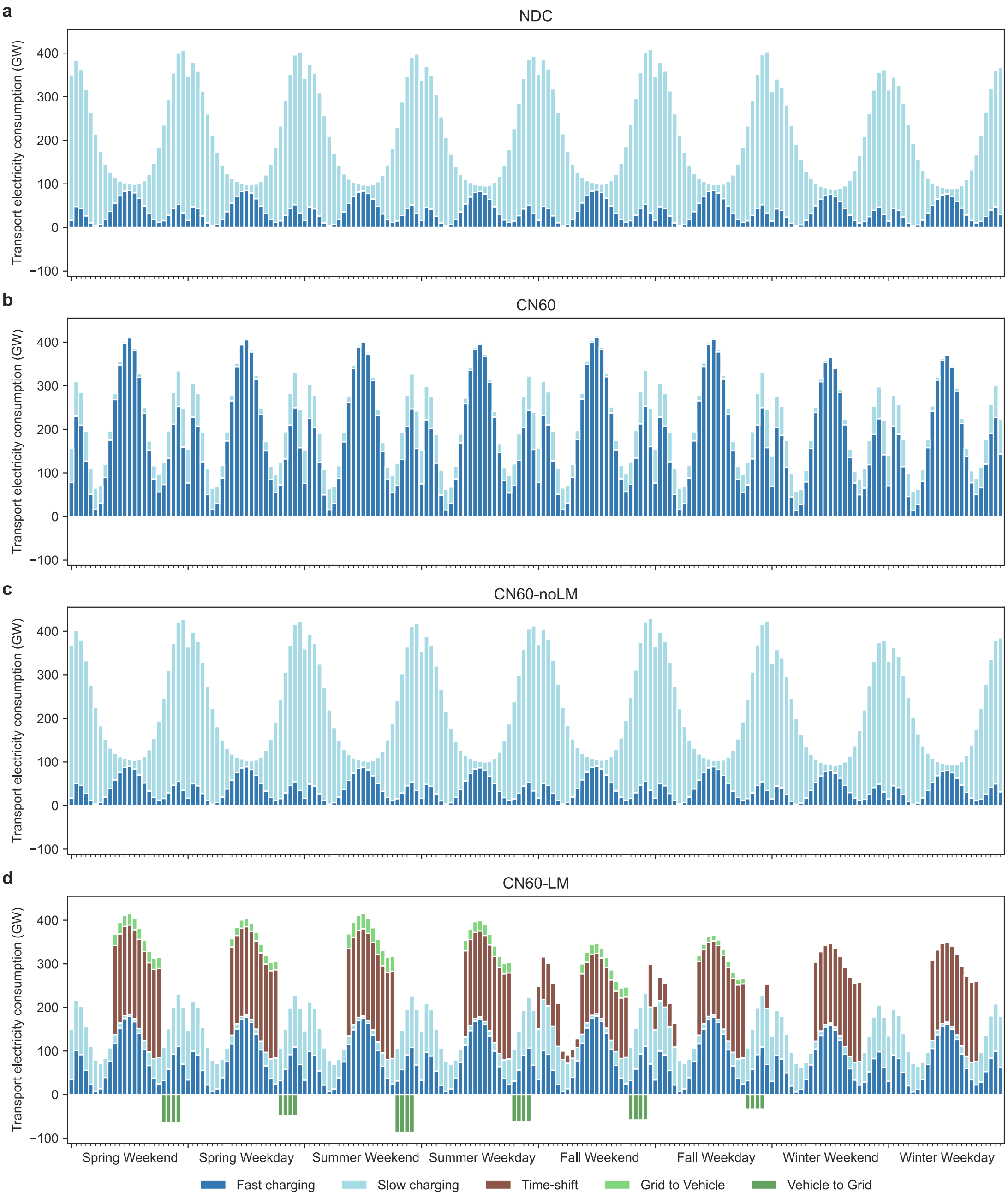

Supplementary Fig. 10 Electricity consumption in the transportation sector in 2060 by technology for different seasons of the year and different working days

a Electricity load in transportation sector by technology under the NDC scenario. **b** Electricity load in transportation sector by technology under the CN60 scenario. **c** Electricity load in transportation sector by technology under the CN60-noLM scenario. **d** Electricity load in transportation sector by technology under the CN60-LM scenario. GW represents gigawatt.

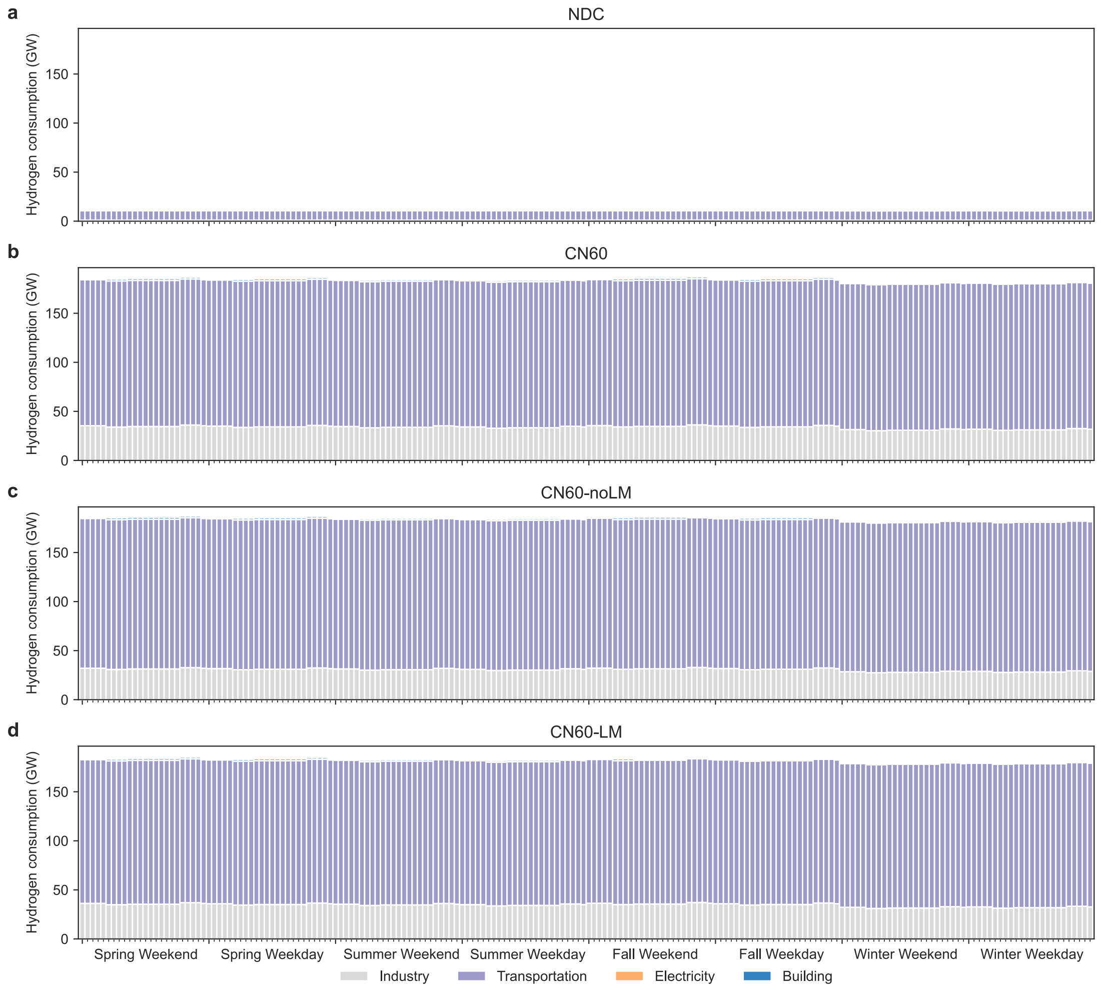

Supplementary Fig. 11 Hydrogen consumption by sector in 2060 for different seasons of the year and different working days

a Hydrogen consumption by sector under the NDC scenario. **b** Hydrogen consumption by sector under the CN60 scenario. **c** Hydrogen consumption by sector under the CN60-noLM scenario. **d** Hydrogen consumption by sector under the CN60-LM scenario. GW represents gigawatt.

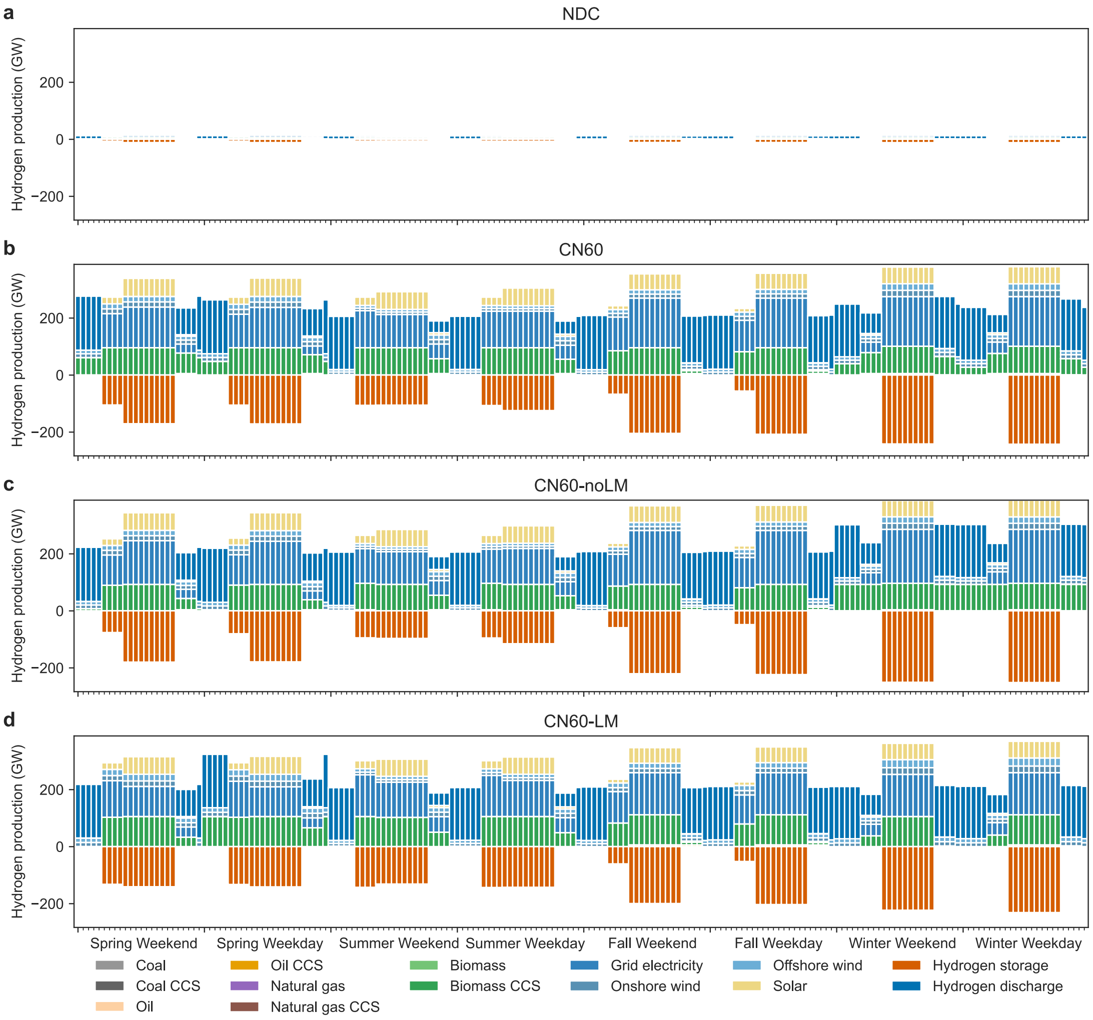

Supplementary Fig. 12 Hydrogen production by technology in 2060 for different seasons of the year and different working days

a Hydrogen production by technology under the NDC scenario. **b** Hydrogen production by technology under the CN60 scenario. **c** Hydrogen production by technology under the CN60-noLM scenario. **d** Hydrogen production by technology under the CN60-LM scenario. In the legend, the fuel names ending in CCS indicate hydrogen generations equipped with carbon capture and storage (CCS) and otherwise indicate hydrogen generations without CCS. GW represents gigawatt.

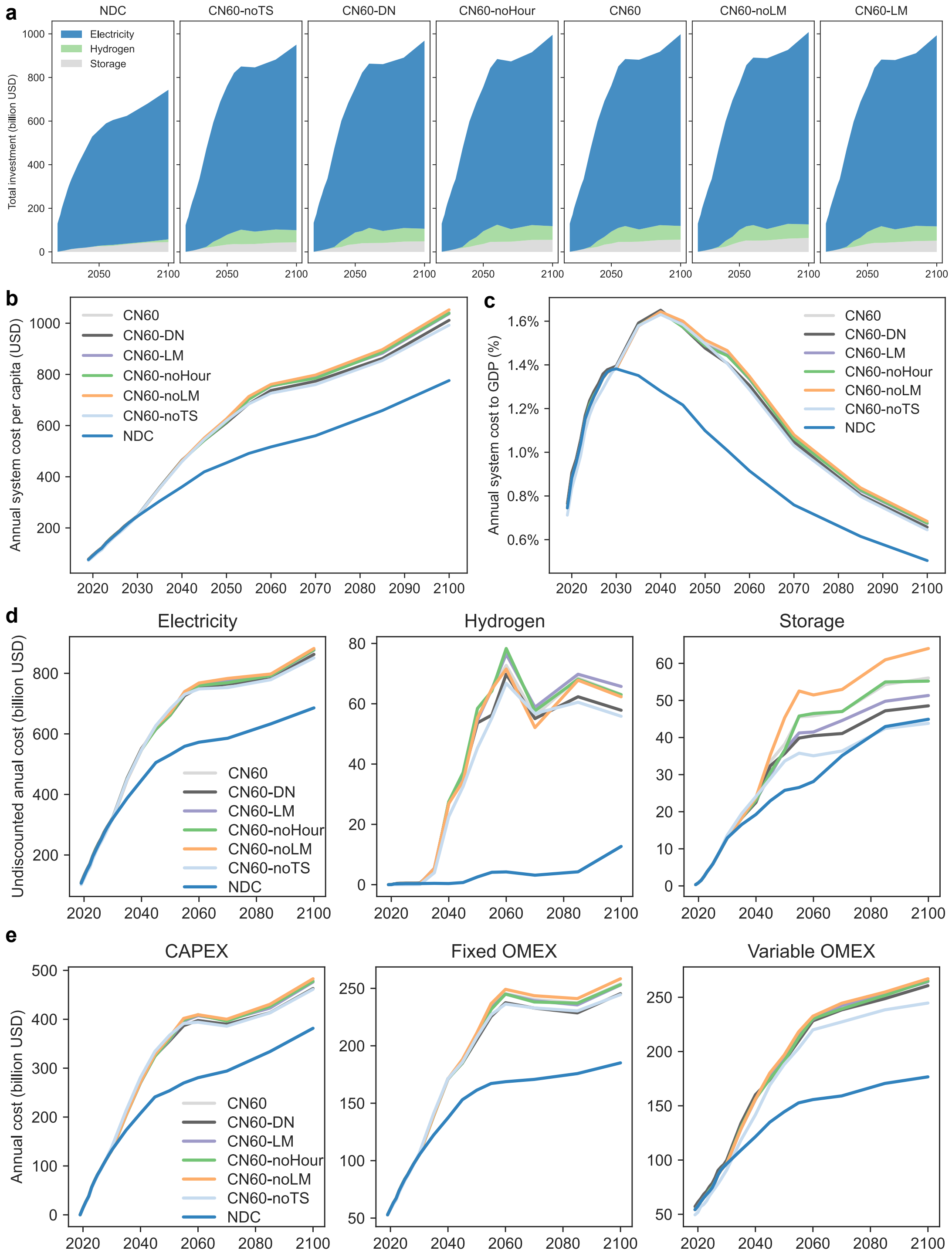

Supplementary Fig. 13 Energy transition cost in electricity, hydrogen and storage for China during 2020-2100

a Annual system investment. **b** Undiscounted annual per capita system cost. **c** Undiscounted annual system costs as a share of the year's GDP. **d** Undiscounted annual system costs by sector. **e** Undiscounted annual system costs by type of cost. USD represents US dollar.

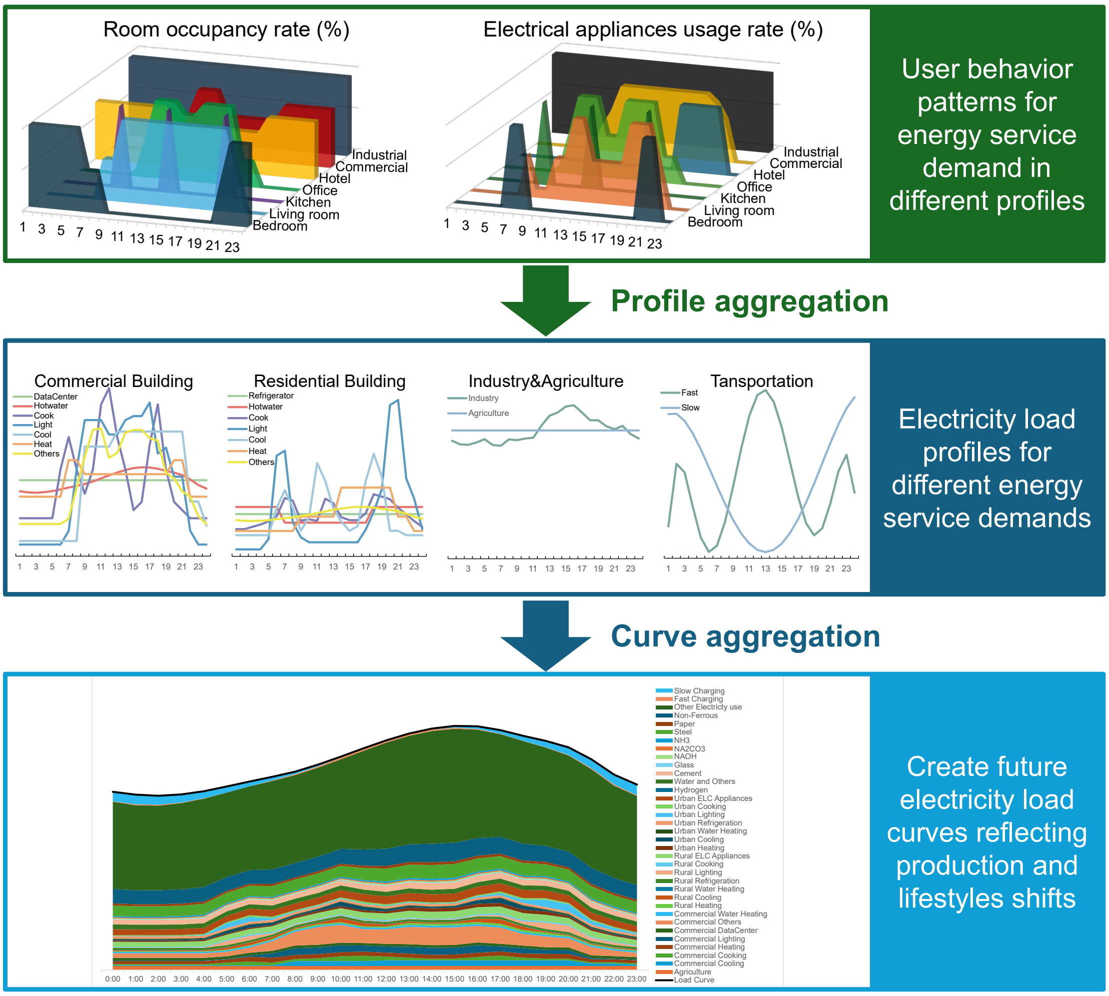

Supplementary Fig. 14 Behavior-based load curve generation procedure

The load curve generation in the China TIMES 2.0 is based on the behavior of energy consumption and obtains the intraday usage fraction for each demand, which is combined with the macroeconomically-driven energy service demand projections and the share of electricity in that demand to obtain the future electricity load profile for that demand. The various demand curves are eventually aggregated to form a system-wide power load curve. The load profile of the building sector refers to the national standard GB 55015-2021 “General specifications for building energy efficiency and renewable energy use”. The load profile of the transportation sector refers to the “New Energy Vehicle Industry Development Plan (2021-2035)”. The agriculture sector is assumed to have flat electricity use curve, and the total load residual term excluding building, transportation, and agriculture is considered as the electricity consumption curve for the industrial sector.

Supplementary Note 1 China's energy transition towards carbon neutrality

China is projected to reach its peak in fossil fuel and industrial processes (FFI) carbon emissions around 2025 at 12 $GtCO_2$ (Supplementary Fig. 15a). Unlike the rapid coal-fired power plant (CFPP) phase-out pathway proposed by most IAMs, our study shows the ongoing expansion of CFPPs would persist as renewables are inadequate in addressing the incremental demand for electricity. After 2030, the energy system is expected to undergo a marked transition, culminating in the achievement of CO_2 net-zero emissions by 2060. Achieving the desired climate targets necessitates a precipitous decline in the coal usage in unabated CFPPs and energy-intensive industries, which would lead to formidable transition pressures (Supplementary Fig. 15b). Final energy consumption is projected to rise by 16% from 2020 to 2030, followed by a gradual decline leading up to 2060 (Supplementary Fig. 15c). The expansion of electricity applications would lead to changes in both the quantity and temporal distribution of electricity consumption (Supplementary Figs. 1-5). Renewables are anticipated to dominate electricity generation after 2035 (Supplementary Fig. 15d). This shift necessitates an accelerated transition within the power sector.

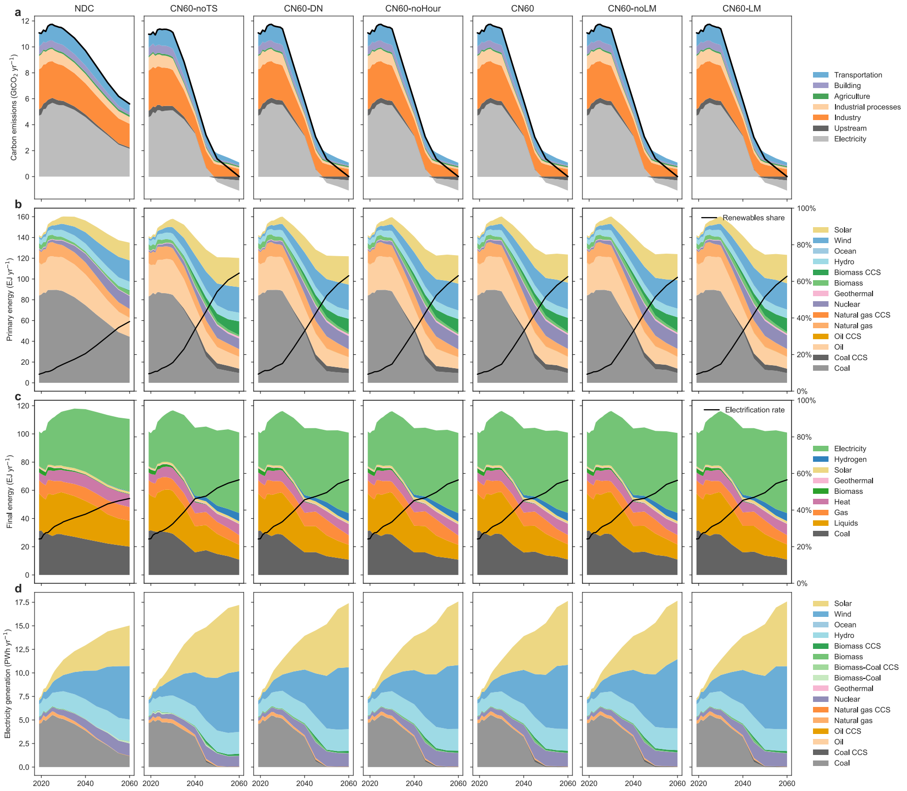

Supplementary Fig. 15 China's energy system decarbonization pathway for all scenarios
a Fossil fuel and industrial processes (FFI) CO_2 emissions by sector, **b** Primary energy mix by fuel, **c** Final energy mix by fuel, **d** Electricity generation by technology. In the legend of Panel **b**, the fuel names ending in CCS indicate fuels are used by processes equipped with carbon capture and storage (CCS) and otherwise indicate processes without CCS. In the legend of Panel **d**, the fuel names ending in CCS indicate power plants equipped with CCS and otherwise indicate power plants without CCS. $GtCO_2$ yr^{-1} represents billion tons of CO_2 per year. EJ yr^{-1} represents exajoule per year. PWh yr^{-1} represents petawatt-hour per year.

Supplementary Note 2 Sensitivity scenario analysis for flexibility retrofits

Flexibility retrofits can help fully utilize existing large-scale thermal power assets to provide flexibility to the energy system. The cost and pace of retrofitting will indeed have a certain impact on the development of thermal power and nuclear power. In the model, retrofit technologies are provided for coal-fired, nuclear, and biomass power generation units (both with and without CCS). Based on the CN60 scenario (retrofitting cost of 500 CNY kW^{-1}), we further ran two additional scenarios: CN60-lowCost and CN60-highCost, corresponding to retrofitting costs of 200 and 1000 CNY kW^{-1}, respectively.

Sensibility scenario simulations indicate that, given the relatively high investment costs associated with energy storage and other flexibility technologies, flexibility retrofits remain cost-effective for most power plants. In the CN60-highCost scenario, solar power generation is projected to decline moderately by 2060, while wind and nuclear power generation are expected to increase correspondingly. Over the 2020–2060 period, total electricity generation from all power sources will experience minor fluctuations. In the CN60-lowCost scenario, the impact on total electricity generation is minimal, with coal-fired power generation with CCS showing a moderate increase between 2045 and 2055.

From the hourly operation perspective, it can be seen that under the CN60-highCost scenario, daytime solar power generation will decrease, while wind power generation will increase slightly throughout the day. In the evening, gas-fired power plants and energy storage will play a greater role in providing flexibility. Under the CN60-lowCost scenario, some nuclear power plants will participate in flexibility renovations and engage in peak shaving, while wind power and solar power generation will not be significant. At the same time, activities related to flexible hydropower and energy storage have also decreased relatively.

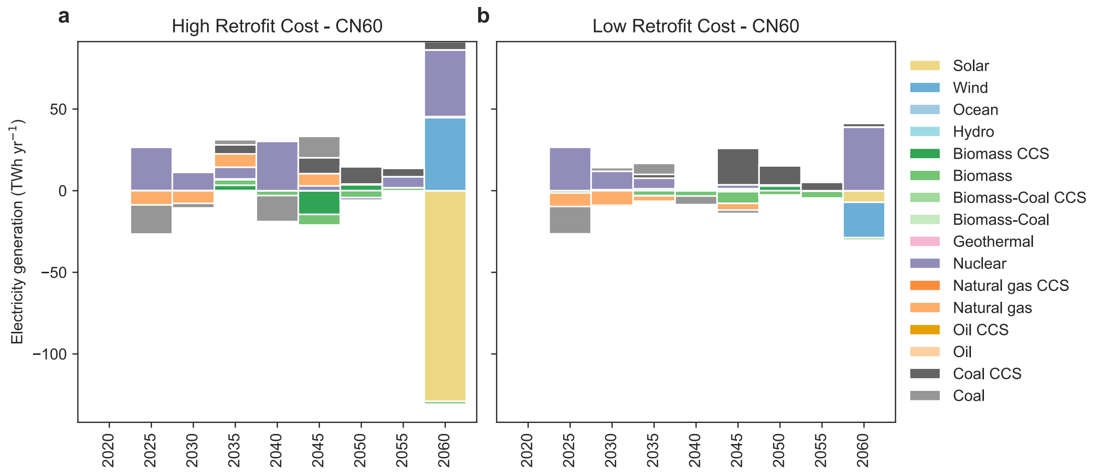

Supplementary Fig. 16 Differences in electricity capacity by technology between the flexibility retrofit sensitivity scenario and the core scenario

a Differences in electricity capacity by technology between the high retrofit cost scenario and the core scenario. **b** Differences in electricity capacity by technology between the low retrofit cost scenario and the core scenario. In the legend, the fuel names ending in CCS indicate power plants equipped with CCS and otherwise indicate power plants without CCS. TWh yr^{-1} represents terawatt-hour per year.

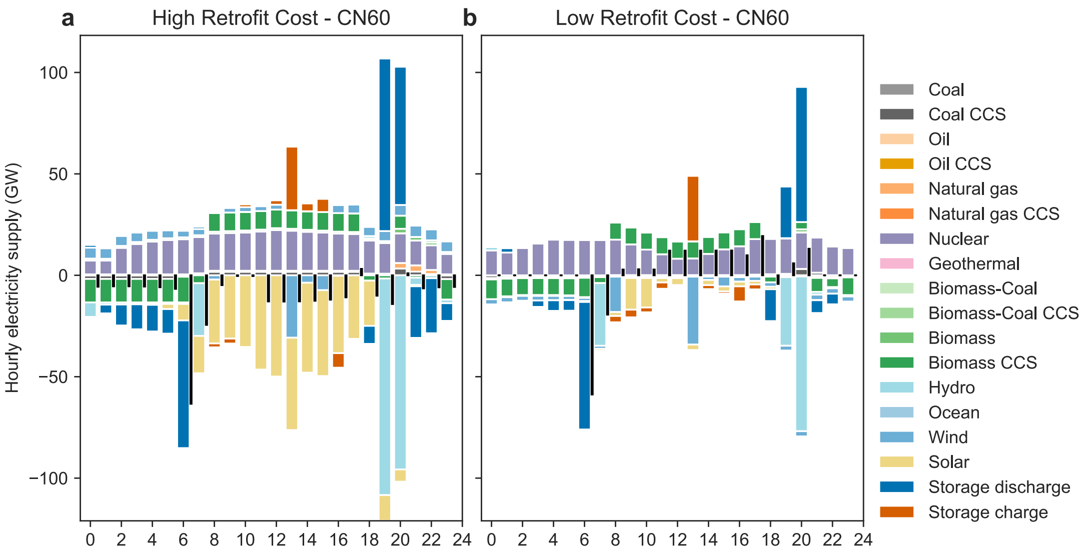

Supplementary Fig. 17 Differences in electricity supply by technology for the typical day in 2060 between the flexibility retrofit sensitivity scenario and the core scenario

a Differences in electricity supply by technology between the high retrofit cost scenario and the core scenario. **b** Differences in electricity supply by technology between the low retrofit cost scenario and the core scenario. In the legend, the fuel names ending in CCS indicate power plants equipped with CCS and otherwise indicate power plants without CCS. GW represents gigawatt.

Supplementary Note 3 Sensitivity scenario analysis for vehicle-to-grid application rates

Demand-side response, such as vehicle-to-grid (V2G), can alleviate peak loads and reduce energy storage requirements. Based on the CN60-LM scenario, we ran two additional sensitivity scenarios and made differentiated adjustments to the participation level of V2G. Specifically, we changed the proportion of the maximum activity level of V2G in each timeslice to the electricity consumption of the transportation sector in different scenarios. For the CN60-LM scenario, in 2030, 2050, and 2110, the maximum activity level for charging or discharging in each timeslice are 20%, 30%, and 50% of the original transportation electricity demand for that timeslice, respectively. For the LowV2G scenario, these limits are 20%, 20%, and 20%, respectively. For the HighV2G scenario, these limits are 40%, 60%, and 70%, respectively.

From the power supply on a typical day, a higher V2G application rate (HighV2G scenario) can promote the integration of wind power and solar power at noon (approximately 50GW), while reducing the charging and discharging of energy storage. V2G facilities primarily charge during midday and discharge in the evening. Conversely, the LowV2G scenario significantly weakens the role of V2G, resulting in a noticeable flexibility shortage in the evening, necessitating increased output from various power plants such as nuclear power, coal-fired power, and biomass-fired power to provide flexibility.

Focusing on V2G operations, it can be observed that the primary charging period for V2G vehicles is between 8 and 10 am, while the discharge period is between 8 and 9 pm. By increasing the utilization rate of V2G applications, charging during the 9 am to 2 pm period can be significantly enhanced, while discharge during the 6 pm to 11 pm period also sees an overall improvement. Through sensitivity analysis, it can be summarized that the most economical role of V2G is to provide power support during peak load and ramping periods. Under conditions of higher V2G application rates, it can partially replace the functions of energy storage facilities.

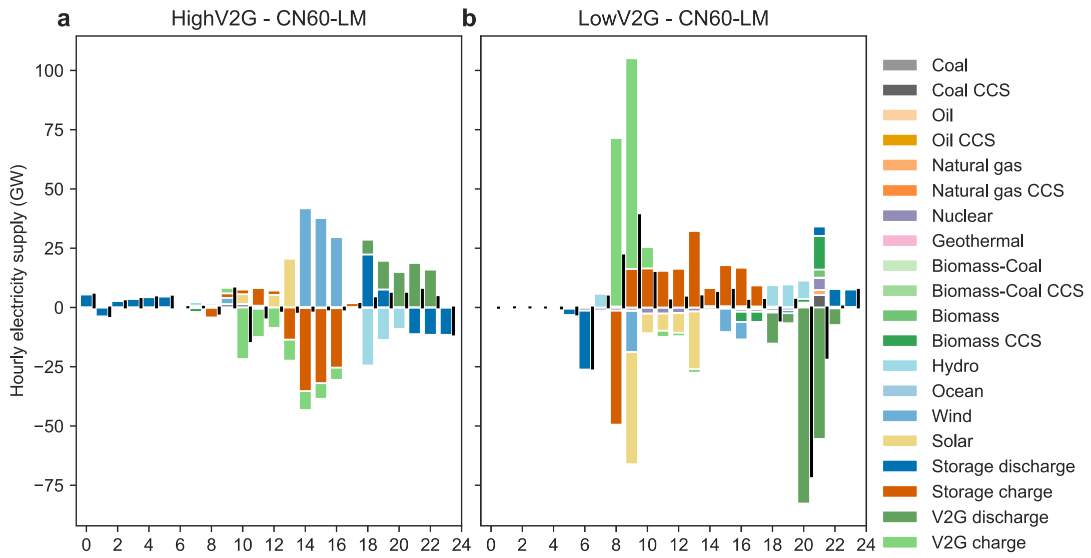

Supplementary Fig. 18 Differences in electricity supply by technology for the typical day in 2060 between the vehicle-to-grid application rate sensitivity scenario and the core scenario

a Differences in electricity supply by technology between the high vehicle-to-grid (V2G) application rate scenario and the core scenario. **b** Differences in electricity supply by technology between the low V2G application rate scenario and the core scenario. In the legend, the fuel names ending in CCS indicate power plants equipped with carbon capture and storage (CCS) and otherwise indicate power plants without CCS. GW represents gigawatt.

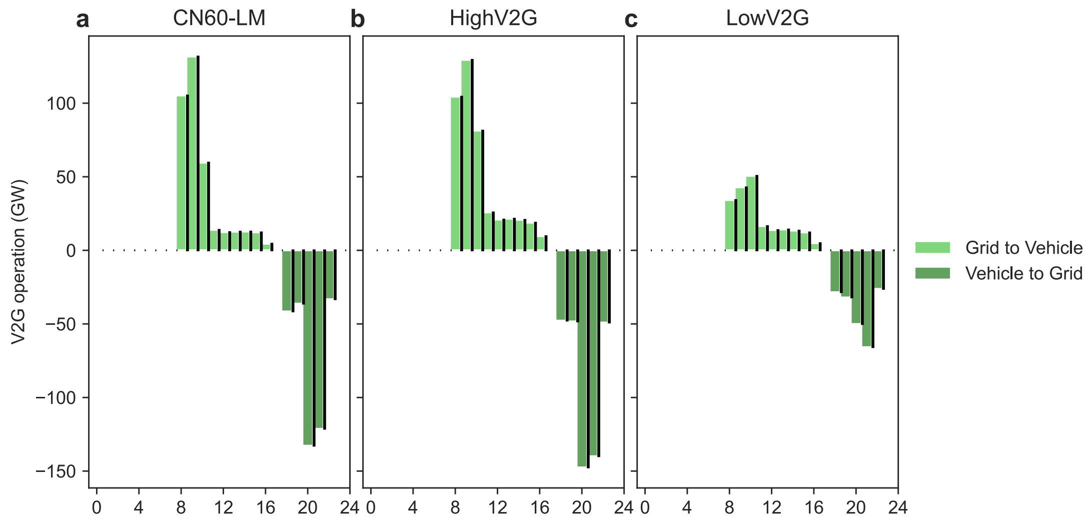

Supplementary Fig. 19 Vehicle-to-grid operations in 2060 under different scenarios

a Vehicle-to-grid (V2G) operations under the CN60-LM scenario. **b** V2G operations under the high V2G application rate scenario. **c** V2G operations under the low V2G application rate scenario. GW represents gigawatt.
