# Supplementary material for: Exploring the feasible net-zero transition pathway in China considering energy system flexibility
Source: Nat Commun. 2026 Apr 11;17:5440. doi: 10.1038/s41467-026-71410-2 (PMC13279959; doi:10.1038/s41467-026-71410-2)
Supplement: Supplementary file 2 — Transparent Peer Review file [file 41467_2026_71410_MOESM2_ESM.pdf]

nature portfolio

Peer Review File

Exploring the feasible net-zero transition pathway in China considering energy system flexibility

Corresponding Author: Professor Wenying Chen

This file contains all reviewer reports in order by version, followed by all author rebuttals in order by version.

Version 0:

Reviewer comments:

Reviewer #1

(Remarks to the Author)
This paper proposes the China TIMES 2.0 model, which significantly enhances the temporal granularity of traditional IAMs by introducing a nested temporal resolution and 56 representative timeslices. This framework provides a technical foundation for capturing energy system flexibility requirements (e.g., intraday fluctuations, seasonal variations). Overall, this paper demonstrates innovation and advances the existing literature. However, revisions are required before acceptance. Here are some comments I would incorporate into the paper.

1) The main contribution of this paper is the presentation of a sub-annual modeling framework within a national IAM. However, the authors do not sufficiently elaborate on this advantage in the results section. In this regard, the most important result is Extended Data Fig. 1. It can be observed that most subplots in this figure show that there is no significant differentiation between the proposed model and other annual IAM models. In fact, for some indicators, the trends are almost identical between the paper's model and other models. The authors should expand their discussion on model comparisons to better highlight the advantages of their proposed model.
2) On page 11, the authors mention that "Similarly, centralized PV, distributed PV, building-integrated PV (BIPV), and centralized solar power (CSP) set future sub-annual operations based on hourly data in 2018". Given that the model's base year is 2019, using 2018 data for PV and wind hourly generation profiles introduces potential temporal misalignment. If updated data are available, the inputs should be harmonized. If not, a justification for using 2018 data must be added.
3) Model validation against recent trends. The model starts planning pathways from 2019. Given that actual data for the past five years (2020-2024) may be available for indicators such as energy consumption, CO2 emissions, and renewable energy generation, a key question arises: which modeling pathway best aligns with recent trends? Addressing this question would strengthen the model's empirical credibility and its relevance to real-world transitions.
4) Parameter sensitivity analysis. The model relies on numerous predefined parameters. To assess robustness, the authors should: (a) Identify key parameters that distinguish this model from others (e.g., demand response elasticity, storage cost assumptions). (b) Conduct sensitivity analyses (e.g., Monte Carlo simulations) to quantify how uncertainties in these parameters affect outcomes (e.g., marginal abatement costs, technology portfolios).
5) Page 6. The text cites "Fig. 5d", but Figure 5 does not include a panel labeled "d".

Reviewer #2

(Remarks to the Author)
The paper should be improved by addressing the following comments.
1. Whether the model covers all factors and contingencies, response to special circumstances such as extreme weather, policy changes, etc. There is a need for further in-depth analysis of technological development uncertainties, such as the expansion of the scale of renewable energy from the point of view of renewable energy systems, the rate of decline in the cost of renewable energy technologies, and breakthroughs in energy storage technologies.
2. Feedback on how the impacts of different policies and market mechanisms on the flexibility of the energy system will feed into forecasts and be accurately predicted.
3. How is the balancing of peak load shifting for V2G with the utilization of renewable energy sources such as PV during daytime peaks considered.

4. The sub-annual time model constructed is divided into: annual-seasonal-weekly-daily-hourly, whether energy flexibility is taken into account for over-seasonalization, and why not annual-monthly-weekly-daily-hourly and consider a more detailed division of time even down to the minute.

Reviewer #3

(Remarks to the Author)
This paper sets out to improve the temporal resolution of the China TIMES model, arguing that such models typically fail to, due to limited representation, capture the necessary flexibility needed for higher renewable systems. In doing so, the study makes a valuable contribution by enhancing the time-slice granularity of the China-TIMES model, strengthening its capability to represent time-varying electricity load curves, proactive demand response, and the interaction between demand and supply. The authors present comprehensive results illustrating China's energy transition under its net-zero targets, considering system flexibility and financial implications.

However, there is a question as to whether the analysis is novel enough to meet the high bar for acceptance in a journal such as Nature Communications. There are other examples of IAM and energy system models running at higher temporal resolution. Therefore, further development of the argument regarding novelty is required.

Furthermore, the paper needs to bring stronger clarity to the difference between the 'standard' model and improved model. It was not clear from the scenario descriptions as to the key scenarios that were being compared, although this could be partly deciphered from Figure 2. Also, key results need to feature in the abstract – to headline the main findings. Additional information is needed in the main paper (incl. Methods) as to what the 'noTS' scenario means. This is not described; if it is simply a scenario that has no timeslices (TS) at all then this is less interesting, but if it was the previous version of China TIMES that had some more aggregated TS then that would be a useful comparison.

With the model effectively demonstrating the role of transportation time-shifting load and the potential of various short- and long-term storage technologies in electricity supply - both of which are critical components of your results - it is necessary to understand the prior baseline before the improvements were made.

There is also the question of scenario design. While the study constructs three net-zero scenarios with varying levels of demand-side participation, the overarching net-zero constraints seem to dominate the energy transition dynamics, thereby weakening the impact of demand-side management assumptions. As a result, the authors should clarify their scenario assumptions or revise the scenario design to align more closely with this granular modelling approach. This would help better illustrate the marginal impacts of the sub-annual analysis on the national net-zero transition.

In general, some of the language could be tightened up, and would benefit from further proof reading.

Abstract:
There is a need for some headline figures in here that underpin the main arguments about improved temporal resolution. What would you consider to be the main findings in quantitative terms?

Main:
Line 37. Define what you mean by 'IAM'. Many modellers would view China TIMES as an integrated energy systems model, not an IAM.

Line 41-42. Why does this result in 'casting doubts'?

Line 64-65. You state that "Our findings indicate that current IAM model does not capture well the challenges and opportunities of accessing large-scale VRE and electrical appliances." What 'model' are you referring to – China TIMES or IAMs in general? Is this context to your research rather than your finding?

Line 66-71. The energy demand temporal disaggregation proposed in this paper does not seem to directly address the obstacles mentioned - "fragmented regulatory frameworks, limited economic incentives, and insufficient market mechanisms." Could you clarify what the proactive demand response strategies refer to in your scenarios or modelling? Additionally, what policy recommendations can be derived from this analysis?

Results:

Energy Transition:

Line 76-81. Despite some description in Methods, more elaboration is needed here prior to reading the results. This includes for 'noTS', which is not described. What does 'orderly energy consumption' mean?

Figure 1 would benefit from including the 'noTS' scenario for completeness - although perhaps may not show key differences due to the results being presented at an annual level.

Line 82-95. Most modelling results presented here, along with the corresponding figures in your supplementary document, indicate no pronounced differences among your three CN60 scenarios. As I understand, China's key energy transition trends

—such as the emissions peak, energy consumption peak, and declining trajectory—are primarily determined by your net-zero constraints or carbon budget constraints. Could you clarify how your different demand- and supply-side strategies (key modelling assumptions) applied in your scenarios influence China's net-zero transition pathway?

Additionally, what new insights does your modelling improvement provide for China net zero transition?

As shown in Figure 2, there is a significant difference between sub-annual and annual modelling analyses across the aspects you mentioned. However, this impact remains generally consistent across the three CN60 scenarios (e.g., sectoral electricity demand). As I understand, these variations cannot be attributed to the effects of more granular and flexible demand-side management on the net-zero transition. Instead, they likely reflect structural uncertainties in this TIMES modelling results. Therefore, I am not convinced that this sub-annual analysis demonstrates any substantial impact on the energy transition itself. Additionally, in your comparison with other modelling analyses, I recommend clarifying the key differences in scenario assumptions between your analysis and other harmonized scenarios - particularly regarding demand-side input assumptions, which may contribute more significantly to the differences you present than the variations in time slices.

Line 108-124. Can you be a bit more precise as to the scenarios that are being compared in this section?

Flexibility:
Line 136-148. As shown in Figure 3c and 3d, the role of nuclear and hydropower storage shows generally consistent patterns in both NDC scenario and net zero scenarios. You then mention “However, cost and security considerations still limit its contribution to flexibility”. Could you clarify if you impose any constraints on the power sector, or if it is the model choice based you carbon budget constraints? If it is, I would recommend showing your fundamental assumptions around the power sector in the SI.

Line 141. Can you evidence the statement – ‘its flexible operation has gained momentum in both research and policy perspectives’? What does this mean?

Line 169-170. Can you state what you are comparing to when discussing the EV charging shift?

Line 174-175. You state that “Concurrently, the load profile undergoes a transformation from a relatively flat distribution to one characterized by daytime peaks, thereby aligning more effectively with the PV power generation”. Which load profile do you mean? Could you refer to the corresponding figure here?

Line 185-186. You write that “Nowadays, fossil fuels and district heating are the primary sources for satisfying the heating demand, while electricity almost exclusively fulfils cooling needs.” Is that district heating also fuelled by fossil fuels (e.g. Gas) in China?
Demand-supply interaction:

The figures cited in the text are inconsistent with the actual figures. Figure 5b is electricity consumption in the transportation sector on a summer workday, while Figure 5d is missing. Figure 6e is marginal electricity supply costs for different time periods on a summer workday.

Line 224-230 (line 459-468). Provide more detail on how demand shifting is achieved within the modelling framework. Price elasticity reducing demand level is not shifting the load. Is demand shifting to that described by Li et al. - https://www.sciencedirect.com/science/article/pii/S0306261918310237. More elaboration is needed.

Financial implications:
As with other sections, clarity on understanding the additional implications of improved temporality on system costs would be useful.

Discussion:
Develop this section more towards the implications of the results for decision makers, and for the modelling community.

Line 273-274. ‘Most economical’ compared to what other options?

Line 279-289. While interesting, are these results pertinent to the main argument of the paper?

Scenario setting:
The scenarios are distinct but need more clarification of common assumptions and differentiated assumptions, specific questions include:

Line 470-423. In your NDC scenario, does it include over 65% reduction in carbon intensity and other renewable related target?

Line 476-477. The statement “We set the cumulative carbon emissions for 2019-2100 under RCP2.6 assumption based on the carbon budget allocation principle (230 GtCO2)”, needs reference or clarification of the allocation principle, and why is it applied for all your scenarios?

Supplementary document:

SI Figure 2, why does coal use rebound in 2060 in urban areas within the hot summer–cold winter zone under your NDC scenario? However, in the same scenario and climate zone, coal use continues to phase down after 2030.

SI Figure 6 lacks legend

SI Figure 15 needs more elaboration around data source

Reviewer #4

(Remarks to the Author)
I co-reviewed this manuscript with one of the reviewers who provided the listed reports. This is part of the Nature Communications initiative to facilitate training in peer review and to provide appropriate recognition for Early Career Researchers who co-review manuscripts.

Version 1:

Reviewer comments:

Reviewer #1

(Remarks to the Author)
This version has been properly revised.

Reviewer #2

(Remarks to the Author)
The following comments should be considered for the revised manuscript:
1. Sensitivity analysis should be conducted considering different V2G participation rates in the assumptions of demand-side response?
2.The sensitivity analyses should also consider different assumed costs for the flexibility retrofits of coal-fired power plants?

Reviewer #3

(Remarks to the Author)
Thank you for the authors' detailed and thoughtful responses to the first-round comments. I appreciate the substantial effort made to address the issues raised, including the additional analyses, clarifications, and the inclusion of new tables and data where requested. These revisions have strengthened the manuscript considerably and improved its transparency.

In response to comments 1.1 and 3.8 regarding the paper's novelty, the three core comparisons presented are clear. However, I remain unconvinced, as your sub-annual modelling approach and key quantitative findings primarily demonstrate that traditional annual-based IAM scenario analyses tend to idealize the pace of thermal power phase-out and overestimate the scale of renewable deployment—an issue that is already widely acknowledged. That said, the strength of your model in exploring specific pathway combinations can indeed support China in identifying more feasible power system transition pathways. I would therefore recommend positioning the paper's novelty around the feasibility of these transition pathways with flexibility management included, rather than framing sub-annual modelling itself as the core innovation. If so, I also suggest refining your key quantitative conclusions in the abstract accordingly.

Part of the problem of the current argument of novelty relates to the starting position of the paper, and the scenario design. Under comment 3.4, the statement on the lack of temporal resolution in models needs to be evidenced. You use one reference (8) in lines 48-49, but this paper is not relevant and fails to back this assertion up. Most national TIMES models for example will almost certainly be using a sub-annual timeslice resolution – so this starting position for the paper is not accurate (unless you have references to suggest otherwise). A stronger position would be that while there are some attempts at sub-annual representation in similar models, this paper is arguing for improving this, based on enhanced modelling of temporal dynamics.

This then calls into question the scenario design, where you have used a 'noTS' scenario. The problem of comparison against this scenario is undermined if indeed most national TIMES models do have some level of TS resolution. Comparisons of scenarios with and without TS, and the emerging results, are therefore not surprising – given that you are using quite an extreme comparator i.e. the noTS case.

Under 3.6, you state that for noTS 'Such a scenario is equivalent to the scenario of a traditional annual-level IAM model without embedded sub-annual level inscriptions.' Is this really the case? Again, please evidence this based on equivalent national models.

The novelty of the paper is likely to be in the innovative way you have introduced load shifting and V2G integration.

However, the approach to identifying any insights based on a comparison with noTS is problematic, and doesn't allow you to highlight improvements versus standard modelling approaches.

A further concern in this revised manuscript relates to the writing and overall structure. The authors have commendably added a great deal of information to clarify the questions raised by reviewers, which is much appreciated. However, the manuscript as a whole would benefit from further proofreading to ensure it is concise enough and better aligned with your research focus and the key conclusions emerging from the analysis. Especially in the Results section, the presentation of quantitative findings does not fully reflect the structure and insights expected from your three comparisons. In other words, the presentation could be more clearly organized or restructured to better convey the main findings.

Key suggestions include (but are not limited to):

Section: Assessing the energy transition at the sub-annual level
• Since the key focus of the paper is the flexibility of the power sector transition, and the sub-annual model does not show significant differences in transition pathways in broad terms, I recommend tightening this section. Keep the key results concise and highlight where the sub-annual modeling truly makes a difference.

• Lines 114–118: "Fig. 2 explores the implications of considering sub-annual modeling across the dimensions of mitigation strategy, energy demand, energy supply, dispatchable resource, and cost & effort. The sub-annual analysis indicates that achieving the energy transition with a high VRE share and a high electrification rate would pose greater challenges than that from the annual perspective."

This sentence appears to suggest that sub-annual analysis poses greater challenges to the energy transition itself, which is confusing. Sub-annual modelling is a change in the granularity of time, and while it may reveal operational challenges, it does not in itself create obstacles to transition pathways.

• Lines 134–136: "The scenario with sub-annual modelling would increase marginal abatement costs of CO2 by more than 9% and decrease total energy consumption by 3% in 2060 compared to that without sub-annual modelling." This has a similar issue: it implies the modeling choice causes these differences rather than reveals them through a more refined temporal lens. I suggest reframing to clarify.

Section: Variable renewable energy diffusion leading to flexibility scarcity
• The context in Lines 158–162 could be tightened.

• Focus more directly on results or metrics that clearly support the subtitle's theme—i.e., highlighting the scarcity of flexibility in a high-VRE system.

• Lines 183–187: "In the context of seasonal variations in electricity supply, summer is distinguished by the longest periods of daylight, resulting in PV generation contributing ~48% of the electricity mix. Conversely, during the winter and spring months, wind energy assumes a more prominent role..." This provides seasonal context on VRE availability, but the link to "flexibility scarcity" is unclear. Consider reframing this to focus on how these variations create flexibility challenges.

• Lines 189–191: "The CN60-noLM scenario demonstrates higher levels of storage activity in comparison to the other two net-zero scenarios, indicating that orderly demand could alleviate power balancing challenges (Fig. 6a)." This content is more appropriate for the next section (Orderly demand easing power imbalance) and should be moved there for better flow.
• For the sections Orderly demand easing power imbalance and Addressing the flexibility crisis through demand-supply interaction, there is a similar issue of attempting to cover too many quantitative details, which results in a lack of clear focus in the discussion. I would suggest the authors to further refine the narrative structure in these sections to strengthen the logical flow and better highlight the key insights.

Reviewer #4

(Remarks to the Author)
I co-reviewed this manuscript with one of the reviewers who provided the listed reports. This is part of the Nature Communications initiative to facilitate training in peer review and to provide appropriate recognition for Early Career Researchers who co-review manuscripts.

Version 2:

Reviewer comments:

Reviewer #2

(Remarks to the Author)
This version has been properly revised.

Reviewer #3

(Remarks to the Author)
I would like to thank the authors for the careful consideration of the comments provided. In my opinion, the authors have responded adequately to the suggestions made. I would therefore recommend publication.

Reviewer #4

(Remarks to the Author)
I co-reviewed this manuscript with one of the reviewers who provided the listed reports. This is part of the Nature Communications initiative to facilitate training in peer review and to provide appropriate recognition for Early Career Researchers who co-review manuscripts.

Open Access This Peer Review File is licensed under a Creative Commons Attribution 4.0 International License, which permits use, sharing, adaptation, distribution and reproduction in any medium or format, as long as you give appropriate credit to the original author(s) and the source, provide a link to the Creative Commons license, and indicate if changes were made.
In cases where reviewers are anonymous, credit should be given to 'Anonymous Referee' and the source.
The images or other third party material in this Peer Review File are included in the article's Creative Commons license, unless indicated otherwise in a credit line to the material. If material is not included in the article's Creative Commons license and your intended use is not permitted by statutory regulation or exceeds the permitted use, you will need to obtain permission directly from the copyright holder.
To view a copy of this license, visit https://creativecommons.org/licenses/by/4.0/

Response to Referees

NCOMMS-25-08417-T

June 25, 2024

Reviewer 1

General Comment:

This paper proposes the China TIMES 2.0 model, which significantly enhances the temporal granularity of traditional IAMs by introducing a nested temporal resolution and 56 representative timeslices. This framework provides a technical foundation for capturing energy system flexibility requirements (e.g., intraday fluctuations, seasonal variations). Overall, this paper demonstrates innovation and advances the existing literature.

Response:

Thank you very much for reviewing this paper and for your valuable comments, we will improve the paper based on the suggestions you have provided, hoping that it will convey our intentions more clearly to the reader.

Comment 1.1:

The main contribution of this paper is the presentation of a sub-annual modeling framework within a national IAM. However, the authors do not sufficiently elaborate on this advantage in the results section. In this regard, the most important result is Extended Data Fig. 1. It can be observed that most subplots in this figure show that there is no significant differentiation between the proposed model and other annual IAM models. In fact, for some indicators, the trends are almost identical between the paper's model and other models. The authors should expand their discussion on model comparisons to better highlight the advantages of their proposed model.

Response:

Thank you very much for your valuable comments. In this paper, we discuss three comparisons: 1) a comparison between China TIMES and other IAMs under the harmonized scenario proposal; 2) a comparison of scenarios in which China TIMES considers sub-annual modeling or not; and 3) a comparison of scenarios in which China TIMES considers different degrees of demand-side management.

For the first comparison, the Extended Data Figure 1 (Figure 3 in the revised version)

that you referenced, all models optimize pathways given the net-zero constraint in 2060 and the same cumulative carbon emissions cap. As a result, the models show nearly identical trends in indicators such as CO_2 emissions, renewable energy share, and electrification rate, which suggests that the models are largely harmonized. However, due to the unique role of thermal power, green hydrogen, and energy storage for system flexibility, their trajectory projections have been differentiated from other models. Take thermal power as an example, thermal power that offers flexibility, enabling reduced investment in storage and avoiding stranded assets in existing units by 2040, also leads to a delay in the decline of emissions in the power sector.

For the second comparison, Figs. 1, 2 and Supplementary Fig. 13 show the impacts from considering sub-annual modeling especially in terms of dispatchable resources, PV capacity, hydrogen and storage investments and welfare losses.

For the third comparison, it can be seen from Figs. 4-8 and Supplementary Fig. 13 that demand-side management measures can have an impact on cross-sectoral energy and financial flows, due to the clear impact on energy demand in the transportation sector, investments in energy storage such as pumped hydro, marginal electricity and hydrogen prices, etc.

In order to further demonstrate the novelty of our paper, we have revised the results section, while we have redrawn Fig. 1 and Supplementary Fig. 13 with the addition of the CN60-noTS scenario, which illustrates the impacts of sub-annual modeling from the perspective of energy and financial flows, respectively.

Changes/additions to the manuscript:

(Manuscript, Line 93-99) For comparison, we also present a scenario without sub-annual constraints, CN60-noTS, which removes the seasonal, weekly, and daily variations in renewable energy output, load carve-outs (assuming an average distribution) and relaxes the power plant flexibility constraints. Energy storage and demand-side response measures are also not considered. Such a scenario is equivalent to the scenario of a traditional annual-level IAM model without embedded sub-annual level inscriptions.

(Manuscript, Line 118-127) Fig. 3 compares the results with prominent IAMs under harmonized scenario framework. China TIMES and global IAMs ran the Glasgow+ scenario under the ENGAGE project protocol to extend the net-zero target to all countries based on income level, after considering countries already proposing a net-zero target (Supplementary Table 4). All models set the same carbon budget constraints according to the equal cumulative per capita method, which ensures that the outputs are comparable. The converging trends in carbon emission pathways,

renewables share, and electrification rates show that the models are largely harmonized. However, due to the unique role of thermal power, green hydrogen, and energy storage for system flexibility, their trajectory projections have been differentiated from other models.

(Manuscript, Line 143-145) Thermal power achieves a shift from electricity provider to flexibility provider, mitigating the possible shocks of rapid decommissioning, which is unique to the sub-annual perspective.

(Manuscript, Line 147-151) Comparing the three sub-annual scenarios, the demand-side measures have cross-sectoral impacts, in particular significant impacts on power transformation and energy storage development. Energy storage is an important source of system flexibility, both for frequency and voltage support as well as for energy transfer.

(Manuscript, Fig.1) add CN60-noTS scenario

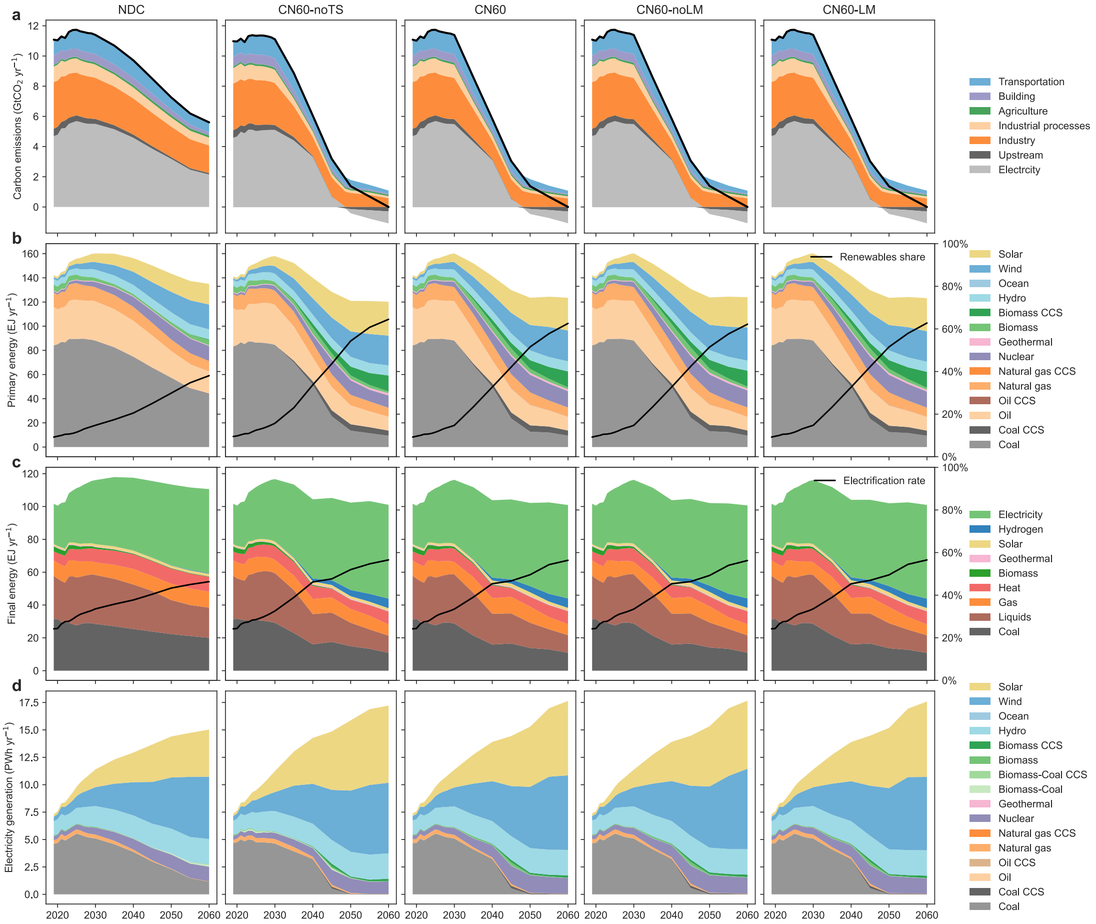

Fig. 1 China's energy system decarbonization pathway

(Supplementary Information, Supplementary Fig.13) add CN60-noTS scenario

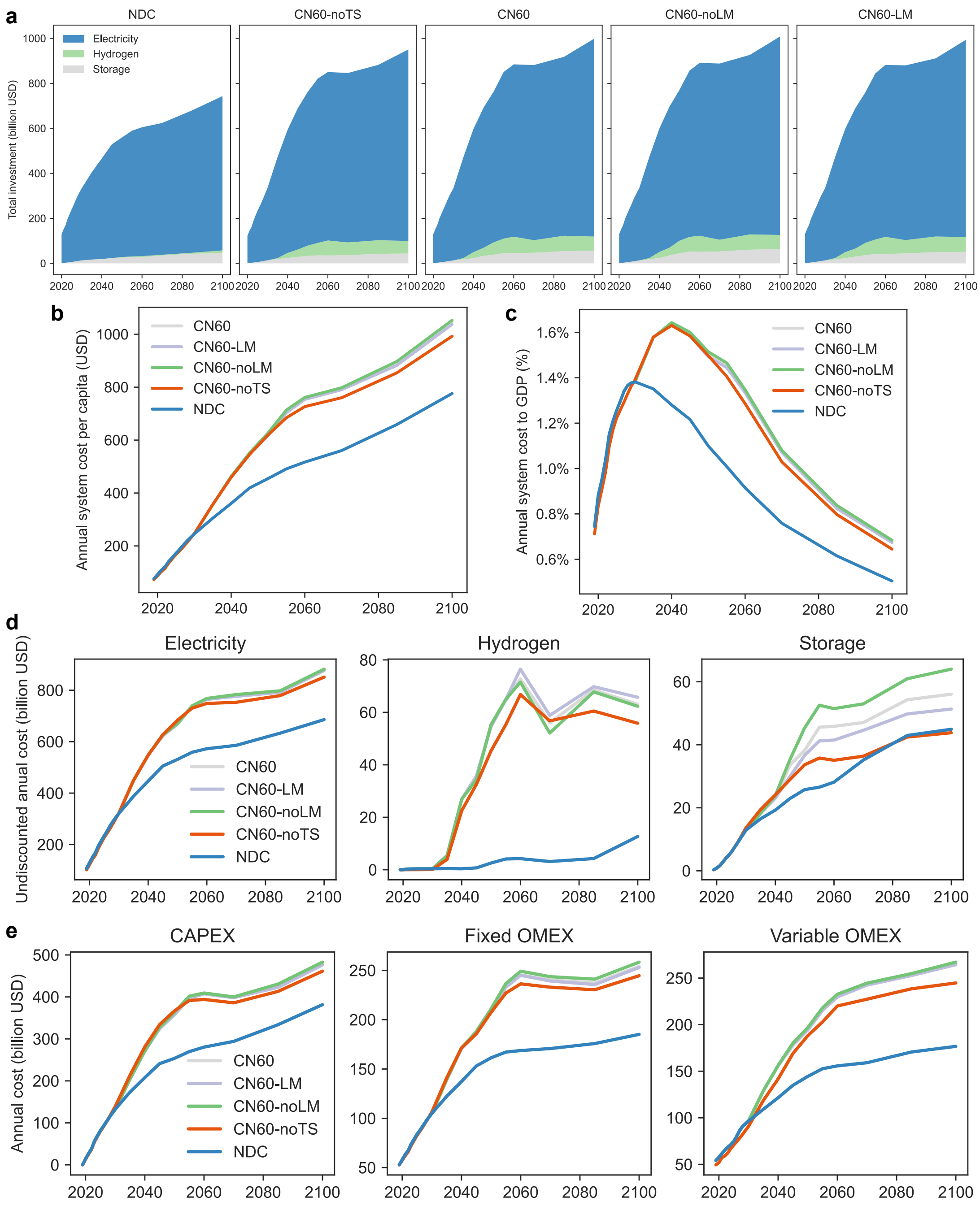

Supplementary Fig. 13 Energy transition cost in electricity, hydrogen and storage for China during 2020-2100.

Comment 1.2:

On page 11, the authors mention that "Similarly, centralized PV, distributed PV, building-integrated PV (BIPV), and centralized solar power (CSP) set future sub-annual operations based on hourly data in 2018". Given that the model's base year is 2019, using 2018 data for PV and wind hourly generation profiles introduces potential

temporal misalignment. If updated data are available, the inputs should be harmonized. If not, a justification for using 2018 data must be added.

Response:

Thank you very much for your question. In view of the limitation of data access, the current data on load, wind power and PV annual hourly levels are all referred from the published paper in Nature Communications, and the statistics department does not regularly publish the hourly generation and load information, so it is not yet possible to obtain the relevant data for 2019.

For this reason, instead of feeding the raw data directly into the model, we pre-processed the data to find the hourly PV and wind generation and the load as a percentage of the annual total. In this way, we extract seasonal and intraday fluctuation characteristics of renewable energy and load. The actual values imported into the model are those scaled by the full year 2019 totals.

Changes/additions to the manuscript:

(Manuscript, Line 494-497) We pre-processed the raw data for 2018 to find the hourly PV and wind generation and load as a percentage of the total for the year. In this way, we extracted the seasonal and intraday variations that characterize renewables and load, and excluded the influence of trend changes on the results.

Comment 1.3:

Model validation against recent trends. The model starts planning pathways from 2019. Given that actual data for the past five years (2020-2024) may be available for indicators such as energy consumption, CO2 emissions, and renewable energy generation, a key question arises: which modeling pathway best aligns with recent trends? Addressing this question would strengthen the model's empirical credibility and its relevance to real-world transitions.

Response:

Thank you very much for your question. The calibration of the model is the biggest part of the workload in the development of an energy system model or IAM model, and our model is indeed one of the few models that have updated the base year calibration to near 2020 (the vast majority of the IAM models in the comparison are still calibrated to 2015 as their base year). At the same time, the vast majority of IAM models do not report data for every year, but usually on a 5-year cycle. In our model, the paths of all scenarios up to 2030 are aligned with the NDC scenario to reflect the inertia of current

policy. Currently the official published energy balances are only updated to 2022, otherwise we have compared the data related to 2023.

Variable	Unit	Model	Statistic	Diff
CO_2 Emissions (2023)[1]	Mt	11316.52	11605.93	-2.5%
Primary Energy (2022)[2]	PJ	142764.08	143639.29	-0.6%
Final Energy (2022)[2]	PJ	103137.47	99546.38	3.6%
Hydro power generation (2023)[3]	TWh	1324.43	1285.90	3.0%
Nuclear power generation (2023)[3]	TWh	432.09	434.70	-0.6%
Solar power generation (2023)[3]	TWh	587.99	584.20	0.6%
Wind power generation (2023)[3]	TWh	913.38	885.90	3.1%
Hydro power capacity (2023)[3]	GW	421.54	422.37	-0.2%
Nuclear power capacity (2023)[3]	GW	56.91	56.91	0.0%
Solar power capacity (2023)[3]	GW	609.49	610.48	-0.2%
Wind power capacity (2023)[3]	GW	441.34	441.44	0.0%

[1] Global Carbon Budget (2024)

[2] China Energy Statistical Yearbook 2023 – The Energy Balance 2022

[3] Summary of Basic Data on Electricity Statistics 2023

Although our model is not tuned specifically for short-term forecasting, but rather focuses on long-term path planning, we have forecast errors of less than 4% on the carbon, energy and power generation indicators you are interested in, which is a good reflection of the reality of the situation.

Changes/additions to the manuscript:

(Manuscript, Line 418-419) Model outputs for 2020-2023 were calibrated against official statistics.

Comment 1.4:

Parameter sensitivity analysis. The model relies on numerous predefined parameters. To assess robustness, the authors should: (a) Identify key parameters that distinguish this model from others (e.g., demand response elasticity, storage cost assumptions). (b) Conduct sensitivity analyses (e.g., Monte Carlo simulations) to quantify how uncertainties in these parameters affect outcomes (e.g., marginal abatement costs, technology portfolios).

Response:

Thank you very much for your question. Modeling requires a lot of assumptions about future techno-economic parameters. For this reason, we show important cost assumptions in Supplementary Table 5.

We know that sensitivity analysis is very important. However, given the computational scale of the model (10 hours for a single scenario on a 64-core CPU workstation), large-scale sensitivity analysis is difficult at this stage, and we have a dedicated paper for parameter uncertainty (https://www.nature.com/articles/s41467-021-27671-0). Therefore, we performed 1000 rounds of Monte Carlo simulations using the same constraints but without the refined time resolution scenario (i.e., CN60-noTS). We set the uncertainty range for wind power costs, PV costs, nuclear power costs, BECCS costs, energy storage costs, and hydrogen energy costs to be 70%-143% of the intermediate scenario (Log-Normal distribution), and the uncertainty range for wind power installation growth, PV installation growth, nuclear power installation growth, and price elasticity to be 70%-130% of the intermediate scenario (Normal distribution). The cumulative carbon budget for 2020-2100 is 200-250 Gt (uniform distribution). In addition, we set up the On-Time Action Scenario (CN60) and the 2025 Early Action Scenario (CN60E) to represent different policy strengths. The following three figures illustrate the net-zero transition emission trajectories, marginal abatement costs, and power capacity under uncertainty perturbations in the input parameters.

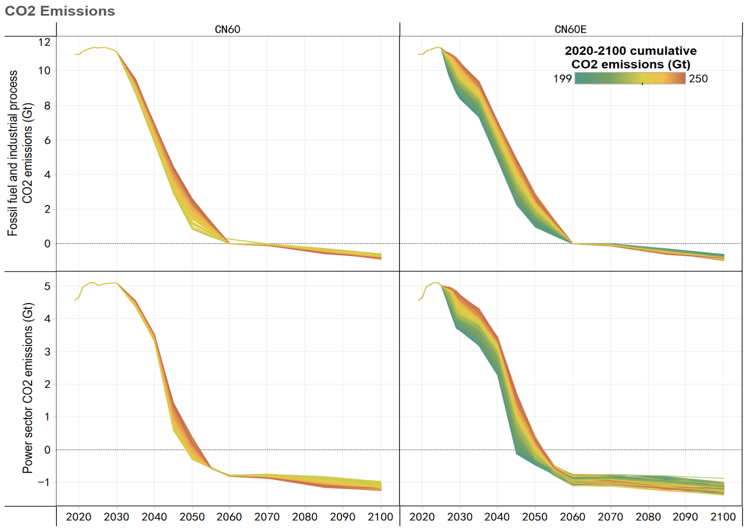

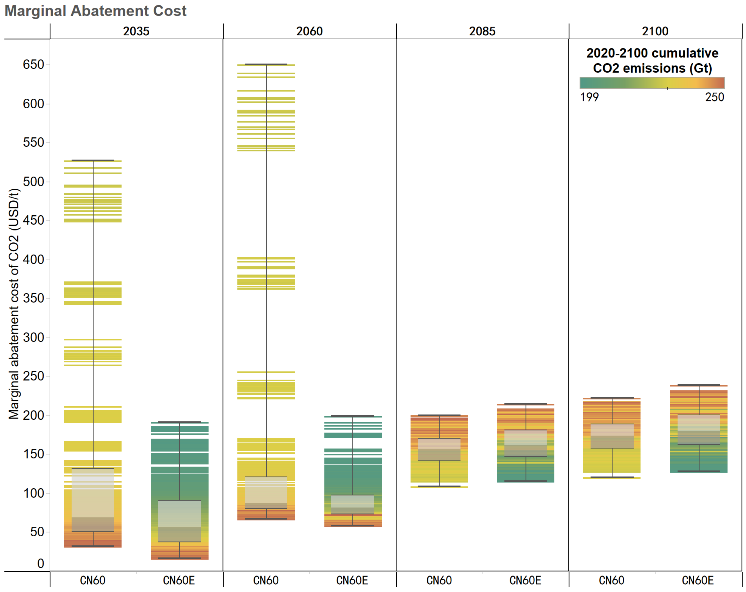

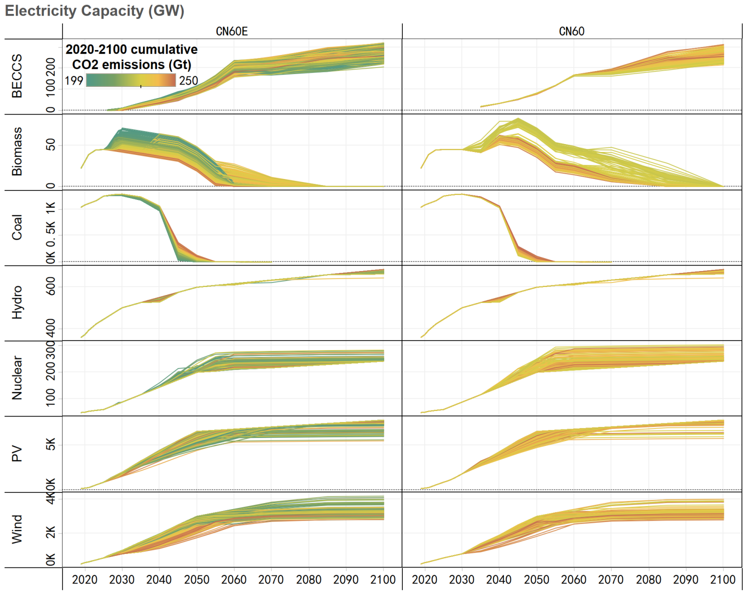

Changes/additions to the manuscript:

(Manuscript, Line 627-628) Main parameter assumptions are listed in the Supplementary Table 5. Source Data are provided with this paper.

(Supplementary Information, Supplementary Table 5) Add table.

Technology	Unit	2025	2030	2060	2100
**Power Plant**					
Coal without CCS	USD/kW	547	545	535	535
Coal with CCS	USD/kW	1031	1000	913	913
Gas without CCS	USD/kW	550	550	550	550
Gas with CCS	USD/kW	904	897	897	897
Nuclear (PWR)	USD/kW	1946	1897	1805	1805

Nuclear (HTGR)	USD/kW	2571	2286	1829	1805
Biomass without CCS	USD/kW	1220	1102	735	735
Biomass with CCS	USD/kW	1916	1726	1136	1136
Hydro	USD/kW	1066	1066	1066	1066
Onshore Wind	USD/kW	602	542	439	439
Offshore Wind	USD/kW	1287	1030	667	667
Solar PV	USD/kW	377	339	247	247
Solar CSP	USD/kW	2531	2405	1782	1371
**Energy Storage**					
Pumped Hydro	USD/kW	908	896	833	754
Battery storage LFP	USD/kWh	119	110	69	37
Battery storage NMC	USD/kWh	126	117	73	39
Hydrogen alkaline	USD/kW	246	232	205	205
Hydrogen PEM	USD/kW	1043	580	213	213
Hydrogen SOEC	USD/kW	1712	1384	387	387
**Demand-Side Response**					
Load time-shifting	USD/kWh·hour	0.04	0.03	0.02	0.02
V2G feed-in cost	USD/kWh	0.08	0.06	0.04	0.04

Supplementary Table 5 Power plant construction, energy storage construction, hydrogen electrolyzer construction and demand side response costs.

Comment 1.5:

5) Page 6. The text cites "Fig. 5d", but Figure 5 does not include a panel labeled "d".

Response:

We are very sorry for the issue with our figure citation, which has been corrected in the revised version of the manuscript.

Changes/additions to the manuscript:

(Manuscript, Line 246) these interactions significantly diminish the reliance on power-based energy storage, such as pumped hydro storage (Fig. 4d).

(Extended Data Fig. 2) Rename to Fig. 4.

Reviewer 2

Comment 2.1:

Whether the model covers all factors and contingencies, response to special circumstances such as extreme weather, policy changes, etc. There is a need for further in-depth analysis of technological development uncertainties, such as the expansion of the scale of renewable energy from the point of view of renewable energy systems, the rate of decline in the cost of renewable energy technologies, and breakthroughs in energy storage technologies.

Response:

Thank you very much for your question. The scenario setting and optimization simulation of our model adopts a multi-layer temporal nested structure, which achieves the simulation coverage of the whole year. Among which the most refined hourly simulation, we selected the load and renewable energy output curves of a typical weekday in summer for 24 consecutive hours, which is used to reflect the daily condition of the energy system operation. In order to ensure a correct estimation of energy supply and demand throughout the year, the current simulations are likelihood scenarios rather than extreme scenarios. Therefore, we are mainly dealing with a “high probability - low risk” scenario for the future development of the energy system. To cope with small probability-high risk situations, we retain a larger margin of safety while satisfying normal operation by adding reserve constraints, credible output capacity constraints, peaking constraints, and start-stop constraints.

In response to policy and technology uncertainty, this is indeed a constant theme in long-term path planning research. We have quantified these factors you mentioned using Monte Carlo analysis in our Nature Communications paper in 2022 (10.1038/s41467-021-27671-0). Due to the heavy computational effort associated with enhanced temporal resolution, it is impractical to perform large-scale scenario simulations in this framework. However, in response to your question, we performed Monte Carlo simulations using the same constraints but without the refined time resolution scenario (i.e., CN60-noTS). We set the uncertainty range for wind power costs, PV costs, nuclear power costs, BECCS costs, energy storage costs, and hydrogen energy costs to be 70%-143% of the intermediate scenario (Log-Normal distribution), and the uncertainty range for wind power installation growth, PV installation growth, nuclear power installation growth, and price elasticity to be 70%-130% of the intermediate scenario (Normal distribution). The cumulative carbon budget for 2020-2100 is 200-250 Gt (uniform distribution). In addition, we set up the On-Time Action Scenario (CN60) and the 2025 Early Action Scenario (CN60E) to

represent different policy strengths. The results show that in 2060, the proportion of renewable energy in the primary energy supply of the energy system is 60-66%, the electrification rate is 56-59%, and the installed capacity of PV and wind power is 5.6-6.8 TW and 2.6-3.5 TW, respectively. More data and result figures can be seen in our response to Comment 1.4. It can be seen that our results remain robust under uncertainty perturbations.

Changes/additions to the manuscript:

(Manuscript, Line 403-404) While richer simulations of uncertainty and extreme scenarios are difficult to perform due to computational capacity limitations, the insights garnered here provide a nuanced understanding of the multifaceted challenges and prospects pertaining to energy system flexibility in China, offering valuable lessons that could be beneficial for other developing nations navigating towards a net-zero future.

Comment 2.2:

Feedback on how the impacts of different policies and market mechanisms on the flexibility of the energy system will feed into forecasts and be accurately predicted.

Response:

Thank you very much for your question. In our modelling, we have carved out policies for energy and climate change that have been released by the end of 2020. For example, we consider no new coal power after 2030, wind and PV capacity exceeding 1,200GW in 2030, and a reduction in carbon intensity of more than 65% in 2030 compared to 2005. These policies directly determine the future direction of energy transition and climate change mitigation.

In order to describe the ongoing coal power “Three Retrofits” plan, our model is dedicated to the technologies of coal power biomass-coal blending retrofit, flexibility retrofit, and CCS retrofit. The model optimizes the transition options for different coal power units, i.e., continued service, early retirement, and retrofit, by considering the technical economics (age, efficiency, cost, etc.). For retrofits, multiple retrofits can be performed simultaneously, or a particular retrofit can be performed separately.

The model aims at cost minimization and does not include subsidies at the level of power system operation. In 2025, the government has eliminated all renewable power subsidies and incentives such as price and quantity guarantees, i.e., all power sources (including thermal and renewable power) are fully traded in the electricity market.

For demand-side response, the current level of subsidy varies from province to province in China, and given that we are a national model, we selected the demand-side response rate for load shifting in Yunnan Province, which has a moderate

demand-side response subsidy, as a basis (1 CNY/kWh for 3-hour load time-shifting), and set the subsidy for load time-shifting to 4 US cents/kWh·hour. Therefore, in model optimization, if the cost reduction due to load time-shifting is higher than the required subsidy, the pair is operated to the extent that it can be time-shifted.

Changes/additions to the manuscript:

(Manuscript, Line 474-479) The model portrays the ongoing CFPP retrofit plan. Flexibility retrofit, CCS retrofit, biomass-coal blending retrofit, and BECCS retrofit can be performed on compatible units. At the same time, units are able to gain better deep regulation capacity through additional investment (500 CNY/kW)[63]. Taking the ultra-supercritical unit as an example, the minimum load level after the retrofit dips from 40% to 20%.

(Manuscript, Line 558-565) Based on the TIMES open-source framework[69,70], the China TIMES 2.0 model models load shifting as a special bi-directional energy storage technology that is able to represent the maximum allowed deviations from nominal demand loads, maximum advance or delay for meeting the shifted loads, and the value per hour shifted by setting the storage capacity, the storage time, and the operating cost, respectively. The model conservatively assumes a subsidy of approximately 4 US cents/kWh per hour of shifting, whether advanced or delayed, based on a subsidy of 1 CNY/kWh for three-hour load shifting in Yunnan Province.

Comment 2.3:

How is the balancing of peak load shifting for V2G with the utilization of renewable energy sources such as PV during daytime peaks considered.

Response:

Thank you very much for your question. In terms of the mechanism of the model, China TIMES model uses a cost minimization optimization approach. During the midday hours, the supply exceeds the demand due to the higher renewable energy output, and thus the marginal cost of electricity for that time period decreases, which guides more demand to be concentrated in this time period.

Taking the transportation sector as an example, we are a priori given two charging time profiles, fast charging and slow charging. For the CN60 scenario, we only consider the shift between fast and slow charging profiles, and for the CN60-LM scenario, we consider load time shifting and V2G. Load time shifting is to shift a portion of the charging demand from other time periods other than noon to the noon time for charging, where there is only a charging process between the EV and the grid, and the SOC of

the batteries is only on the rise. V2G technology, on the other hand, treats the EV as an energy storage technology, which is connected to the grid for both charging and discharging during the day, and the SOC of its battery remains constant after fluctuations.

The difference in the cost of electricity over the course of the day due to the concentration of PV output at noon, which directs the model to seek the least costly way to meet demand, involves both the supply-side derating of other adjustable power sources as well as the demand-side use of demand-side response measures to increase load during that time of day.

Comment 2.4:

The sub-annual time model constructed is divided into: annual-seasonal-weekly-daily-hourly, whether energy flexibility is taken into account for over-seasonalization, and why not annual-monthly-weekly-daily-hourly and consider a more detailed division of time even down to the minute.

Response:

Thank you very much for your question about our time nested structure tradeoffs. In our model, pumped hydro, CAES, and hydrogen storage all function as both intraday and long-duration storage. Undoubtedly, more fine-grained simulations (considering each month as well as taking into account the minute scale) would enable the checking of the short-term/ very-short-term system operational state at more time periods, but this would significantly increase the computational burden of the model. At this stage, our model has about 200 million non-zeroes, and with the application of Gurobi's multi-threaded Barrier method for solving, it takes about 10 hours and about 100 GB of memory, which is close to the bottleneck of the computational power of desktop workstations.

On the other hand, the focus of energy transition and climate change mitigation research is not to provide much insight into power system operational decisions, but rather to improve the feasibility of energy system planning by taking into account operational constraints, and thus to guide “big inertia” energy systems towards no-regrets investments.

At present, our division of seasons takes into account to a large extent the dry and wet seasons of hydropower, the seasonal differences in wind and solar resources, and the seasonal differences in space heating and cooling demand, and basically reflects the characteristics of power supply and demand in different months.

Hourly-level simulations that can reflect major flexibility aspects such as peak-shaving (2h or more), demand side response (1h-3h), thermal power start/stop (4-12 hours), and energy storage operation (1-6 hours). Minute-scale simulation mainly addresses aspects such as frequency regulation, load tracking, ramp control, spike loads, etc., which are often generated due to uncertainty in energy use behavior or natural conditions, and are not mainly dominated by socio-economic development and changes in production mode and lifestyle, etc. For minute-scale simulations, the use of power system-specific models and scenario-generation techniques can provide greater explanatory power for these extreme scenarios.

Changes/additions to the manuscript:

(Manuscript, Line 637-639) The model has about 200 million non-zero values, and applying the Barrier method of the Gurobi 11 software to solve it requires about 10 hours of computation and about 100 GB of memory on a workstation with 64 CPU cores.

Reviewer 3

General Comment:

This paper sets out to improve the temporal resolution of the China TIMES model, arguing that such models typically fail to, due to limited representation, capture the necessary flexibility needed for higher renewable systems. In doing so, the study makes a valuable contribution by enhancing the time-slice granularity of the China-TIMES model, strengthening its capability to represent time-varying electricity load curves, proactive demand response, and the interaction between demand and supply. The authors present comprehensive results illustrating China's energy transition under its net-zero targets, considering system flexibility and financial implications.

However, there is a question as to whether the analysis is novel enough to meet the high bar for acceptance in a journal such as Nature Communications. There are other examples of IAM and energy system models running at higher temporal resolution. Therefore, further development of the argument regarding novelty is required.

Furthermore, the paper needs to bring stronger clarity to the difference between the 'standard' model and improved model. It was not clear from the scenario descriptions as to the key scenarios that were being compared, although this could be partly deciphered from Figure 2. Also, key results need to feature in the abstract – to headline the main findings. Additional information is needed in the main paper (incl. Methods) as to what the 'noTS' scenario means. This is not described; if it is simply a scenario that has no timeslices (TS) at all then this is less interesting, but if it was the previous version of China TIMES that had some more aggregated TS then that would be a useful comparison.

With the model effectively demonstrating the role of transportation time-shifting load and the potential of various short- and long-term storage technologies in electricity supply - both of which are critical components of your results - it is necessary to understand the prior baseline before the improvements were made.

There is also the question of scenario design. While the study constructs three net-zero scenarios with varying levels of demand-side participation, the overarching net-zero constraints seem to dominate the energy transition dynamics, thereby weakening the impact of demand-side management assumptions. As a result, the authors should clarify their scenario assumptions or revise the scenario design to align more closely with this granular modelling approach. This would help better illustrate the marginal impacts of the sub-annual analysis on the national net-zero transition.

In general, some of the language could be tightened up, and would benefit from further

proof reading.

Response:

Thank you very much for your constructive comments. First of all, the main novelty of this paper is the significant extension of the sub-annual (up to hourly) modeling of the IAMs/energy system model, especially in the portrayal of the demand side, which overcomes the problem of exogenous load curves in previous studies and enables the integrated simulation of power dispatch, load characteristic changes, and demand response measures in the same model. More responses about new insights and improvements to our model can be found in the subsequent response to Comment 3.8. In our response to Comment 3.18, we also provided recommendations for policymakers and the modeling community based on the results.

Second, you mentioned the missing distinction between sub-annual models and the original models without sub-annual modeling. And there is a lack of key take-away findings. For this reason, we have revised the summary in our response to Comment 3.1 to include quantitative analyses for renewable energy, thermal power, dispatchable resources, and demand-side response. In this study, CN60-noTS, relaxes all sub-annual constraints and assumes that all supply and demand are equally distributed throughout the year. To address the issue of model differences, we present the three sub-annual scenarios (CN60-noLM, CN60-LM, and CN60) and the annual scenario (CN60-noTS) differences at the beginning of the results section, and we show the results for the CN60-noTS scenario in Fig. 1 and Supplementary Fig. 13, which are detailed in the responses to the Comment 3.6, 3.7 and 3.17.

Third, you mentioned the baseline regarding energy storage and demand-side response. Modeling with the goal of cost minimization does not result in significant activity and investment in energy storage in scenarios that include only annual-level constraints. The capacity and investment in CN60-noTS regarding energy storage is due to the consideration of the current policy renewable energy mandate to equip storage (10% of installed renewable energy) and the planned construction capacity of pumped hydro. For details, see the response to Comment 3.10. It is not difficult to see a significant rise in demand for energy storage in the scenarios that consider sub-annual balancing. Demand side response mainly responds to peak loads and therefore its main operating dynamics cannot be portrayed in models that do not consider sub-annual level modeling or even hourly level modeling.

Fourth, you provided suggestions for our scenario design. As you note, the net-zero constraint and the carbon budget assumptions have a strong impact on the model results, which is to be expected. However, how net zero is achieved is something that

needs to be carefully assessed. By setting up three scenarios with different levels of demand-side participation, we show how the energy system, especially electricity, hydrogen, energy storage and demand behavior may change even with the same climate goals. The importance of improving the temporal resolution during the modeling of net-zero transition pathways is also reinforced by the comparison with scenarios that do not take into account sub-annual level modeling. To this end, we have further elaborated on the scenario setting and inter-scenario comparisons in both the main text and the methodology section, as detailed in the responses to Comment 3.5, 3.21, 3.22, 3.23.

Finally, we have carefully proofread the entire text in response to your questions to minimize ambiguity, opacity, and categorical statements, and some of the changes can be found in the responses to Comment 3.2, 3.3, 3.4, 3.9, 3.11, 3.12, 3.13, 3.14, 3.15, 3.17, 3.19, 3.24, 3.25, and 3.26.

We hope that our revisions will help you and the readers to better discover the contribution of this study to China's net-zero transition and modeling community.

Comment 3.1:

Abstract: There is a need for some headline figures in here that underpin the main arguments about improved temporal resolution. What would you consider to be the main findings in quantitative terms?

Response:

Thank you very much for your advice. We have included quantitative data on renewable energy, thermal power, dispatchable resources, and demand side management measures in the revised abstract.

Changes/additions to the manuscript:

(Manuscript, Line 21-27) China-based case study finds that, compared to the annual-level mitigation scenario, the renewables share would decrease by 4% by 2060, while thermal power capacity would increase by 18%; dispatchable resources (energy storage, grid-connected hydrogen and electric vehicles) would grow faster. Incentives for demand-side response such as load time-shifting and vehicle-to-grid can reduce investment in pumped hydro by 23% and yield more than a threefold cost-benefit ratio.

Comment 3.2:

Line 37. Define what you mean by 'IAM'. Many modellers would view China TIMES as an integrated energy systems model, not an IAM.

Response:

Thank you very much for bringing this up. It is true that there are different understandings of the definition of an IAM model, especially for national IAMs, which often do not directly integrate their results with climate models in the same way as global IAMs. Your suggestion for adding an IAM definition is apt, and it allows the reader to get a more intuitive sense of the modeling improvements presented in this paper.

Additionally, according to the definition of IAMC, the China TIMES model belongs to the category of process-based optimization models at the national level, which focuses on the energy system and also involves the quantitative description of the interactions between energy, water, land, air quality and other domains. For research on the energy-food-water-air quality connection, you can follow our paper in Nature Sustainability (10.1038/s41893-024-01400-z).

Changes/additions to the manuscript:

(Manuscript, Line 45-47) Integrated Assessment Models (IAMs) are designed to provide a quantitative description of key human and earth system processes and their interactions, which usually includes a portrayal of energy, land, water, climate, and other systems.

(Manuscript, Line 413-414) With modeling capabilities for water system, land system and air quality, China TIMES 2.0 is becoming a representative national IAM for China.

Comment 3.3:

Line 41-42. Why does this result in ‘casting doubts’?

Response:

Thank you very much for your question. In China, the current LCOE of wind power and PV with storage is already lower than the LCOE of coal power, which means coal power can't compete with renewable energy in terms of economy in the dimension of electricity generation. Therefore, if models do not have the capacity to simulation at the sub-annual level, the role of coal power for grid integration, peaking shelving, capacity reserve, etc., cannot be fully charactered. Thus, without considering the role of coal power for flexibility, coal power is deemed to be cost-effective to exit quickly in the near term in the face of carbon reduction pressures. Disorganized renewable energy expansion and coal power exit have already forced time-sharing blackouts in some Chinese provinces in 2022. As a result, a transition pathway that does not take

into account power system flexibility has been recognized by policymakers as having a feasibility concern.

Changes/additions to the manuscript:

(Manuscript, Line 52-53) Without considering the challenges of power system flexibility, such pathways casts doubt on the practicability of their proposed transition pathways

---

Comment 3.4:

Line 64-65. You state that “Our findings indicate that current IAM model does not capture well the challenges and opportunities of accessing large-scale VRE and electrical appliances.” What ‘model’ are you referring to – China TIMES or IAMs in general? Is this context to your research rather than your finding?

Response:

Thank you very much for pointing out where our language is not rigorous enough, we will try to tighten up the description. IAM modeling here refers to most current IAMs or energy system models on an annual scale.

Changes/additions to the manuscript:

(Manuscript, Line 75-76) Most IAMs or energy models are typically modeled on an annual scale, making it difficult to capture well the challenges and opportunities of accessing large-scale VRE and electrical appliances.

Comment 3.5:

Line 66-71. The energy demand temporal disaggregation proposed in this paper does not seem to directly address the obstacles mentioned - "fragmented regulatory frameworks, limited economic incentives, and insufficient market mechanisms.” Could you clarify what the proactive demand response strategies refer to in your scenarios or modelling? Additionally, what policy recommendations can be derived from this analysis?

Response:

Thank you very much for your question, the sentence you quoted we recognize as forming the context of the flexibility challenge, not our findings, and we have adjusted it to the first paragraph of the introduction.

The proactive demand response stands for proactive measures that provide

specialized incentives such as load time shifting, V2G, etc., and corresponds to the relevant technologies in the CN60-LM scenario later. We will refine the text to make our intentions clearer.

Two policy insights can be gleaned from this study.1) Orderly energy consumption (especially EV and hydrogen production demand), energy storage, and power dispatch will be important in the net-zero transition, and this paper quantifies their size and operating modes.2) Demand-side response measures, such as V2G, load time-shifting, which are highly cost-effective for peaking loads, should be incentivized.

Changes/additions to the manuscript:

(Manuscript, Line 39-42) Fragmented regulatory frameworks, limited economic incentives and insufficient market mechanisms pose significant obstacles to the redistribution of load in a manner that aligns with power output, consequently constraining system flexibility.

(Manuscript, Line 78-85) Our findings indicate that effective coordination between orderly energy consumption, energy storage and power dispatching are pivotal in rectifying demand-supply imbalances. Rapidly rising electricity demand for EV charging and green hydrogen production should be directed to accommodate fluctuations in renewable energy generation. Proactive demand response strategies such as V2G and load time-shifting, can significantly curtail peak load and alleviate ramping issues, thereby circumventing the need for energy storage investment, and preventing volatile price oscillations. This signifies the key in subsidizing demand-side response measures.

Comment 3.6:

Line 76-81. Despite some description in Methods, more elaboration is needed here prior to reading the results. This includes for ‘noTS’, which is not described. What does ‘orderly energy consumption’ mean?

Response:

Thank you very much for your advice. We have added explanations to CN60 and CN60-LM indicating the most important characteristics of the changes. Where CN60 ordered energy demand mainly means that hydrogen production can be regulated over time and EV charging can freely choose between fast and slow charging modes (but not load time shifting); CN60-LM considers demand-side response measures such as load time shifting for almost all energy service demands and adds V2G technology carve-outs.

For the CN60-noTS scenario, we have also enriched the description by stating the main sub-annual constraints and technical carve-outs that we remove.

Changes/additions to the manuscript:

(Manuscript, Line 88-99) In this study, we propose four sub-annual scenarios: NDC, CN60, CN60-noLM, and CN60-LM, which represent the baseline reference, the net-zero scenario with orderly energy consumption (flexible load adjustment for transportation and hydrogen production), the net-zero scenario maintaining current energy consumption patterns and the net-zero scenario with proactive supply-demand interactions (introduction of demand-side response for almost all energy service demands and V2G technology), respectively. For comparison, we also present a scenario without sub-annual constraints, CN60-noTS, which removes the seasonal, weekly, and daily variations in renewable energy output, load carve-outs (assuming an average distribution) and relaxes the power plant flexibility constraints. Energy storage and demand-side response measures are also not considered. Such a scenario is equivalent to the scenario of a traditional annual-level IAM model without embedded sub-annual level inscriptions.

Comment 3.7:

Figure 1 would benefit from including the 'noTS' scenario for completeness - although perhaps may not show key differences due to the results being presented at an annual level.

Response:

Thank you very much for your suggestion, we have added the CN60-noTS scenario in Figure 1.

Changes/additions to the manuscript:

(Manuscript, Figure 1) modify figure.

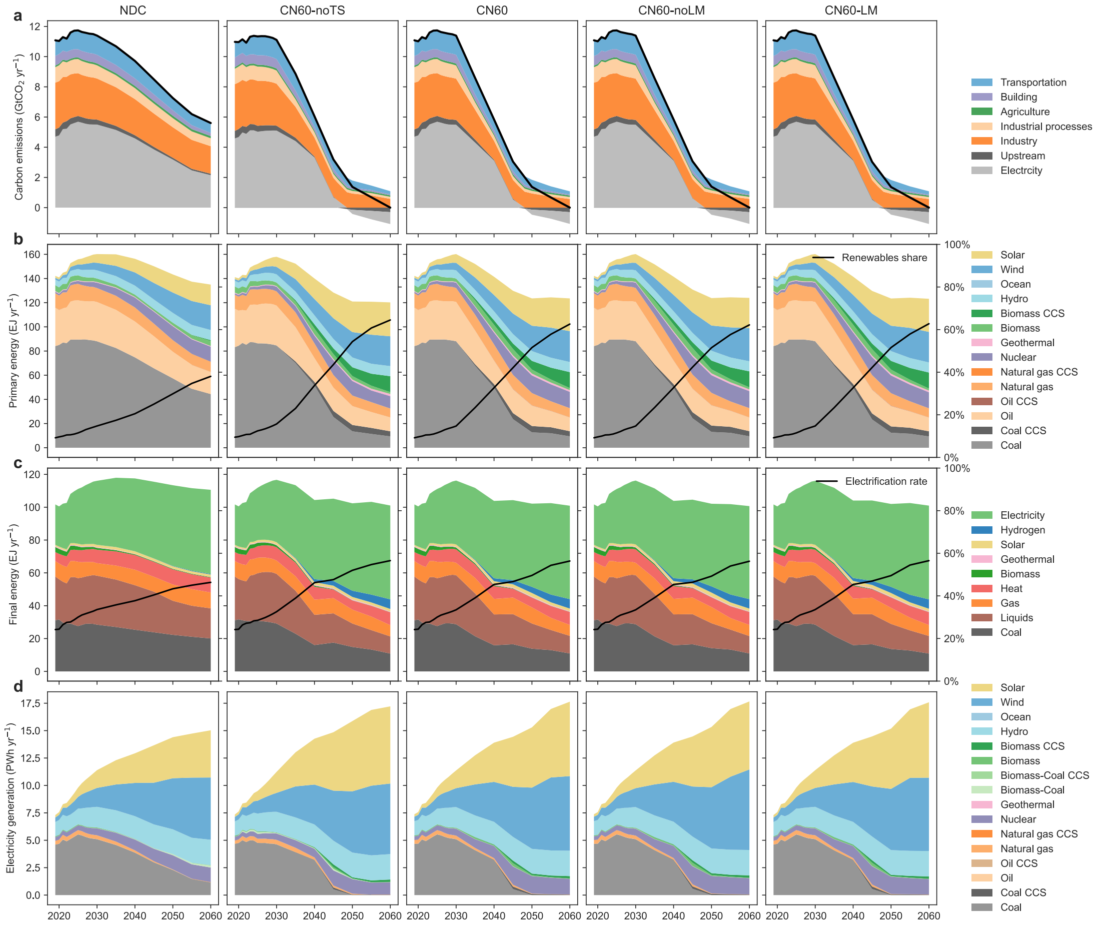

Comment 3.8:

Line 82-95. Most modelling results presented here, along with the corresponding figures in your supplementary document, indicate no pronounced differences among your three CN60 scenarios. As I understand, China's key energy transition trends—such as the emissions peak, energy consumption peak, and declining trajectory—are primarily determined by your net-zero constraints or carbon budget constraints. Could you clarify how your different demand- and supply-side strategies (key modelling assumptions) applied in your scenarios influence China's net-zero transition pathway?

Additionally, what new insights does your modelling improvement provide for China net zero transition?

As shown in Figure 2, there is a significant difference between sub-annual and annual modelling analyses across the aspects you mentioned. However, this impact remains generally consistent across the three CN60 scenarios (e.g., sectoral electricity demand). As I understand, these variations cannot be attributed to the effects of more granular and flexible demand-side management on the net-zero transition. Instead,

they likely reflect structural uncertainties in this TIMES modelling results. Therefore, I am not convinced that this sub-annual analysis demonstrates any substantial impact on the energy transition itself. Additionally, in your comparison with other modelling analyses, I recommend clarifying the key differences in scenario assumptions between your analysis and other harmonized scenarios - particularly regarding demand-side input assumptions, which may contribute more significantly to the differences you present than the variations in time slices.

Response:

Thank you very much for your enlightening comments. Overall, we will respond to your comments in three ways: a comparison of the impacts of the sub-annual model scenarios and the annual model scenarios on the transition paths; a comparison between the three sub-annual scenarios with varying degrees of demand-side participation; and a comparison of the assumptions of the models proposed in this study with those of other comparative models.

1. First of all, as you said, the most critical influences on the net-zero transition pathway are the carbon budget and the net-zero target. Within a given carbon constraint, we, model both supply and demand sub-annually to address the feasibility concerns arising from the current change in energy system landscape under the rapid development of renewable energy in China.

For the supply side, **1) we consider the output curves of renewables, the regulation performance of thermal, hydro and nuclear power, which changes the choice of model optimization.** In the model without sub-annual modeling, since PV already has a clear cost advantage, without additional constraints, almost all future electricity demand will shift to be supplied by PV, which is clearly a large gap with reality. **2) We consider diverse energy storage needs and achieve endogenous optimization of the model for energy storage capacity expansion and operation simulation.** Without sub-annual modeling, energy storage will not be applied based on cost considerations, and most energy system models or IAMs currently use a given proportion of renewable energy with storage, while such an exogenously given coefficient is still questionable; 3**) We consider the coordination between green hydrogen production and the power system.** When sub-annual modeling is not considered, the green hydrogen cost are annual average cost, which are significantly higher than the cost using midday affluent power. Also. orderly green hydrogen production is an important complement to system flexibility, an operational state that is not captured in annual-scale models.

For the demand side, **1) we consider the real-time matching of supply and demand**

on a sub-annual basis. When sub-annual modeling is not considered, supply-demand matching is built on an annual scale, thus ignoring many sub-annual mismatches that pose feasibility concerns; **2) we consider the temporal distribution for each type of energy service demand and endogenously generate load curves at different periods in the future.** Existing power system-specific models' future load profiles are often held constant or exogenously given, but changes in the load curves with electricity substitution and behavioral changes can have impacts on the pathways; **3) We consider demand-side response measures such as demand curtailment and demand shifting.** The costs and benefits of demand-side response are portrayed by making full use of multi-timeslice modeling of elastic demand, load time-shift, and V2G, and setting appropriate subsidies according to the real situation.

Through sub-annual modeling, we were able to obtain some special results, some examples are as follows: the shift of thermal power from providing power to providing flexibility rather than a quick exit provides a path for a smooth transition of thermal power; for renewables, hydropower and wind power received more attention; and for energy storage, we identified a clear correlation between the pumped hydro capacity and the level of demand-side participation, whereas the battery storage capacity is mainly associated with the PV and wind power capacities are correlated. Charging modes will tend to favor the midday fast charging option to take advantage of daytime solar energy.

2. Secondly, as you say, if you look at electricity consumption by sector, there is little difference between the three sub-annual scenarios. This is due to the need to meet exogenously given energy services demand regardless of the level of demand side participation. But looking at the three scenarios' dispatchable resource, wind and PV ratios, and energy storage investments, you can see significant differences. This also illustrates the cross-sectoral impact of demand-side measures, making a significant difference to the transformation of the electricity sector in particular. Thus, the differences in the power sector expansion and operating modes, investments, and transition burdens required to meet demand in the three scenarios that essentially fix the end-use sector transition trend and carbon emissions are precisely what demand-side participation creates.

3. Since the ENGAGE project does not harmonize the demand inputs for each model group, the demand input assumptions used by each model may vary. We reviewed the raw data submitted to the database, and no fewer than three models reported data in GDP, population, cement production, steel production, and floor space of the residential and commercial houses. See the figure below for details.

For socio-economic parameters, China TIMES model considers China's policy goal of

doubling its GDP in 2035 compared to 2020, and sets an average annual growth rate of 3% after 2040. Our model takes into account the fact that China is already in a trend of population decline, which is slightly less overall than the other models. Calculations show that the main driver of demand, GDP per capita, will be on the high side of all models, reaching the policy target of $30,000 per person in 2050.

There are large differences in the base year due to the different sources of calibration data for the different models, but in terms of trends, the China TIMES model is basically in the middle of the models in terms of indicators such as iron and steel, cement, and floor space of the residential and commercial houses.

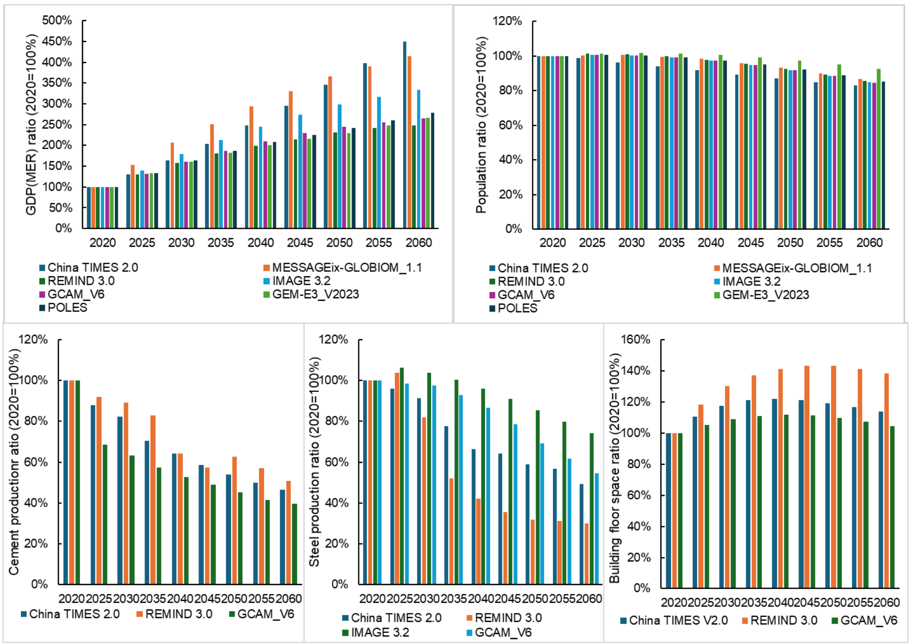

As can be seen in Figure 2, there are some uncertainties between the different models, where the main differences between China TIMES and the other models are reflected in the emission reduction pathways of the power sector, thermal capacity, and energy storage ratio. These items show significant differences in trends that can be explained not only by the size of demand, but due to the sub-annual level modeling that exploits the easy-to-overlook value of thermal power. Hydrogen energy, on the other hand, enters an accelerated period of development after 2040, with electrification rates showing a clear bottleneck after 2040, because power balance becomes more difficult.

Changes/additions to the manuscript:

(Manuscript, Line 143-145) Thermal power achieves a shift from electricity provider to flexibility provider, mitigating the possible shocks of rapid decommissioning, which is unique to the sub-annual perspective.

(Manuscript, Line 147-151) Comparing the three sub-annual scenarios, the demand-

side measures have cross-sectoral impacts, in particular significant impacts on power transformation and energy storage development. Energy storage is an important source of system flexibility, both for frequency and voltage support as well as for energy transfer. Energy storage is an important source of system flexibility, both for frequency and voltage support as well as for energy transfer.

(Manuscript, Line 175-176) Hydropower has gained significantly in importance in the sub-annual perspective and is poised to become a central pillar of flexibility in the power supply side.

(Manuscript, Line 205-206) Benefiting from cost differences across time due to sub-annual modeling, under the CN60 scenario, EV would shift from charging primarily at night to charging during the day, thereby utilizing PV power.

(Manuscript, Line 608-624) For comparison, we introduced six prominent global IAMs. China TIMES and these six IAMs worked together on a simulation of the Glasgow+ scenario, guided by the ENGAGE project proposal. This task aims at exploring the consistency of mid-century strategies and policies with the overall objectives of the Paris Agreement. The protocol integrates national and global pathways into a coherent set of low-carbon mid-century strategies, assessing national mid-century strategies and their consistency with global pathways to 1.5/2°C warming levels and identifying the most effective policies in different countries and sectors. The proposal does not mandate harmonization of input assumptions across model groups due to differences in calibration data sources for national and global models. Based on the model input information voluntarily reported by the teams, China TIMES model is in the middle of the models in terms of the demand assumptions for steel, cement, and residential and commercial floor space. The GDP assumptions are on the high side of the models, and the population assumptions are on the low side of the models. Despite the differences in demand assumptions, for the trajectory trends and quantity differences in power sector emissions, storage, and thermal power identified by the comparisons in this paper, they can be accounted for by the implications of sub-annual level modeling.

Comment 3.9:

Line 108-124. Can you be a bit more precise as to the scenarios that are being compared in this section?

Response:

Thank you very much for your advice. We have added the ENGAGE project protocol on scenario design in the text. The detailed net-zero target year for each country is

showed in Supplementary Table 4.

Changes/additions to the manuscript:

(Manuscript, Line 119-127) China TIMES and global IAMs ran the Glasgow+ scenario under the ENGAGE project protocol to extend the net-zero target to all countries based on income level, after considering countries already proposing a net-zero target (Supplementary Table 4). All models set the same carbon budget constraints according to the equal cumulative per capita method, which ensures that the outputs are comparable.

(Supplementary Information, Supplementary Table 4) Add table.

**Region**	**Countries**	**Net-zero year**
Eastern Africa	Burundi, Comoros, Djibouti, Eritrea, Ethiopia, Kenya, Madagascar, Mauritius, Reunion, Rwanda, Sudan, Somalia, Uganda	2070
Northern Africa	Algeria, Egypt, Western Sahara, Libya, Morocco, Tunisia	2045
Southern Africa	Angola, Botswana, Lesotho, Mozambique, Malawi, Namibia, Swaziland, Tanzania, Zambia, Zimbabwe	2070
Western Africa	Benin, Burkina Faso, Central African Republic, Cote d'Ivoire, Cameroon, Democratic Republic of the Congo, Congo, Cape Verde, Gabon, Ghana, Guinea, Gambia, Guinea-Bissau, Equatorial Guinea, Liberia, Mali, Mauritania, Niger, Nigeria, Senegal, Sierra Leone, Sao Tome and Principe, Chad, Togo	2060
Argentina	Argentina	2040
Australia/NZ	Australia, New Zeeland	2040
Brazil	Brazil	2040
Canada	Canada	2040

Central America and the Caribbean	Aruba, Anguilla, Netherlands Antilles, Antigua & Barbuda, Bahamas, Belize, Bermuda, Barbados, Costa Rica, Cuba, Cayman Islands, Dominica, Dominican Republic, Guadeloupe, Grenada, Guatemala, Honduras, Haiti, Jamaica, Saint Kitts and Nevis, Saint Lucia, Montserrat, Martinique, Nicaragua, Panama, El Salvador, Trinidad and Tobago, Saint Vincent and the Grenadines	2045
Central Asia	Armenia, Azerbaijan, Georgia, Kazakhstan, Kyrgyzstan, Mongolia, Tajikistan, Turkmenistan, Uzbekistan	2045
China	China	2060
Colombia	Colombia	2040
EU-12	Bulgaria, Cyprus, Czech Republic, Estonia, Hungary, Lithuania, Latvia, Malta, Poland, Romania, Slovakia, Slovenia	2040
EU-15	Andorra, Austria, Belgium, Denmark, Finland, France, Germany, Greece, Greenland, Ireland, Italy, Luxembourg, Monaco, Netherlands, Portugal, Sweden, Spain, United Kingdom	2040
Eastern Europe	Belarus, Moldova, Ukraine	2050
European Free Trade Association	Iceland, Norway, Switzerland	2040
Europe Non-EU	Albania, Bosnia and Herzegovina, Croatia, Macedonia, Montenegro, Serbia, Turkey	2053
India	India	2060
Indonesia	Indonesia	2050
Japan	Japan	2040
Mexico	Mexico	2040
Middle East	United Arab Emirates, Bahrain, Iran, Iraq, Israel, Jordan, Kuwait, Lebanon, Oman, Palestine, Qatar, Saudi Arabia, Syria, Yemen	2040
Pakistan	Pakistan	2070
Russia	Russia	2060

South Africa	South Africa	2050
Northern South America	French Guiana, Guyana, Suriname, Venezuela	2050
Southern South America	Bolivia, Chile, Ecuador, Peru, Paraguay, Uruguay	2040
South Asia	Afghanistan, Bangladesh, Bhutan, Sri Lanka, Maldives, Nepal	2065
Southeast Asia	American Samoa, Brunei Darussalam, Cocos (Keeling) Islands, Cook Islands, Christmas Island, Fiji, Federated States of Micronesia, Guam, Cambodia, Kiribati, Lao Peoples Democratic Republic, Marshall Islands, Myanmar, Northern Mariana Islands, Malaysia, Mayotte, New Caledonia, Norfolk Island, Niue, Nauru, Pacific Islands Trust Territory, Pitcairn Islands, Philippines, Palau, Papua New Guinea, Democratic People's Republic of Korea, French Polynesia, Singapore, Solomon Islands, Seychelles, Thailand, Tokelau, Timor Leste, Tonga, Tuvalu, Viet Nam, Vanuatu, Samoa	2050
South Korea	South Korea	2050
Taiwan	Taiwan	2040
USA	United States of America	2050

Supplementary Table 4 Net-zero CO_2 target year based on official submissions, aggregated to the GCAM regions, for Glasgow+ scenario.

Comment 3.10:

Line 136-148. As shown in Figure 3c and 3d, the role of nuclear and hydropower storage shows generally consistent patterns in both NDC scenario and net zero scenarios. You then mention "However, cost and security considerations still limit its contribution to flexibility". Could you clarify if you impose any constraints on the power sector, or if it is the model choice based you carbon budget constraints? If it is, I would recommend showing your fundamental assumptions around the power sector in the SI.

Response:

Thank you very much for your question. In order to make the results of the optimization model realistic and policy-guiding, we have set some upper limit and growth rate constraints on capacity expansion in the power sector based on resource endowment, please refer to Supplementary Table 2.

For the nuclear power you mentioned, we have considered 2 major types of nuclear power: for pressurized water reactor (PWR) type, it is only allowed to be built along the coast of China, given the security considerations; for Generation IV nuclear power technology (such as high-temperature gas-cooled reactors (HTGR)), it is allowed to be built inland. According to previous study (10.1016/j.accre.2018.05.002) coastal and inland nuclear capacity is capped at 218GW and 273GW. Since Generation IV nuclear power technology is significantly more expensive than PWR, it is less likely to be used until 2050, given the cost considerations.

For the pumped hydro you mentioned, we follow the target of the Medium and Long Term Development Plan for Pumped Hydro (2021-2035) issued by the National Energy Administration (NEA), and we set the lower limit of pumped hydro at 60GW and 120GW in 2025 and 2030, and according to the current medium and long term planning layout and potential assessment, we set the upper limit at 420GW in 2060, and the upper limit at 500GW in 2100.

Changes/additions to the manuscript:

(Manuscript, Line 507-508) The constraints on the capacity of the power plant are described in Supplementary Table 2.

(Manuscript, Line 534-538) China TIMES 2.0 considers the current policy and sets a lower limit for energy storage development (10% of installed renewable energy) and sets a lower limit for future pumped hydro capacity in accordance with the Medium- and Long-Term Development Plan for Pumped Hydro (2021-2035).

(Supplementary Information, Supplementary Table 2) Add table.

Technology	Unit	2025	2030	2060	2100
Coal power plant capacity upper limit	GW	1300	1350	1350	1350
Nuclear power capacity upper limit	GW	200	200	350	400
Hydropower capacity upper limit	GW	445	500	615	700
Wind power capacity lower limit	GW	600	1000	/	/
Wind power capacity upper limit	GW	/	/	3500	5500
Solar power capacity lower limit	GW	1000	2000	/	/
Solar power capacity upper limit	GW	/	/	6800	8000
Pumped hydro capacity lower limit	GW	60	120	/	/
Pumped hydro capacity upper limit	GW	/	/	420	500

Supplementary Table 2 Power capacity limit assumptions.

The coal power capacity upper limit is set based on existing coal power construction projects from Global Energy Monitor database, assuming that no new coal power units will be built after 2030 according to the policy requirement; it is assumed that only coastal nuclear power construction will be permitted before 2030, and inland nuclear power (only Generation IV reactors) will be allowed to be built according to the 14th Five-year Development Plan; The upper limit for wind and solar capacity is based on the National Energy Administration(NEA)'s guidance of 200 GW of PV and 80 GW of wind per year in the short term, and on the results of the wind resource assessment in the long term. we set the lower limit of pumped hydro at 60GW and 120GW in 2025 and 2030 according to the Pumped Hydro Plan issued by NEA, and according to the long-term planning layout and potential assessment reported by NEA, we set the upper limit at 420GW in 2060, and the upper limit at 500GW in 2100.

Comment 3.11:

Line 141. Can you evidence the statement – 'its flexible operation has gained momentum in both research and policy perspectives'? What does this mean?

Response:

Thank you for your question. The issue of flexibility for nuclear power has been studied and promoted in academia, industry, and by policymakers. In terms of academic research, the article published in Nature Energy considers the techno-economics of flexible nuclear power and its adaptability to renewable power systems (10.1038/s41560-022-00979-x). National Development and Reform Commission of China and National Energy Administration issued guidance in January 2024 (Ref. 35) stating that it "explores nuclear power peaking shaving and studies the feasibility of safe participation of nuclear power in power system regulation." According to our field research, the current nuclear power plant in Jiangsu province has already been performing regular output reduction regulation during the midday hours in summer.

Changes/additions to the manuscript:

(Manuscript, Line 171-174) Nuclear power, traditionally employed to meet baseload, but its flexible operation has gained momentum in both research[34] and policy[35] perspectives. Some nuclear power is already undergoing regular power regulation in the midday of summer.

Comment 3.12:

Line 169-170. Can you state what you are comparing to when discussing the EV

charging shift?

Response:

Thank you very much for your question, the issue of EV behavior is indeed receiving more attention at the moment. We mentioned in “Future load profile generation” of the Method section that we refer to the New Energy Vehicle Industry Development Plan (2021-2035) for the EV charging time distribution, and we showed the details in Supplementary Table 3. We further explain in the main text that we carved out two types of charging modes, and corresponded to intensive charging during the day, and slow charging at night.

Changes/additions to the manuscript:

(Manuscript, Line 203-205) Fast and slow charging devices are modeled according to the EV development plan, with charging taking place at midday and night, respectively (Supplementary Table 3).

(Supplementary Information, Supplementary Table 3) Add table.

Demand	H1	H2	H3	H4	H5	H6	H7	H8	H9	H10	H11	H12	H13	H14	H15	H16	H17	H18	H19	H20	H21	H22	H23	H24
**Commercial**	**2.4%**	**2.4%**	**2.4%**	**2.4%**	**2.4%**	**2.6%**	**3.1%**	**3.9%**	**5.1%**	**5.7%**	**6.0%**	**5.5%**	**5.4%**	**5.8%**	**5.7%**	**5.7%**	**5.9%**	**5.7%**	**4.9%**	**4.6%**	**4.3%**	**3.2%**	**2.8%**	**2.5%**
Cooling	0.8%	0.8%	0.8%	0.8%	0.8%	0.8%	0.8%	0.8%	6.0%	6.0%	6.0%	6.0%	6.8%	6.8%	6.8%	6.8%	6.8%	6.8%	6.8%	6.8%	6.8%	3.0%	3.0%	1.7%
Cooking	2.1%	2.1%	2.1%	2.1%	2.1%	4.8%	6.5%	4.8%	3.4%	4.8%	8.3%	9.2%	6.5%	4.8%	2.5%	3.0%	6.1%	8.3%	4.8%	3.0%	2.5%	2.1%	2.1%	2.1%
Heating	3.3%	3.3%	3.3%	3.3%	3.3%	3.3%	5.3%	5.3%	4.5%	4.5%	4.5%	4.5%	4.5%	4.5%	4.5%	4.5%	4.5%	4.5%	4.5%	5.3%	5.3%	3.3%	3.3%	3.3%
Lighting	0.6%	0.6%	0.6%	0.6%	0.6%	0.6%	1.4%	4.8%	7.5%	7.5%	7.5%	6.7%	6.7%	7.5%	7.7%	7.7%	8.4%	5.6%	6.0%	4.4%	4.4%	1.4%	0.6%	0.6%
Data Center	4.2%	4.2%	4.2%	4.2%	4.2%	4.2%	4.2%	4.2%	4.2%	4.2%	4.2%	4.2%	4.2%	4.2%	4.2%	4.2%	4.2%	4.2%	4.2%	4.2%	4.2%	4.2%	4.2%	4.2%
Others	1.8%	1.8%	1.8%	1.8%	1.8%	1.8%	2.0%	3.8%	5.5%	6.9%	7.0%	5.4%	5.7%	6.8%	6.9%	6.9%	6.5%	6.3%	4.6%	4.3%	3.6%	3.3%	2.2%	1.8%
Hot water	3.6%	3.5%	3.5%	3.5%	3.6%	3.7%	3.8%	3.9%	4.0%	4.1%	4.3%	4.4%	4.6%	4.7%	4.8%	4.9%	4.9%	4.8%	4.7%	4.6%	4.5%	4.2%	3.9%	3.7%
**Rural**	**3.0%**	**3.0%**	**3.0%**	**3.0%**	**3.2%**	**4.6%**	**5.0%**	**4.0%**	**3.5%**	**3.7%**	**4.4%**	**4.4%**	**4.1%**	**4.2%**	**4.1%**	**4.2%**	**4.9%**	**5.6%**	**5.5%**	**5.8%**	**5.5%**	**4.3%**	**3.8%**	**3.3%**
Heating	2.5%	2.5%	2.5%	2.5%	2.5%	2.5%	2.5%	2.5%	3.9%	3.9%	3.9%	3.9%	3.9%	6.8%	6.8%	6.8%	6.8%	6.8%	6.8%	6.8%	3.9%	3.9%	2.5%	2.5%
Cooling	2.1%	2.1%	2.1%	2.1%	2.1%	4.8%	6.5%	4.8%	2.5%	3.9%	9.2%	7.4%	4.8%	3.4%	2.5%	3.0%	7.4%	10.1%	7.9%	2.5%	2.5%	2.1%	2.1%	2.1%
Hot water	4.9%	4.9%	4.9%	4.9%	4.9%	4.9%	3.3%	3.3%	3.3%	3.3%	3.3%	3.3%	3.3%	3.3%	3.3%	3.3%	3.3%	4.9%	4.9%	4.9%	4.9%	4.9%	4.9%	4.9%
Refrigeration	4.2%	4.2%	4.2%	4.2%	4.2%	4.2%	4.2%	4.2%	4.2%	4.2%	4.2%	4.2%	4.2%	4.2%	4.2%	4.2%	4.2%	4.2%	4.2%	4.2%	4.2%	4.2%	4.2%	4.2%
Lighting	0.7%	0.7%	0.7%	0.7%	1.7%	9.9%	10.4%	3.4%	1.9%	1.4%	1.4%	1.4%	1.4%	1.4%	1.4%	1.4%	2.6%	4.4%	6.6%	14.9%	15.4%	7.7%	5.7%	2.7%
Cooking	2.7%	2.7%	2.9%	3.1%	3.5%	4.0%	5.7%	5.5%	3.5%	3.7%	3.5%	5.7%	5.3%	3.9%	3.6%	3.6%	4.3%	6.1%	5.9%	5.6%	4.8%	4.2%	3.5%	2.9%
Appliances	3.6%	3.5%	3.5%	3.5%	3.6%	3.7%	3.8%	3.9%	4.0%	4.1%	4.3%	4.4%	4.6%	4.7%	4.8%	4.9%	4.9%	4.8%	4.7%	4.6%	4.5%	4.2%	3.9%	3.7%
**Urban**	**3.0%**	**3.0%**	**3.0%**	**3.0%**	**3.2%**	**4.5%**	**4.8%**	**3.9%**	**3.5%**	**3.7%**	**4.3%**	**4.3%**	**4.1%**	**4.3%**	**4.2%**	**4.2%**	**4.9%**	**5.6%**	**5.5%**	**5.9%**	**5.5%**	**4.4%**	**3.8%**	**3.3%**
Heating	2.5%	2.5%	2.5%	2.5%	2.5%	2.5%	2.5%	2.5%	3.9%	3.9%	3.9%	3.9%	3.9%	6.8%	6.8%	6.8%	6.8%	6.8%	6.8%	6.8%	3.9%	3.9%	2.5%	2.5%
Cooling	2.1%	2.1%	2.1%	2.1%	2.1%	4.8%	6.5%	4.8%	2.5%	3.9%	9.2%	7.4%	4.8%	3.4%	2.5%	3.0%	7.4%	10.1%	7.9%	2.5%	2.5%	2.1%	2.1%	2.1%
Hot water	4.9%	4.9%	4.9%	4.9%	4.9%	4.9%	3.3%	3.3%	3.3%	3.3%	3.3%	3.3%	3.3%	3.3%	3.3%	3.3%	3.3%	4.9%	4.9%	4.9%	4.9%	4.9%	4.9%	4.9%
Refrigeration	4.2%	4.2%	4.2%	4.2%	4.2%	4.2%	4.2%	4.2%	4.2%	4.2%	4.2%	4.2%	4.2%	4.2%	4.2%	4.2%	4.2%	4.2%	4.2%	4.2%	4.2%	4.2%	4.2%	4.2%
Lighting	0.7%	0.7%	0.7%	0.7%	1.7%	9.9%	10.4%	3.4%	1.9%	1.4%	1.4%	1.4%	1.4%	1.4%	1.4%	1.4%	2.6%	4.4%	6.6%	14.9%	15.4%	7.7%	5.7%	2.7%
Cooking	2.7%	2.7%	2.9%	3.1%	3.5%	4.0%	5.7%	5.5%	3.5%	3.7%	3.5%	5.7%	5.3%	3.9%	3.6%	3.6%	4.3%	6.1%	5.9%	5.6%	4.8%	4.2%	3.5%	2.9%
Appliances	3.6%	3.5%	3.5%	3.5%	3.6%	3.7%	3.8%	3.9%	4.0%	4.1%	4.3%	4.4%	4.6%	4.7%	4.8%	4.9%	4.9%	4.8%	4.7%	4.6%	4.5%	4.2%	3.9%	3.7%
**Transport**	**6.9%**	**7.4%**	**7.0%**	**6.2%**	**5.2%**	**4.2%**	**3.4%**	**2.8%**	**2.4%**	**2.0%**	**1.8%**	**1.7%**	**1.7%**	**1.7%**	**1.7%**	**1.9%**	**2.3%**	**2.9%**	**3.6%**	**4.6%**	**5.8%**	**6.9%**	**7.8%**	**8.0%**
Fast	1.7%	5.1%	4.7%	2.8%	1.1%	0.3%	0.7%	2.0%	3.9%	6.0%	7.7%	8.9%	9.1%	8.5%	7.1%	5.3%	3.4%	1.9%	1.3%	1.6%	3.0%	4.7%	5.6%	3.6%
Slow	7.8%	7.8%	7.5%	6.8%	5.9%	4.9%	3.9%	3.0%	2.1%	1.4%	0.8%	0.5%	0.3%	0.5%	0.8%	1.4%	2.1%	3.0%	4.1%	5.1%	6.2%	7.3%	8.1%	8.7%

Supplementary Table 3 The percentage of energy consumption in different hours of the day in the building and transportation sectors.

The percentage of energy use by time period in the building sector refers to the national standard GB 55015-2021 “General specifications for building energy efficiency and renewable energy use”. The percentage of energy use by time period in the transportation sector refers to the “New Energy Vehicle Industry Development Plan (2021-2035)”. The data is processed by spline smoothing.

Comment 3.13:

Line 174-175. You state that "Concurrently, the load profile undergoes a transformation from a relatively flat distribution to one characterized by daytime peaks, thereby aligning more effectively with the PV power generation". Which load profile do you mean? Could you refer to the corresponding figure here?

Response:

Thank you very much for bringing up the confusing points. What we are trying to convey here is that future loads, guided by prices, will be more concentrated when renewables are generating large amounts of electricity, and that the comparison is to the electricity load curve for the year 2020, which was not specifically plotted in the original manuscript. We present the load profiles for key years and scenarios in Supplementary Figure 6.

Changes/additions to the manuscript:

(Manuscript, Line 210-213) Comparing the 2060 and 2020 load profiles (Supplementary Figure 6a), the load profile undergoes a transformation from a relatively flat distribution (reducing thermal power regulation operations) to one characterized by daytime peaks (better matching PV generation).

(Supplementary Information, Supplementary Figure 6) Add Supplementary Fig. 6a.

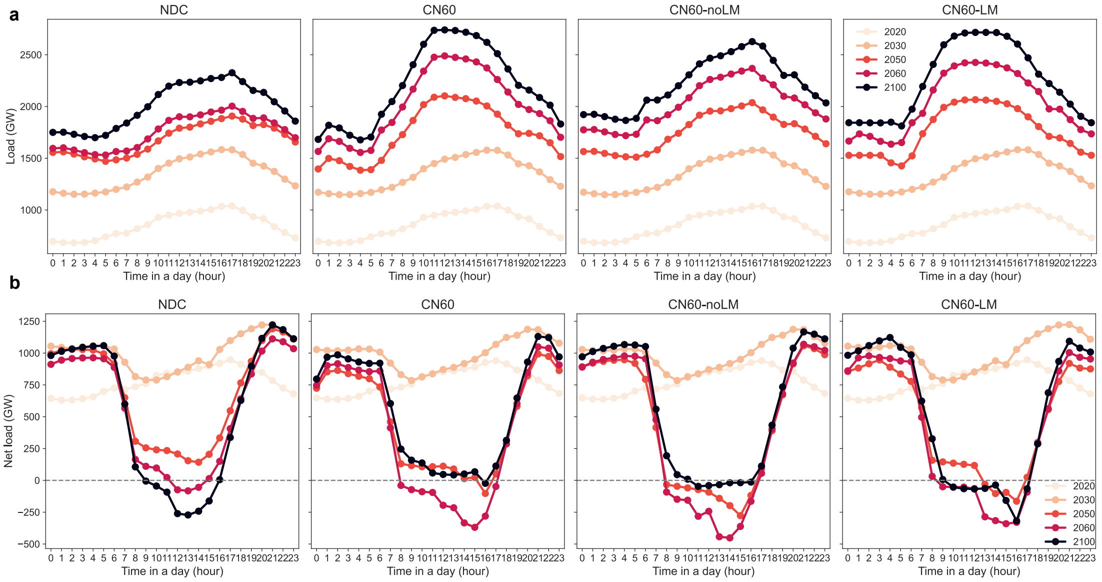

Supplementary Fig. 6 The load and net load variation of the typical summer day in China

a The real electricity load (except storage charging), **b** The net load. The net load is the difference between the real electricity load (except storage charging) and the output of variable renewables (wind and PV power generation).

Comment 3.14:

Line 185-186. You write that "Nowadays, fossil fuels and district heating are the primary sources for satisfying the heating demand, while electricity almost exclusively fulfils cooling needs." Is that district heating also fuelled by fossil fuels (e.g. Gas) in China?

Response:

Thanks for your question. Nowadays, district heating is mainly supplied by fossil fuels, such as coal-fired CHP and gas-fired heat boilers. However, low-carbon heating modes such as industrial waste heat and nuclear energy heat are now being piloted in some places, and this mode of district heating will be free from fossil fuels.

Changes/additions to the manuscript:

(Manuscript, Line 223-224) Nowadays, fossil fuels and district heating (mostly fossil fuels) are the primary sources for satisfying the heating demand,

---

Comment 3.15:

The figures cited in the text are inconsistent with the actual figures. Figure 5b is electricity consumption in the transportation sector on a summer workday, while Figure 5d is missing. Figure 6e is marginal electricity supply costs for different time periods on a summer workday.

Response:

Thank you very much for pointing out the inconsistencies in the figure citations. We have revised the order and combination of figures against the journal's figure formatting requirements, and we have double-checked the full-text figure citations in the revised manuscript.

Changes/additions to the manuscript:

(Manuscript, Line 243) adjustable load and V2G technology play a pivotal role in enhancing system flexibility during periods of severely constrained online capacity by facilitating intraday storage arbitrage (Fig. 6b).

(Manuscript, Line 246) these interactions significantly diminish the reliance on power-based energy storage, such as pumped hydro (Fig. 4d).

(Manuscript, Line 256) This, in turn, can effectively lower the marginal cost of electricity (Fig. 8e)

Comment 3.16:

Line 224-230 (line 459-468). Provide more detail on how demand shifting is achieved within the modelling framework. Price elasticity reducing demand level is not shifting the load. Is demand shifting to that described by Li et al. - https://www.sciencedirect.com/science/article/pii/S0306261918310237. More elaboration is needed.

Response:

Thank you very much for mentioning this. Both our paper and the study you mentioned for the UK are based on the TIMES framework and have a similar modeling formulation of time-shifting of loads. However, an improvement over this is the introduction of the amount of value of the load time shift in our model. Based on a three-hour load time-shift subsidy rate of 1 CNY/kWh for Yunnan Province, the model conservatively assumes that the subsidy per hour shifted (whether advanced or delayed) is about 4 US cents/kWh.

In response to your question about price elasticity, it is not our intent to indicate that price elasticity enables load hourly shifting. Rather, we are applying price elasticity, which has traditionally worked only on total annual demand, to the hourly scale, thereby enabling price-based load reduction (curtailable load), which is an important way in which demand-side response provides system flexibility.

Changes/additions to the manuscript:

(Manuscript, Line 558-565) Based on the TIMES open-source framework[69,70], the China TIMES 2.0 model models load shifting as a special bi-directional energy storage technology that is able to represent the maximum allowed deviations from nominal demand loads, maximum advance or delay for meeting the shifted loads, and the value per hour shifted by setting the storage capacity, the storage time, and the operating cost, respectively. The model conservatively assumes a subsidy of approximately 4 cents/kWh per hour of shifting, whether advanced or delayed, based on a subsidy of RMB 1/kWh for three-hour load shifting in Yunnan Province.

Comment 3.17:

As with other sections, clarity on understanding the additional implications of improved temporality on system costs would be useful.

Response:

Thank you very much for your valuable suggestions. In terms of total system cost per capita, there will be a clear bifurcation between scenarios that consider sub-annual modeling and those that do not after 2040. In 2060, sub-annual modeling will push up system costs by about 4% (CN60 vs CN60-noTS), while not considering demand-side management measures will further push up system costs by nearly 1% (CN60 vs CN60-noLM). Enhanced time resolution places more demand on flexibility resources, and the differences between scenarios are mainly in the areas of electricity, hydrogen and energy storage. In terms of power supply costs, consideration of sub-annual level modeling would increase costs by 3% until 2030, with a modest cost differential between 2030-2050 and then again after 2050 to about 3%. In terms of hydrogen costs, the average increase compared to CN60-noTS amounts to 14% from 2035-2060 and 27% in 2035. This also suggests that sub-annual modeling better identifies hydrogen energy application scenarios and supply patterns. In terms of the cost of energy storage, it is even more significant, and the gap gradually widens as the share of renewable energy increases, with the scenario considering sub-annual modeling rising 24% over the CN60-noTS scenario by 2060. The degree of demand-side management has a significant impact on energy storage investment, with the CN60-LM scenario reducing investment by 10% compared to the CN60 scenario, while the CN60-noLM scenario increases investment by more than 12% compared to the CN60 scenario.

We have added the CN60-noTS scenario in Supplementary Fig. 13 so that we can more intuitively see the impact by considering sub-annual constraints.

Changes/additions to the manuscript:

(Manuscript, Line 270-274) Considering the system operating constraints at the sub-annual level, the total system cost would rise by about 4% compared to not considering it. Enhanced time resolution places more demand on flexibility resources, and the differences between scenarios are mainly in the areas of electricity, hydrogen and energy storage. (Supplementary Fig. 13).

(Manuscript, Line 279-284) By considering the sub-annual operation process, we identified that hydrogen could take advantage of low electricity price periods to reduce production costs and thus expand application. Comparing CN60 with CN60-noTS, the annual expense of hydrogen increases by 14% from 2035 to 2060, and by 27% especially in 2035. It has been demonstrated that the CN60 increases energy storage expenses by 24% over the CN60-noTS by 2060.

(Manuscript, Line 287-290) Demand-side response exerts a substantial influence on energy storage expenditures. A 10% reduction in expenses is observed for the CN60-LM scenario relative to the CN60 scenario, while the CN60-noLM scenario

demonstrates an expense increase of more than 12% compared to the CN60 scenario.

(Supplementary Information, Supplementary Fig.13) add CN60-noTS scenario.

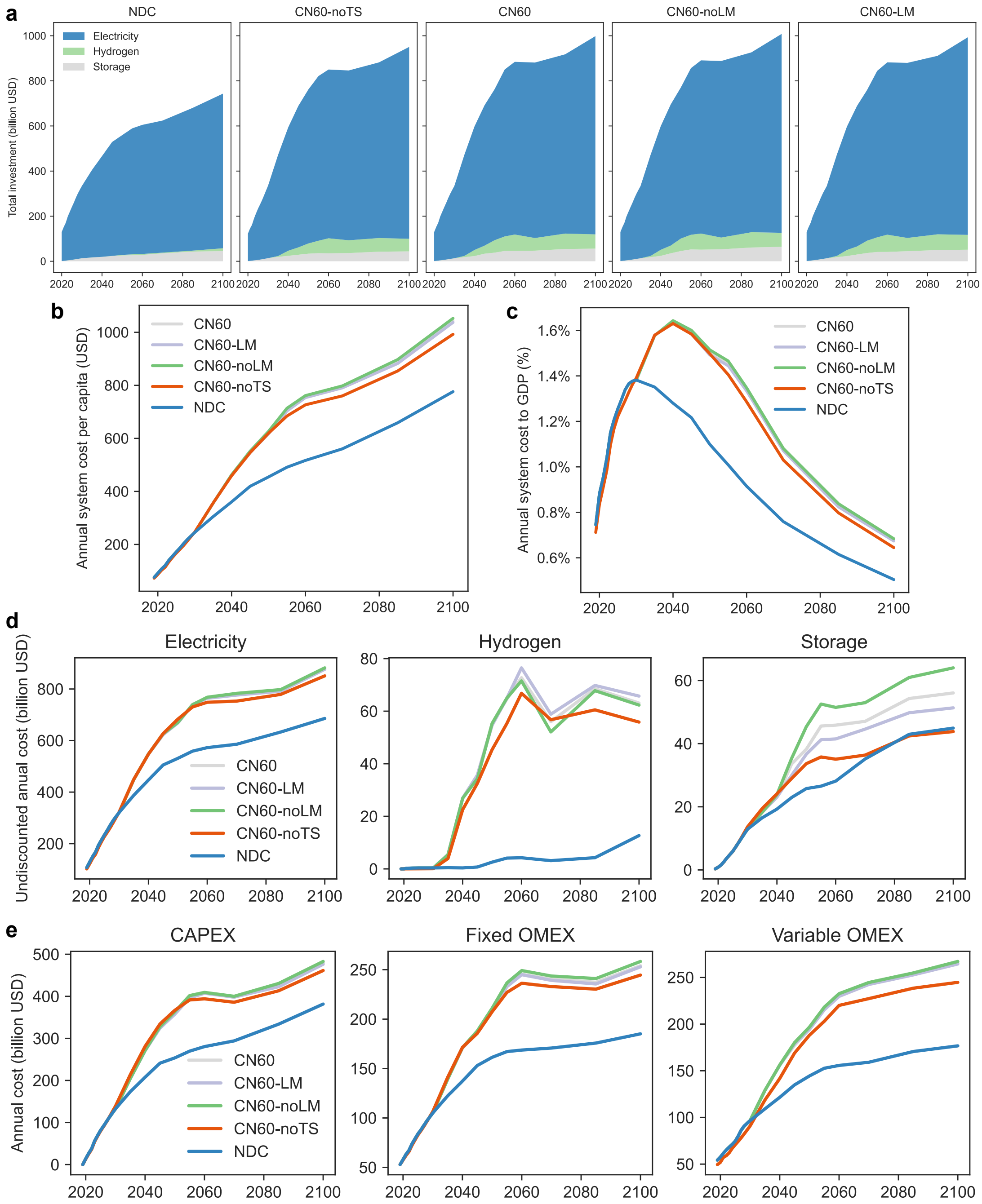

Supplementary Fig. 13 Energy transition cost in electricity, hydrogen and storage for China during 2020-2100. a Annual system investment. **b** Undiscounted annual per capita system cost. **c** Undiscounted annual system costs as a share of the year's GDP. **d** Undiscounted annual system costs by sector. **e** Undiscounted annual system costs by type of cost.

Comment 3.18:

Discussion: Develop this section more towards the implications of the results for decision makers, and for the modelling community.

Response:

Thank you very much for your suggestions. We have implementation suggestions for policymakers and the modeling community in the revised version of the manuscript.

Changes/additions to the manuscript:

(Manuscript, Line 321-323) Based on our findings, we offer the following insights into demand management, power planning, storage deployment, demand-side response, and market mechanisms for flexibility enhancement and energy system transformation

(Manuscript, Line 330-333) Therefore, the construction of charging infrastructure should gradually increase the fast-charging proportion and guide the coordination of charging load with renewable energy output through market regulation, policy incentives and intelligent instruments.

(Manuscript, Line 339-341) Enhancing regulation performance and CCS retrofits of CFPPs could mitigate asset stranding concerns, thereby facilitating a viable transition path for massive CFPPs.

(Manuscript, Line 353-358) We recommend accelerating pumped hydro construction and actively promoting variable speed pumped hydro to effectively manage large-scale, rapid power fluctuations. We propose to strengthen the integration of new-type energy storage with the development planning of distribution grids, renewables, EVs, and to put efforts into the grid-forming energy storage, to further enhance the ability of energy storage to support the operation of power grids.

(Manuscript, Line 380-386) Specifically, we advocate for the following measures: widening the peak-valley price difference, expanding the floating range of tariffs and the upper and lower price limits, enriching the trading varieties of auxiliary services with technical characteristics of dispatchable resources such as ramping and system inertia, and, with reference to CFPP and pumped hydro, establishing a capacity tariff mechanism for new-type energy storage to break up the unfair competition of flexible resources.

(Manuscript, Line 393-400) The challenges of ensuring power system security during the net-zero transition are evidenced by recent global trends, including more frequent power interruptions, load control, and electricity price volatility. A pressing need has emerged within the broader IAMs community and among energy system modelers to provide sub-annual perspectives that consider the novel changes precipitated by the

substantial integration of renewable energy and the emergence of new electric load access. To enhance the feasibility of long-term mitigation pathways, it is imperative to devise cost-effective system flexibility regulation mechanisms.

Comment 3.19:

Line 273-274. ‘Most economical’ compared to what other options?

Response:

Thank you very much for your question. The other options here we would like to stand in for non-demand-side traditional means such as peaking power, energy storage, reserve capacity and so on. We have tried to minimize this more absolute formulation in the revised version.

Changes/additions to the manuscript:

(Manuscript, Line 324-326) Effective demand management is a more economical means of balancing energy supply and demand than making investments and dispatching power plants and storage.

Comment 3.20:

Line 279-289. While interesting, are these results pertinent to the main argument of the paper?

Response:

Thank you very much for your thought-provoking questions. Here we may not have passed on background knowledge of China's coal power development, thus creating a leap of logic. In 2021 the National Development and Reform Commission (NDRC) and the National Energy Administration (NEA) proposed to carry out energy-saving and carbon-reducing retrofits, heat supply retrofits, and flexibility retrofits (the Three Retrofits) for coal power units, in 2024 the NDRC and the NEA proposed to carry out the three major decarbonization retrofits such as biomass blending retrofits, ammonia blending retrofits, and carbon capture retrofits for coal power units. In March 2025, the (NDRC and the NEA released the Implementation Plan for Coal Power Upgrading Special Action (2025-2027), which emphasized the need to enhance the flexibility of coal power operation capabilities such as deep peaking, rate of change of loads, starting and stopping peaking, and high efficiency of wide loads. Therefore, we believe that our discussion of retrofitting existing CFPPs and new-built CFPPs is of great

interest to policymakers and must be considered to address the transformation of China's coal power. The topic of this paper is about the need for a integrated assessment of net-zero paths at the sub-annual level, and the flexibility retrofit of coal power happens to be an important source of power system flexibility.

Comment 3.21:

Scenario setting: The scenarios are distinct but need more clarification of common assumptions and differentiated assumptions.

Response:

Thank you very much for your suggestion, we have significantly improved the description of common assumptions and differences between scenarios.

Changes/additions to the manuscript:

(Manuscript, Line 574-578) In addition, we propose a scenario CN60-noTS without sub-annual constraints, which removes the seasonal, weekly, and daily variability carve-outs of renewable energy outputs and loads (assumed to be averaged distributions) and relaxes the power unit flexibility constraints.

(Manuscript, Line 579-581) For all net-zero scenarios (CN60-noTS, CN60, CN60-noLM and CN60-LM), we set the cumulative carbon emissions for 2019-2100 under RCP2.6 assumption based on the carbon budget allocation principle (230 $GtCO_2$).

(Manuscript, Line 588-592) For NDC and all net-zero scenarios, we set capacity limits for coal, nuclear, wind, and PV power plants based on policy and resource potential. For all end-use sectors, we assumed no further increase in the share of fossil fuel. In addition, for all scenarios, we require total carbon emissions to decline monotonically after peaking (no rebound).

(Manuscript, Line 593-607) The difference in assumptions between scenarios centers on the difference in the degree of demand-side contribution to system flexibility. CN60-noLM represents a scenario where no demand response measures are implemented, and the energy user consumes energy in the same way as they do presently. This represents the case where energy prices do not affect demand-side behavior at all. CN60 is an intermediate scenario that envisions the electricity demand for transportation and hydrogen production adapting to variations in online electricity generation capacity. In this scenario, energy prices have an impact on demand that can be easily regulated, such as hydrogen production and EV charging, and users regulate the time distribution of energy use in response to price signals. Meanwhile,

CN60-LM is a supply-demand interaction scenario that introduces load time shifting across a wide range of industries alongside V2G technology and home energy storage feed-in. In this scenario, most of the energy service demands are capable of demand-side response with price incentives, and in particular, EV batteries, and home energy storage can be dispatched to balance system supply and demand.

Comment 3.22:

Line 470-423. In your NDC scenario, does it include over 65% reduction in carbon intensity and other renewable related target?

Response:

Thank you very much for your question. Our NDC scenarios include China's carbon intensity reduction target (>65% reduction) and renewable energy development target (>1200GW solar and wind capacity) for 2030 in the updated NDC.

Changes/additions to the manuscript:

(Manuscript, Line 569-571) Carbon intensity reduction of more than 65% from 2005 and wind & solar capacity exceeding 1200GW targets are bounded in the NDC scenario.

Comment 3.23:

Line 476-477. The statement "We set the cumulative carbon emissions for 2019-2100 under RCP2.6 assumption based on the carbon budget allocation principle (230 GtCO2)", needs reference or clarification of the allocation principle, and why is it applied for all your scenarios?

Response:

Thank you very much for your question. As you mentioned, there is a large uncertainty in allocating carbon budgets to countries from RCP2.6 or the two-degree target, and each country has its own preferred allocation principles. However, for the sake of multi-model comparability, we have adopted the proposal suggested by the ENGAGE project. The global carbon budget allocation is calculated according to the "Equal Cumulative Per Capita (ECPC)" methodology, with a historical allocation starting point in 1990, and a global pathway that selects the median pathway of the IPCC Sixth Assessment Report Category C4 (limit warming in 2 degrees with >50% probability)

Changes/additions to the manuscript:

(Manuscript, Line 581-585) The global carbon budget allocation is calculated according to the "Equal Cumulative Per Capita (ECPC)" methodology, with a historical allocation starting point in 1990, and a global pathway that selects the median pathway of the IPCC Sixth Assessment Report Category C4 (limit warming in 2 degrees with >50% probability).

Comment 3.24:

SI Figure 2, why does coal use rebound in 2060 in urban areas within the hot summer–cold winter zone under your NDC scenario? However, in the same scenario and climate zone, coal use continues to phase down after 2030.

Response:

Thank you very much for your question. The regions that have seen a rebound in coal consumption are all in the south (HSCW, HSWW, M). Buildings in the South do not currently have mandatory space heating requirements, so space heating penetration in 2025 is low, with penetration rates of 29%, 26%, and 9% in the three regions above, while urban space heating penetration in the North is close to 100%. As the quality of life improves, we expect the heating penetration rate in the South to increase significantly, and by 2030 and 2060, the heating penetration rate in the above three regions will reach 34%, 31%, 12% and 62%, 61%, 30%, respectively, and thus drive-up energy consumption. Additionally, the dominant technologies available for space heating in the South include coal boilers, natural gas boilers, electric boilers, air conditioners, and heat pumps. Since we set the share of fossil fuels not to rise, from a cost-optimization perspective, it is relatively inexpensive to choose coal in the NDC scenario with no carbon constraints in 2060, so coal boilers rebound in the face of declining natural gas (which relies heavily on imports and higher fuel costs).

Comment 3.25:

SI Figure 6 lacks legend

Response:

Thank you very much for pointing out this omission, we have added the legend.

Changes/additions to the manuscript:

(Supplementary Information, Supplementary Figure 6) Add legend.

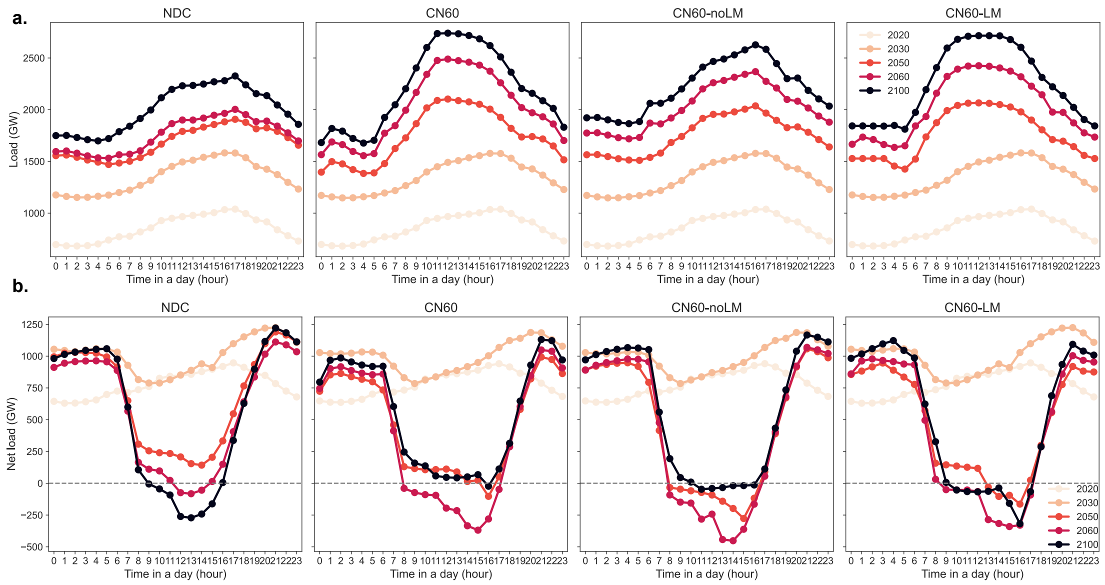

Supplementary Fig. 6 The load and net load variation of the typical summer day in China

a The real electricity load (except storage charging), **b** The net load. The net load is the difference between the real electricity load (except storage charging) and the output of variable renewables (wind and PV power generation).

Comment 3.26:

SI Figure 15 needs more elaboration around data source

Response:

Thank you very much for your question. We have included data on the percentage of energy use in the building and transportation sectors that have large differences in temporal distribution in the new Supplementary Table 3.

The percentage of energy use by time period in the building sector refers to the national standard GB 55015-2021 “General specifications for building energy efficiency and renewable energy use”. The percentage of energy use by time period in the transportation sector refers to the “New Energy Vehicle Industry Development Plan (2021-2035)”. The agriculture sector is assumed to have flat electricity use curve, and the total load residual term excluding building, transportation, and agriculture is considered as the electricity consumption curve for the industrial sector.

Changes/additions to the manuscript:

Supplementary Information, Supplementary 14 caption) The load profile of the building sector refers to the national standard GB 55015-2021 “General specifications for

building energy efficiency and renewable energy use”. The load profile of the transportation sector refers to the “New Energy Vehicle Industry Development Plan (2021-2035)”. The agriculture sector is assumed to have flat electricity use curve, and the total load residual term excluding building, transportation, and agriculture is considered as the electricity consumption curve for the industrial sector.

Reviewer 4

I co-reviewed this manuscript with one of the reviewers who provided the listed reports. This is part of the Nature Communications initiative to facilitate training in peer review and to provide appropriate recognition for Early Career Researchers who co-review manuscripts.

Response:

Thanks very much for your invitation of Early Career Researchers to give us helpful comments.

Response to Referees

NCOMMS-25-08417-A

August 25, 2025

Reviewer 1

This version has been properly revised.

Response:

Thank you very much for reviewing this paper and for your valuable comments.

Reviewer 2

Comment 2.1:

Sensitivity analysis should be conducted considering different V2G participation rates in the assumptions of demand-side response?

Response:

Thank you very much for your suggestion. Based on the CN60-LM scenario, we ran two additional scenarios and made differentiated adjustments to the participation level of V2G. Specifically, we changed the proportion of the maximum activity level of V2G in each timeslice to the electricity consumption of the transportation sector in different scenarios. The model is then optimized according to the principle of cost optimization.

Scenario	2030	2050	2110
CN60-LM	20%	30%	50%
CN60-LM-lowV2G	20%	20%	20%
CN60-LM-highV2G	40%	60%	70%

The results show that an increase in the proportion of V2G applications can promote the consumption of VRE while reducing the operation of energy storage systems. V2G is first applied to support peak loads and ramping, and when the application rate is higher, it further substitutes for energy storage.

We have included the sensitivity analysis and additional figures in the supplementary information, with references in the main text.

Changes/additions to the manuscript:

(Manuscript, Line 224-226) The result of sensitivity analysis of different V2G application rates can be found in Supplementary Notes 3 and Supplementary Figures 18-19.

(Supplementary Note 3 and Supplementary Figure 18-19)

Supplementary Note 3 Sensitivity scenario analysis for V2G application rates

Demand response, such as V2G, can alleviate peak loads and reduce energy storage requirements. Based on the CN60-LM scenario, we ran two additional sensitivity scenarios and made differentiated adjustments to the participation level of V2G. Specifically, we changed the proportion of the maximum activity level of V2G in each timeslice to the electricity consumption of the transportation sector in different scenarios. For the CN60-LM scenario, in 2030, 2050, and 2110, the maximum activity level for charging or discharging in each timeslice are 20%, 30%, and 50% of

the original transportation electricity demand for that timeslice, respectively. For the LowV2G scenario, these limits are 20%, 20%, and 20%, respectively. For the HighV2G scenario, these limits are 40%, 60%, and 70%, respectively.

From the power supply on a typical day, a higher V2G application rate (HighV2G scenario) can promote the consumption of wind power and solar power at noon (approximately 50GW), while reducing the charging and discharging of energy storage. V2G facilities primarily charge during midday and discharge in the evening. Conversely, the LowV2G scenario significantly weakens the role of V2G, resulting in a noticeable flexibility shortage around 9 PM, necessitating increased output from various power plants such as nuclear, coal-fired, and biomass to provide flexibility.

Focusing on V2G operations, it can be observed that the primary charging period for V2G vehicles is between 8 and 10 am, while the discharge period is between 8 and 9 pm. By increasing the utilization rate of V2G applications, charging during the 9 am to 2 pm period can be significantly enhanced, while discharge during the 6 pm to 11 pm period also sees an overall improvement. Through sensitivity analysis, it can be summarized that the most economical role of V2G is to provide power support during peak load and ramping periods. Under conditions of higher V2G application rates, it can partially replace the functions of energy storage facilities.

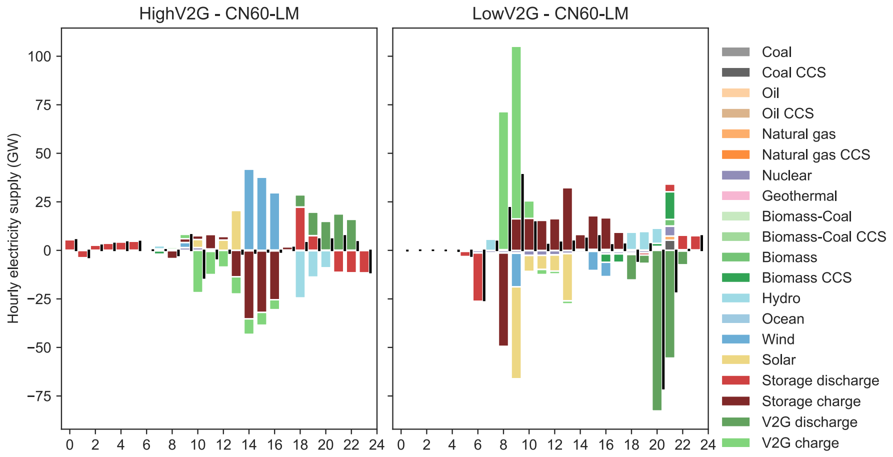

Supplementary Fig. 18 Differences in electricity supply by technology for the typical day in 2060 between the V2G application rate sensitivity scenario and the core scenario

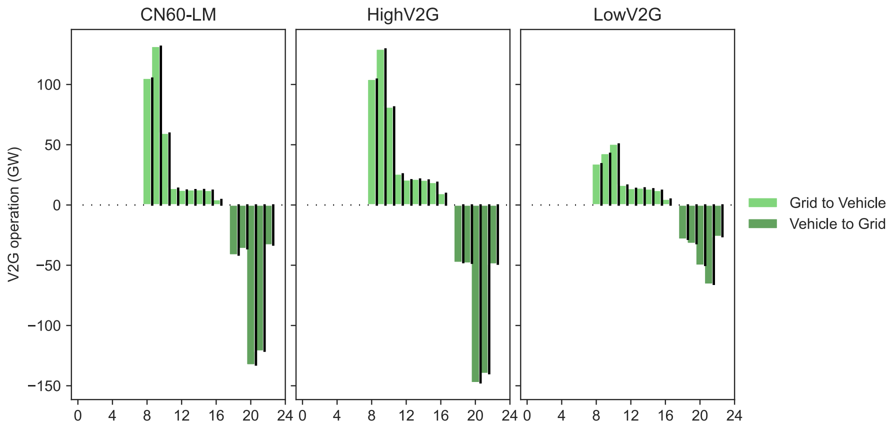

Supplementary Fig. 19 V2G operations in 2060 under different scenarios

---

Comment 2.2:

The sensitivity analyses should also consider different assumed costs for the flexibility retrofits of coal-fired power plants?

Response:

Thank you very much for your suggestion. Your comment is very important. The cost and pace of retrofitting will indeed have a certain impact on the development and operation of thermal power and nuclear power. Based on the CN60 scenario (retrofitting cost of 500 CNY/kW), we further ran two additional scenarios: CN60-lowCost and CN60-highCost, corresponding to retrofitting costs of 200 CNY/kW and 1000 CNY/kW, respectively.

The results indicate that flexibility retrofits for existing coal-fired power plants are both necessary and cost-effective in the near term. Both high-cost and low-cost retrofit scenarios show high retrofit rates. However, in the high retrofit cost scenario, by 2060, daytime renewable energy integration will face greater challenges, with energy storage and gas-fired generation taking on more flexible regulation tasks. In the low retrofit cost scenario, nuclear power demonstrates greater flexibility, enabling it to increase load during the evening hours when flexibility is scarce and reduce load during midday hours.

We have included the sensitivity analysis and additional figures in the supplementary information, with references in the main text.

Changes/additions to the manuscript:

(Manuscript, Line 150-153) CFPP flexibility retrofits can significantly improve the dispatch performance of existing assets, partially alleviating the lack of flexibility

(Supplementary Notes 2 and Supplementary Figure 16-17).

(Supplementary Note 2 and Supplementary Figure 16-17)

Supplementary Note 2 Sensitivity scenario analysis for flexibility retrofits

Flexibility retrofits can help fully utilize existing large-scale thermal power assets to provide flexibility to the energy system. The cost and pace of retrofitting will indeed have a certain impact on the development of thermal power and nuclear power. In the model, retrofit technologies are provided for coal-fired, nuclear, and biomass power generation units (both with and without CCS). Based on the CN60 scenario (retrofitting cost of 500 CNY/kW), we further ran two additional scenarios: CN60-lowCost and CN60-highCost, corresponding to retrofitting costs of 200 and 1000 CNY/kW, respectively.

Sensibility scenario simulations indicate that, given the relatively high investment costs associated with energy storage and other flexibility technologies, flexibility retrofits remain cost-effective for most power plants. In the CN60-highCost scenario, solar power generation is projected to decline moderately by 2060, while wind and nuclear power generation are expected to increase correspondingly. Over the 2020–2060 period, total electricity generation from all power sources will experience minor fluctuations. In the CN60-lowCost scenario, the impact on total electricity generation is minimal, with coal-fired power generation with CCS showing a moderate increase between 2045 and 2055.

From the hourly operation perspective, it can be seen that under the CN60-highCost scenario, daytime solar power generation will decrease, while wind power generation will increase slightly throughout the day. In the evening, gas-fired power plants and energy storage will play a greater role in providing flexibility. Under the CN60-lowCost scenario, some nuclear power plants will participate in flexibility renovations and engage in peak shaving, while wind power and solar power generation will not be significant. At the same time, activities related to flexible hydropower and energy storage have also decreased relatively.

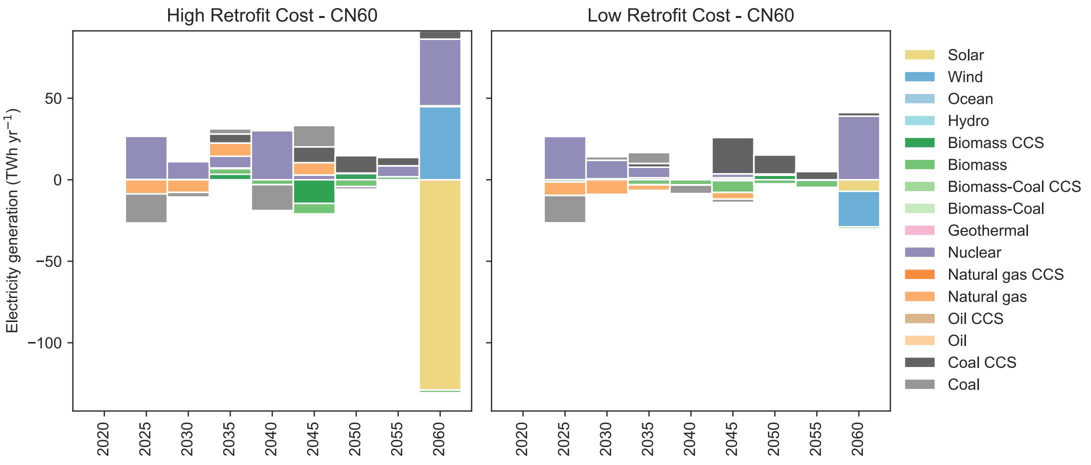

Supplementary Fig. 16 Differences in electricity capacity by technology between the flexibility retrofit sensitivity scenario and the core scenario

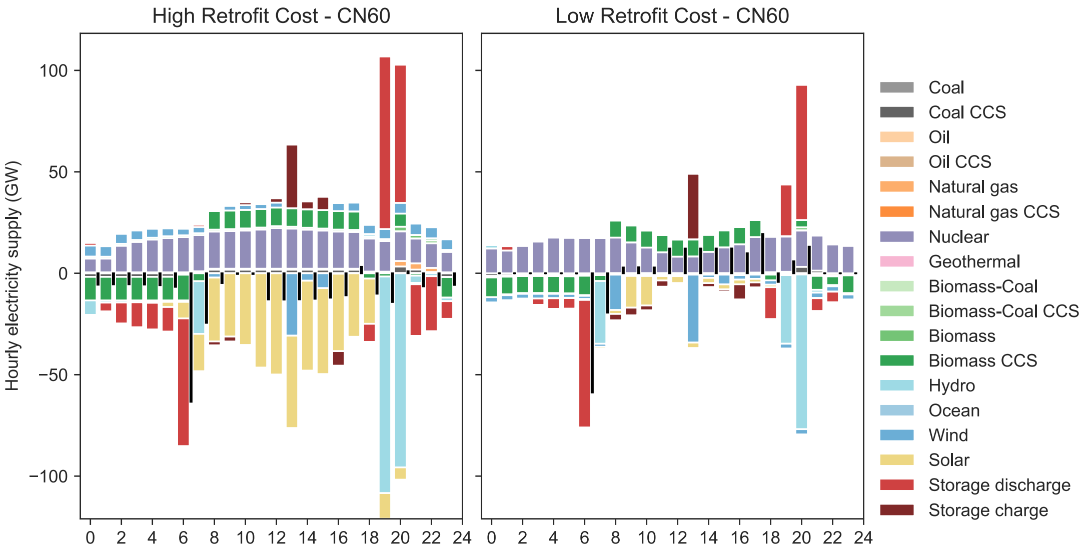

Supplementary Fig. 17 Differences in electricity supply by technology for the typical day in 2060 between the flexibility retrofit sensitivity scenario and the core scenario

Reviewer 3

General Comment:

Thank you for the authors' detailed and thoughtful responses to the first-round comments. I appreciate the substantial effort made to address the issues raised, including the additional analyses, clarifications, and the inclusion of new tables and data where requested. These revisions have strengthened the manuscript considerably and improved its transparency.

Response:

Thank you very much for your review. Your comments have helped us further refine the novelty and key points of our paper. We hope that our revisions to the manuscript in this round will adequately address your concerns. Due to changes in the novelty description and narrative style of the paper, additional revisions not mentioned in the following comments are highlighted in color in the manuscript.

Comment 3.1:

In response to comments 1.1 and 3.8 regarding the paper's novelty, the three core comparisons presented are clear. However, I remain unconvinced, as your sub-annual modelling approach and key quantitative findings primarily demonstrate that traditional annual-based IAM scenario analyses tend to idealize the pace of thermal power phase-out and overestimate the scale of renewable deployment—an issue that is already widely acknowledged. That said, the strength of your model in exploring specific pathway combinations can indeed support China in identifying more feasible power system transition pathways. I would therefore recommend positioning the paper's novelty around the feasibility of these transition pathways with flexibility management included, rather than framing sub-annual modelling itself as the core innovation. If so, I also suggest refining your key quantitative conclusions in the abstract accordingly.

Response:

We appreciate the suggestion to position our contribution around feasibility with flexibility management. We have revised the Title, Abstract, Introduction, and Discussion to emphasize that our core contribution is to quantify feasibility and least-regret portfolios for China's energy transition when flexibility options are co-optimized in a sub-annual framework.

Now, sub-annual modeling is presented as an enabling methodological enhancement, not the novelty itself. We now focus key quantitative conclusions on feasibility and flexibility rather than on differences vs an annual model and highlight

the unique integration of demand-side flexibility and V2G in an economy-wide framework.

Changes/additions to the manuscript:

(Manuscript, Title) Exploring the feasible net-zero transition pathway in China considering energy system flexibility

(Manuscript, Abstract)

Net-zero energy transition is projected to accelerate the replacement of fossil fuels with renewables, leaving system flexibility resources increasingly scarce. Here, we present a sub-annual energy-environment-economy model with endogenous hourly energy demand profiles and power balance dynamics including power dispatch, storage operations and demand-side response that co-optimizes supply- and demand-side flexibility, to map feasible transition pathways for China. The results show that compared with coarser timeslice representative of common modelling practice, sub-annual representation tightening flexibility needs with a high variable renewable energy (VRE) and high electrification energy system. Results reveal steeper load ramps and frequent evening price strikes, identify increased activities in thermal power, nuclear power, hydrogen production, and energy storage. Accounting for temporal variability in supply and demand, the cost-optimal solution exhibits marginal abatement costs that are over 9% higher, but incorporating demand-side flexibility measures can mitigate cost growth and delineates least-regret portfolios for reliable, affordable decarbonization. Incentives for demand-side response such as load time-shifting and vehicle-to-grid (V2G) can reduce investment in pumped hydro by 23% and yield more than a threefold cost-benefit ratio. The study highlights enhanced modelling of temporal dynamics within future energy model development and incentive-compatible market mechanism design for dispatchable resource development.

(Manuscript, Line 43-46) Multi-sector energy system model and integrated Assessment Model (IAM) are designed to provide a quantitative description of coupled human and natural system processes and their interactions, having been widely applied in long-term planning studies in energy and wider domains.

(Manuscript, Line 65-72) In this study, we enhanced the modeling of temporal dynamics in an energy-environment-economy model, China TIMES 2.0, using 56 representative timeslices nested at the annual-seasonal-weekly-daily-hourly to assess energy transition under operational constraints and flexibility enhancement measures. This approach establishes an integrated framework that aligns long-term strategic planning with the complexities of short-term operational dynamics. Based

on industry-level energy consumption pattern modeling, the amplitude and shape of the power load curve are optimized endogenously.

(Manuscript, Line 269-270) With the annual-seasonal-weekly-daily-hourly nesting modeling framework, we observe the dynamics of the energy transition with operational constraints.

(Manuscript, Line 280-285) The booming EV charging and hydrogen production via electrolysis are poised to become valuable resources for managing flexible loads in the coming years. Given that daytime fast charging is a cheap, clean, and flexibility-enhancing option, charging infrastructure construction should gradually increase the fast-charging proportion and guide the coordination of charging load with VRE output through market regulation, policy incentives and intelligent instruments.

(Manuscript, Line 286-287) For safety scheduling and adequate supply considerations, China has accelerated the approval process for new CFPPs in the past 2 years[38].

(Manuscript, Line 291-294) Therefore, enhancing regulation performance of CFPPs and promoting low-carbon retrofits are essential options for mitigating the risk of stranded assets. In a high-VRE power system, hydro, abated CFPPs and BECCS need to reposition and transform into primary flexibility providers.

(Manuscript, Line 295) Diversified energy storage portfolio addresses various flexibility needs.

(Manuscript, Line 312-314) Conventional objectives in power dispatch are progressively proving ineffective in aligning with the optimal configuration requirements for system flexibility in high-VRE power systems.

(Manuscript, Line 316-317) Current time-of-use tariffs encourage narrowing the peak-to-valley load gap, thereby reducing the need for thermal power regulation for economic efficiency.

(Manuscript, Line 320-327) More importantly, energy consumers must be able to discern and react to price fluctuations, thereby empowering stakeholders to engage in incentive-compliant actions that foster system flexibility. Specifically, we propose the implementation of the following measures: a) The expansion of the peak-valley price discrepancy, b) The augmentation of the floating range of tariffs. c) The diversification of trading varieties in auxiliary service markets, including ramping and system inertia. d) The establishment of a capacity tariff mechanism for new-type energy storage, rectifying inequitable competition among flexible resources.

(Manuscript, Line 333-336) A pressing need has emerged within the broader IAMs community and among energy system modelers to enhanced modelling of sub-annual dynamics and flexibility management measures.

Comment 3.2:

Part of the problem of the current argument of novelty relates to the starting position of the paper, and the scenario design. Under comment 3.4, the statement on the lack of temporal resolution in models needs to be evidenced. You use one reference (8) in lines 48-49, but this paper is not relevant and fails to back this assertion up. Most national TIMES models for example will almost certainly be using a sub-annual timeslice resolution – so this starting position for the paper is not accurate (unless you have references to suggest otherwise). A stronger position would be that while there are some attempts at sub-annual representation in similar models, this paper is arguing for improving this, based on enhanced modelling of temporal dynamics.

Response:

Thank you very much for your suggestion. We acknowledge that our previous statement was too absolute. We have replaced it with a more objective position. Specifically, some national TIMES models represent sub-annual dynamics through coarse timeslices (e.g., seasonal × day/night or day types), while several global IAMs default to annual energy balances with optional timeslices. However, coarse timeslice often does not capture diurnal ramps, electricity scarcity, or the operational synergies from demand-side flexibility and V2G. We now cite model documentation and peer-reviewed studies of representative models, and highlight the research gap of finer temporal dynamics.

Changes/additions to the manuscript:

(Manuscript, Line 46-51) Some national energy system models, such as TIMES-family, employ coarse sub-annual timeslices (timeslices by season and daily period)[8], and several global IAMs operate at annual with limited operational detail, although optional timeslices exist[9,10.] Coarse timeslice modeling, however, often cannot fully capture diurnal ramps, peak load hours, or the operational synergies from demand-side flexibility.

(References) Add references

8 Aryanpur, V., Balyk, O., Daly, H., Ó Gallachóir, B. & Glynn, J. Decarbonisation of passenger light-duty vehicles using spatially resolved TIMES-Ireland Model. *Applied Energy* **316**, 119078 (2022). https://doi.org/10.1016/j.apenergy.2022.119078

9 Huppmann, D. *et al.* The MESSAGE Integrated Assessment Model and t

he ix modeling platform (ixmp): An open framework for integrated and cross-cutting analysis of energy, climate, the environment, and sustainable development. *Environmental Modelling & Software* **112**, 143–156 (2019). https://doi.org/10.1016/j.envsoft.2018.11.012
10 Joint Global Change Research Institute. GCAM Documentation (gcam-v8.2). *Zenodo* (2025). https://doi.org/10.5281/zenodo.15581183

Comment 3.3:

This then calls into question the scenario design, where you have used a ‘noTS’ scenario. The problem of comparison against this scenario is undermined if indeed most national TIMES models do have some level of TS resolution. Comparisons of scenarios with and without TS, and the emerging results, are therefore not surprising – given that you are using quite an extreme comparator i.e. the noTS case.

Response:

We're grateful for your comments. We agree that CN60-noTS is a relatively extreme comparator to the TIMES model with TS characterization.

We conducted further reviews on sub-annual modeling both domestically and internationally. Power models such as SWITCH-China, RESPO, CISPO, and GTEP have relatively fine time granularity (often hourly, or even full-year 8760 hours), but they provide limited insights into future end-use sector transitions. Multi-sector models can generally be categorized into energy economic models, which focus on economic analysis, and energy technology models, which emphasize technological dynamics. Teams in countries such as Switzerland, the UK, the US, and Ireland have conducted sub-annual national TIMES. Most Chinese modeling teams constructed energy economic models, such as CE3METL, CHEER, C-GEM, C-REM, IMED|CGE, and C^{3}IAM|CEEPA, which typically study the dynamics of net-zero transition from a top-down perspective and find it difficult to characterize multiple timeslices. There are relatively few active energy technology models in China. Representative models include China-MAPLE, C^{3}IAM|NET, IPAC-Technology and IMED|TEC. These models typically contain rich technical details and describe technological evolution from the bottom-up perspective, but none of them focus on sub-annual modeling. For the localized version of the international IAM model, MESSAGEix-China is currently developing a sub-annual version (4 seasons × 2 weeks × 6 daily timeslices). GCAM-China 7.0 has been open-sourced, calculating credible capacity by setting contribution coefficients for system flexibility. Our team’s model China TIMES 1.0 considers diurnal variations, and China TIMES-30PE considers 4 seasons × 5 daily timeslices. Hence, in order to enhance the comparison between different time

granularities, we added the CN60-DN scenario, which considers two timeslices for day and night, and the 32-timeslice scenario CN60-noHour, which considers 4 seasons × 2 weeks × 4 daily timeslices. For modifications to the scenario setting description, please refer to the reply to the next comment (Comment 3.4). We modified Figure 2 by adding the CN60-noHour and CN60-DN scenarios. The results of the section "Assessing the energy transition at the sub-annual level" have also been included in the comparison of the two scenarios mentioned above.

Changes/additions to the manuscript:

(Manuscript, Line 105-110) The finer temporal resolution reveals operational constraints that are not visible at coarse resolution which tighten feasible pathways for high VRE shares and fast electrification unless flexibility is enhanced accordingly. In addition, the distinct trajectories of thermal power, green hydrogen, and energy storage in China TIMES 2.0, which differ from other IAMs, clearly demonstrate the unique impact of system flexibility on long-term planning.

(Manuscript, Line 113-117) When temporal variability is explicitly represented, the cost-optimal solution exhibits marginal abatement costs that are over 9% higher and total final energy about 3% lower by 2060 relative to a coarse-timeslice baseline. These differences arise because the model internalizes flexibility requirements and operational constraints that are muted at the annual scenario.

(Manuscript, Line 121-126) Thermal power capacity in 2050 could increase by 16-21% and 30-34% in the coarser and hourly modeling scenario, respectively. From the hourly perspective, we identify thermal power achieves a shift from electricity provider to flexibility provider, mitigating the possible shocks of rapid decommissioning. On the other hand, renewables proportion among energy mix would decrease by 2.2% for CN60-nohour and 3.0% for CN60.

(Manuscript, Line 128-139) The coarse time granularity masks the impact of different flexibility measures. Under the CN60-LM scenario, which considers active demand-side flexibility, photovoltaics (PV) generation would decrease by 2% compared to the annual-scale model, while in the CN60-noLM scenario, which does not include demand-side flexibility measures, it would decrease by 12%. For scenarios considering 2-timeslice (CN60-DN) and 32-timeslice (CN60-noHour) scenarios, power generation would decrease by 3% and 4%, respectively. Demand for dispatchable resources would rise sharply with considerable differences between scenarios, with pumped hydro growing by 43-135% under hourly scenarios, and CN60-noHour and CN60-DN would grow by 90% and 46% respectively. Grid-connected hydrogen production would increase by more than 45% when seasonal

factors are taken into account, but only by 27% when only diurnal variations are considered.

(Figure 2) add CN60-noHour and CN60-DN scenarios.

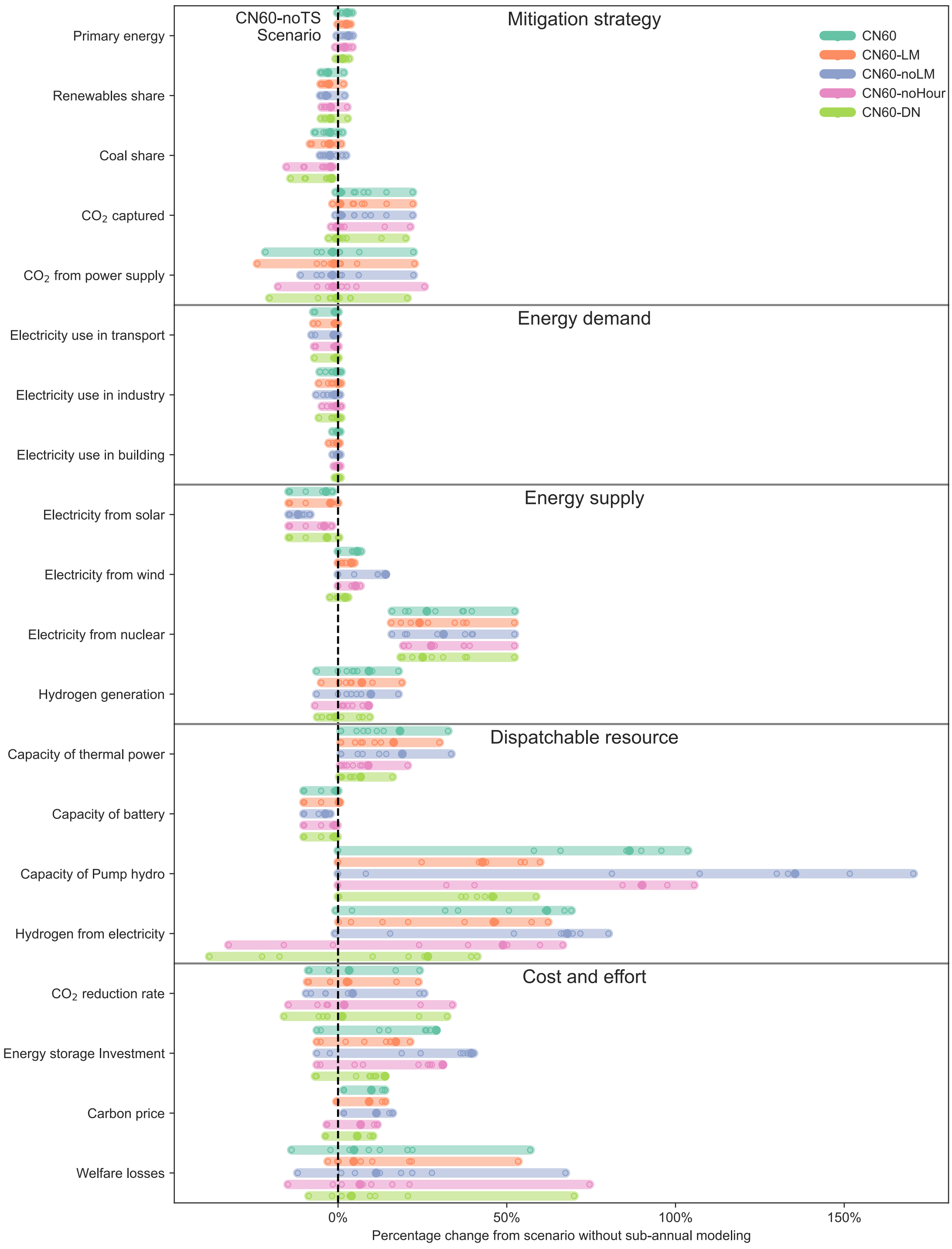

Comment 3.4:

Under 3.6, you state that for noTS ‘Such a scenario is equivalent to the scenario of a

traditional annual-level IAM model without embedded sub-annual level inscriptions.' Is this really the case? Again, please evidence this based on equivalent national models.

Response:

Thank you for pointing this out. We have softened and clarified our wording. Our CN60-noTS setup captures annual energy balances without sub-annual operating constraints; it is not universally "equivalent" to all annual-level IAMs, some of which may include stylized capacity adequacy or flexibility constraints. We restructured the model framework and adjusted different time granularities to represent 1) the status of most multi-sector energy models in China, 2) the time resolution of China TIMES V1.0, and 3) the time granularity commonly used by leading national TIMES for comparison. At the same time, we have also made modifications to the scenario setting section.

Changes/additions to the manuscript:

(Manuscript, Line 89-90) In this study, we propose four hourly scenarios with different flexibility management measures and climate ambition:

(Manuscript, Line 95-100) For comparison, we also present three scenarios considering coarser temporal resolutions: CN60-noTS, CN60-DN, and CN60-noHour, corresponding to no sub-annual modeling (most multi-sector energy system models in China), only considering day and night timeslices (China TIMES 1.0 model), and considering seasonal-weekly-daily modeling but without hourly modeling (common practice in national TIMES models).

(Manuscript, Line 513-516) CN60-DN represents the fluctuations in renewable energy and load considering only daytime and nighttime, which is the practice of the China TIMES 1.0 model[46]. CN60-noHour scenario depicts four seasons, weekdays and weekends, and four time periods in a day, for a total of 32 timeslices, similar to the leading national TIMES model.

Comment 3.5:

The novelty of the paper is likely to be in the innovative way you have introduced load shifting and V2G integration. However, the approach to identifying any insights based on a comparison with noTS is problematic, and doesn' t allow you to highlight improvements versus standard modelling approaches.

Response:

Thanks for your comments. In the text, except for the first subsection of the results, where we use several course timeslice scenarios for comparison, the remaining subsections focus on comparisons between the three scenarios CN60, CN60-noLM, and CN60-LM, which are characterized at the hourly level. We added the explicit description of the comparator to the results section in the revised text.

Changes/additions to the manuscript:

(Manuscript, Line 113-116) When temporal variability is explicitly represented, the cost-optimal solution exhibits marginal abatement costs that are over 9% higher and total final energy about 3% lower by 2060 relative to a coarse-timeslice baseline.

(Manuscript, Line 121-126) Thermal power capacity in 2050 could increase by 16-21% and 30-34% in the coarser and hourly modeling scenario, respectively. From the hourly perspective, we identify thermal power achieves a shift from electricity provider to flexibility provider, mitigating the possible shocks of rapid decommissioning. On the other hand, renewables proportion among energy mix would decrease by 2.2% for CN60-nohour and 3.0% for CN60.

(Manuscript, Line 129-139) Under the CN60-LM scenario, which considers active demand-side flexibility, photovoltaics (PV) generation would decrease by 2% compared to the annual-scale model, while in the CN60-noLM scenario, which does not include demand-side flexibility measures, it would decrease by 12%. For scenarios considering 2-timeslice (CN60-DN) and 32-timeslice (CN60-noHour) scenarios, power generation would decrease by 3% and 4%, respectively. Demand for dispatchable resources would rise sharply with considerable differences between scenarios, with pumped hydro growing by 43-135% under hourly scenarios, and CN60-noHour and CN60-DN would grow by 90% and 46% respectively. Grid-connected hydrogen production would increase by more than 45% when seasonal factors are taken into account, but only by 27% when only diurnal variations are considered.

(Manuscript, Line 180-182) By comparing the CN60 and CN60-noLM scenarios, a discernible shift is evident in the consumption patterns of adaptive loads, including EV charging and hydrogen production.

(Manuscript, Line 184-187) Model results suggest adoption of fast-charging devices would increase dramatically, projected to increase from 15% in 2019 to 80% by 2060 in CN60 while remaining stable in CN60-noLM.

(Manuscript, Line 194-197) Compared with the CN60-noLM scenario, strategic hydrogen production in the CN60 scenario has the potential to utilize surplus daytime

electricity, thereby mitigating rather than exacerbating power imbalances during peak demand periods.

(Manuscript, Line 235-236) Considering system operation constraints at the hourly level (CN60), the total system cost would rise by about 4% compared to the CN60-noTS scenario.

(Manuscript, Line 239-244) Net-zero transition in the CN60 scenario requires an increment annual investment by 50% compared to the NDC scenario (Fig. 8). Under the CN60 scenario, annual investment in VRE, energy storage, BECCS and hydrogen needs to be approximately 250, 30, 12 and 44 billion USD by 2060. Energy storage expenses would be 24% larger in the CN60 compared to the CN60-noTS by 2060.

Comment 3.6:

A further concern in this revised manuscript relates to the writing and overall structure. The authors have commendably added a great deal of information to clarify the questions raised by reviewers, which is much appreciated. However, the manuscript as a whole would benefit from further proofreading to ensure it is concise enough and better aligned with your research focus and the key conclusions emerging from the analysis. Especially in the Results section, the presentation of quantitative findings does not fully reflect the structure and insights expected from your three comparisons. In other words, the presentation could be more clearly organized or restructured to better convey the main findings.

Response:

Thank you very much for your suggestions, which will greatly improve the readability of our article. Our narrative logic follows “the need for more refined time granularity assessment in high VRE systems”—“power supply needs to be repositioned to address flexibility challenges”—“demand-side flexibility is crucial”—“high levels of supply-demand interaction address flexibility crises”, emphasizing the comparison between hourly scenarios and coarse-timeslice scenarios, considering the impact of orderly load (CN60 vs. CN60-noLM), and considering the impact of demand response and V2G (CN60-LM vs. CN60). Finally, a comprehensive assessment of cost-effectiveness is conducted.

We have restructured the Results around the three key comparisons. First, we revised the subheadings in the results section to make each subsection clearer and more progressive. Second, we deleted some background descriptive text that were not

directly related to flexibility, thereby making the paper more focused. Third, we centered around a small set of system-level metrics aligned with flexibility and feasibility.

Changes/additions to the manuscript:

(Manuscript, Line 140) Flexibility scarcity leading to power plant repositioning

(Manuscript, Line 162-165) Its operational focus is anticipated to transition from catering to peak loads to bolstering ramping capacity and accommodating nighttime loads, with over 35% of electricity demand at night being met by hydro.

(Manuscript, Line 174-176) This surge is coupled with an increase in peak load, projected to grow from 1.0 TW in 2019 to at least 2.5 TW in 2060. Peak loads could be even further in case power load fluctuates unpredictably.

(Manuscript, Line 184-187) Model results suggest adoption of fast-charging devices would increase dramatically, projected to increase from 15% in 2019 to 80% by 2060 in CN60 while remaining stable in CN60-noLM.

(Manuscript, Line 215-220) Pumped hydro capacity is expected to reach 285 GW by 2060, a level deemed feasible with a consistent annual growth rate of 3% (Fig. 4d). In the CN60 scenario, it is necessary to invest pumped hydro facilities in nearly all eligible candidate sites (368 GW). Even worse, the CN60-noLM scenario exhausts the potential of pumped hydro and requires the intensive construction of other long-term storage facilities (417 GW).

(Manuscript, Line 234) Financial implications of a feasible energy transition to net-zero

(Manuscript, Line 241-244) Under the CN60 scenario, annual investment in VRE, energy storage, BECCS and hydrogen needs to be approximately 250, 30, 12 and 44 billion USD by 2060. Energy storage expenses would be 24% larger in the CN60 compared to the CN60-noTS by 2060.

Comment 3.7:

Since the key focus of the paper is the flexibility of the power sector transition, and the sub-annual model does not show significant differences in transition pathways in broad terms, I recommend tightening this section. Keep the key results concise and highlight where the sub-annual modeling truly makes a difference.

Response:

Thank you very much for your suggestion, which has been very helpful in highlighting our views. We have significantly reduced the content of this section and moved the lengthy description of the energy transition to Supplementary Notes 1. In addition, for aesthetic and recognizability reasons, we have only shown the four hourly granularity scenarios in Figure 1, and the complete figure comparing the 7scenarios is shown in Supplementary Figure 15.

Changes/additions to the manuscript:

(Manuscript, Line 86-89) China aims for net-zero emissions by 2060 amid rising energy demand and sectoral transition (Fig. 1). China is projected to peak its fossil fuel and industrial CO_2 emissions at 12 Gt in 2025. Coal use will decline sharply post-2030, with renewables dominating power generation after 2035.

(Supplementary Notes 1 and Supplementary Figure 15)

Supplementary Note 1 China's energy transition towards carbon neutrality

China is projected to reach its peak in fossil fuel and industrial processes (FFI) carbon emissions around 2025 at 12 $GtCO_2$ (Supplementary Fig. 15a). Unlike the rapid coal-fired power plant (CFPP) phase-out pathway proposed by most IAMs, our study shows the ongoing expansion of CFPPs would persist as renewables are inadequate in addressing the incremental demand for electricity. After 2030, the energy system is expected to undergo a marked transition, culminating in the achievement of CO_2 net-zero emissions by 2060. Achieving the desired climate targets necessitates a precipitous decline in the coal usage in unabated CFPPs and energy-intensive industries, which would lead to formidable transition pressures (Supplementary Fig. 15b). Final energy consumption is projected to rise by 16% from 2020 to 2030, followed by a gradual decline leading up to 2060 (Supplementary Fig. 15c). The expansion of electricity applications would lead to changes in both the quantity and temporal distribution of electricity consumption (Supplementary Figs. 1-5). Renewables are anticipated to dominate electricity generation after 2035 (Supplementary Fig. 15d). This shift necessitates an accelerated transition within the power sector.

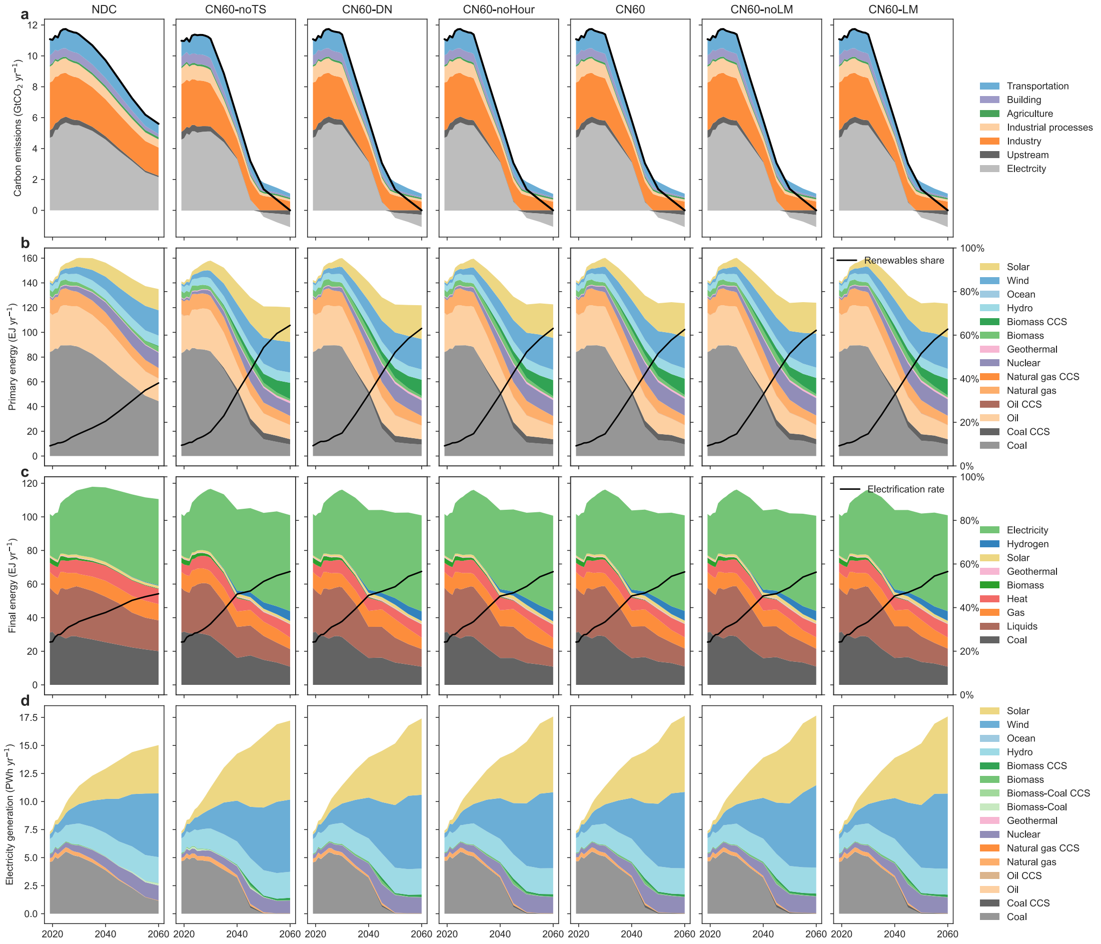

Supplementary Fig. 15 China's energy system decarbonization pathway for all scenarios

a Fossil fuel and industrial processes (FFI) CO_2 emissions by sector, **b** Primary energy mix by fuel, **c** Final energy mix by fuel, **d** Electricity generation by technology.

Comment 3.8:

Lines 114 – 118: "Fig. 2 explores the implications of considering sub-annual modeling across the dimensions of mitigation strategy, energy demand, energy supply, dispatchable resource, and cost & effort. The sub-annual analysis indicates that achieving the energy transition with a high VRE share and a high electrification rate would pose greater challenges than that from the annual perspective." This sentence appears to suggest that sub-annual analysis poses greater challenges to the energy transition itself, which is confusing. Sub-annual modelling is a change in the granularity of time, and while it may reveal operational challenges, it does not in itself create obstacles to transition pathways.

Response:

Thank you very much for pointing out this issue. What we want to express is that more refined modeling reveals challenges that previous models could not detect, rather than creating challenges. We have re-proofread the entire text and resolved any ambiguities that such issues may have caused.

Changes/additions to the manuscript:

(Manuscript, Line 101-110) Fig. 2 compares outcomes under different temporal granularity and flexibility management across mitigation strategy, energy demand, energy supply, dispatchable resources, and cost. Fig. 3 compares the results with prominent IAMs under a harmonized scenario framework with the same carbon budget constraints and net-zero targets. The finer temporal resolution reveals operational constraints that are not visible at coarse resolution which tighten feasible pathways for high VRE shares and fast electrification unless flexibility is enhanced accordingly. In addition, the distinct trajectories of thermal power, green hydrogen, and energy storage in China TIMES 2.0, which differ from other IAMs, clearly demonstrate the unique impact of system flexibility on long-term planning.

Comment 3.9:

Lines 134 – 136: "The scenario with sub-annual modelling would increase marginal abatement costs of CO2 by more than 9% and decrease total energy consumption by 3% in 2060 compared to that without sub-annual modelling." This has a similar issue: it implies the modeling choice causes these differences rather than reveals them through a more refined temporal lens. I suggest reframing to clarify.

Response:

Thank you very much for pointing out this ambiguous statement. We have corrected it in the new statement and explicitly pointed out that the difference in optimization results is due to the internalization of flexibility requirements and operational constraints.

Changes/additions to the manuscript:

(Manuscript, Line 113-117) When temporal variability is explicitly represented, the cost-optimal solution exhibits marginal abatement costs that are over 9% higher and total final energy about 3% lower by 2060 relative to a coarse-timeslice baseline. These differences arise because the model internalizes flexibility requirements and operational constraints that are muted at the annual scenario.

Comment 3.10:

The context in Lines 158 – 162 could be tightened.

Response:

We appreciate your feedback. We have refined our original statement and explained why the expansion of VRE leads to flexibility issues.

Changes/additions to the manuscript:

(Manuscript, Line 142-144) China has historically concentrated its flexibility resources on the supply side, primarily through thermal power and hydropower executing dispatch instructions.

Comment 3.11:

Focus more directly on results or metrics that clearly support the subtitle’s theme—i.e., highlighting the scarcity of flexibility in a high-VRE system.

Response:

Thank you very much for your comment. We first clarified the focus of this section, which is the shift in flexibility shortages on the power supply side due to the development of VRE. Therefore, we first revised the section title. Second, we explained at what stage flexibility shortages are likely to occur. Subsequently, we described the periods of intraday flexibility shortages indicated by the net load curve and discussed the future development patterns of thermal power, hydropower, and nuclear power. Finally, we discussed flexibility issues at the seasonal level.

Changes/additions to the manuscript:

(Manuscript, Line 140) Flexibility scarcity leading to power plant repositioning

(Manuscript, Line 141-142) During periods of ramping, generation output variations, net load fluctuations, and peak load hours, flexibility scarcity issues are likely to arise.

(Manuscript, Line 150-153) CFPP flexibility retrofits can significantly improve the dispatch performance of existing assets, partially alleviating the lack of flexibility (Supplementary Notes 2 and Supplementary Figure 16-17).

(Manuscript, Line 156-157) With the rapid spread of VRE, it is essential to rapidly scale up low-carbon flexibility resources.

(Manuscript, Line 161-165) Hydropower has gained significant importance in the high-VRE power system and is poised to become a central pillar of flexibility. Its

operational focus is anticipated to transition from catering to peak loads to bolstering ramping capacity and accommodating nighttime loads, with over 35% of electricity demand at night being met by hydro.

(Manuscript, Line 166-171) Seasonal pattens in PV and wind output reshape system balance configuration (Fig. 5d). In summer, PV will dominate, requiring massive electricity storage during midday hours and low-load operation capabilities for thermal power plants. In winter, wind dominance will increase intraday power load fluctuations and multi-hour deficits. These dynamics will increase the demand for fast-ramping capacity, short-duration storage, and demand flexibility to contain curtailment and maintain adequacy.

Comment 3.12:

Lines 183 - 187: "In the context of seasonal variations in electricity supply, summer is distinguished by the longest periods of daylight, resulting in PV generation contributing ~48% of the electricity mix. Conversely, during the winter and spring months, wind energy assumes a more prominent role..." This provides seasonal context on VRE availability, but the link to "flexibility scarcity" is unclear. Consider reframing this to focus on how these variations create flexibility challenges.

Response:

Thank you very much for reminding us to link seasonal variations with flexibility challenges. In our new statement, we have reframed the issue to show how seasonal PV/wind patterns translate into specific flexibility challenges (hourly fluctuations, low-severe oversupply, peak load hours, multi-hour deficits).

Changes/additions to the manuscript:

(Manuscript, Line 166-171) Seasonal pattens in PV and wind output reshape system balance configuration (Fig. 5d). In summer, PV will dominate, requiring massive electricity storage during midday hours and low-load operation capabilities for thermal power plants. In winter, wind dominance will increase intraday power load fluctuations and multi-hour deficits. These dynamics will increase the demand for fast-ramping capacity, short-duration storage, and demand flexibility to contain curtailment and maintain adequacy.

Comment 3.13:

Lines 189 - 191: "The CN60-noLM scenario demonstrates higher levels of storage

activity in comparison to the other two net-zero scenarios, indicating that orderly demand could alleviate power balancing challenges (Fig. 6a)." This content is more appropriate for the next section (Orderly demand easing power imbalance) and should be moved there for better flow.

Response:

Thank you very much for your suggestion. We have moved it to the next section and refined the content.

Changes/additions to the manuscript:

(Manuscript, Line 189-191) In the CN60-noLM scenario without demand-side participation, the load curve’s shape remains unchanged, thereby requiring higher storage capacity and activity (Fig. 6a).

Comment 3.14:

For the sections Orderly demand easing power imbalance and Addressing the flexibility crisis through demand-supply interaction, there is a similar issue of attempting to cover too many quantitative details, which results in a lack of clear focus in the discussion. I would suggest the authors to further refine the narrative structure in these sections to strengthen the logical flow and better highlight the key insights.

Response:

Thank you very much for pointing out the shortcomings in our expression. First, we made significant cuts to the text and quantitative details, removing content that was not closely related to the main theme of the article.

In the section titled “Orderly demand easing power imbalance,” we further highlighted the role of flexible loads such as orderly EV charging and orderly hydrogen production. Finally, we also explained the load fluctuations in different seasons.

In the section “Addressing the flexibility crisis through demand-supply interaction,” we discuss the two common forms of demand-side response (“shiftable load” and “curtailable load”) and V2G technology, which exemplify source-load interaction. Since such technologies are often used to smooth peak loads, we observe not only their activities but also their associated impact on energy storage construction.

Changes/additions to the manuscript:

(Manuscript, Line 176) Peak loads could be even further in case power load

fluctuates unpredictably.

(Manuscript, Line 177-178) Our study delineates the seasonal and intraday variations in power loads, a crucial aspect for integrating more challenging-to-dispatch power systems.

(Manuscript, Line 182-187) Under the CN60 scenario, results show EV would shift from charging primarily at night to charging during the day, utilizing PV power and alleviating excess electricity supply. Model results suggest adoption of fast-charging devices would increase dramatically, projected to increase from 15% in 2019 to 80% by 2060 in CN60 while remaining stable in CN60-noLM.

(Manuscript, Line 189-191) In the CN60-noLM scenario without demand-side participation, the load curve's shape remains unchanged, thereby requiring higher storage capacity and activity (Fig. 6a).

(Manuscript, Line 202-206) Significant increases in winter electricity consumption are expected in the future due to the growing popularity of electric heating technology, thereby narrowing seasonal differences in electricity consumption (Fig. 5b). Hydrogen produced in spring and autumn is stored for use in summer and winter, thereby achieving cross-seasonal energy storage (Fig. 7d).

(Manuscript, Line 215-220) Pumped hydro capacity is expected to reach 285 GW by 2060, a level deemed feasible with a consistent annual growth rate of 3% (Fig. 4d). In the CN60 scenario, it is necessary to invest pumped hydro facilities in nearly all eligible candidate sites (368 GW). Even worse, the CN60-noLM scenario exhausts the potential of pumped hydro and requires the intensive construction of other long-term storage facilities (417 GW).

(Manuscript, Line 227-229) The energy price increase resulting from emission reduction could incentivize demand-side load reduction, which is also an important means of demand-side response.

Reviewer 4

I co-reviewed this manuscript with one of the reviewers who provided the listed reports. This is part of the Nature Communications initiative to facilitate training in peer review and to provide appropriate recognition for Early Career Researchers who co-review manuscripts.

Response:

Thanks very much for giving us helpful comments.
